# Orientational Self-Sorting in Octahedral Coordination Cages Based on Small, Heteroditopic N‑Donor Ligands

**DOI:** 10.1021/acs.inorgchem.6c02256

**Published:** 2026-07-27

**Authors:** Jean de Montmollin, Cédric Przybylski, Jean-Yves Salpin, Daniel Ortiz, Pol Gorrea-Acín, Atena B. Solea, Farzaneh Fadaei-Tirani, Kay Severin

**Affiliations:** † Institute of Chemical Sciences and Engineering, École Polytechnique Fédérale de Lausanne (EPFL), 1015 Lausanne, Switzerland; ‡ Université Paris-Saclay, Univ. Evry, CY Cergy Paris Université, CNRS, LAMBE, 91025 Evry-Courcouronnes, France

## Abstract

Coordination cages based on heteroditopic N-donor ligands can potentially form isomers, which differ in the relative orientation of the ligands. We have explored the construction of octahedral cages using small low-symmetry N-donor ligands (L). When the ligands were combined with [Pd­(CH_3_CN)_4_]­(BF_4_)_2_, hexanuclear Pd_6_L_12_-type cages were obtained as a mixture of isomers, as evidenced by NMR spectroscopy and single-crystal X-ray crystallography. Attempts to resolve the isomers by cyclic ion mobility-mass spectrometry (cIM-MS) are described. Structurally defined cages of the general formula [Pt_2_Pd_4_Cl_8_L_8_]­(BF_4_)_4_ were formed in kinetically controlled reactions of homoleptic [PtL_4_]­(BF_4_)_2_ complexes with PdCl_2_(CH_3_CN)_2_. Crystallographic analyses of the products have revealed antipodal Pt^2+^ centers, the pyridine or pyrimidine donor groups of the bridging ligands being exclusively bound to the four equatorial PdCl_2_ complexes.

## Graphical abstract

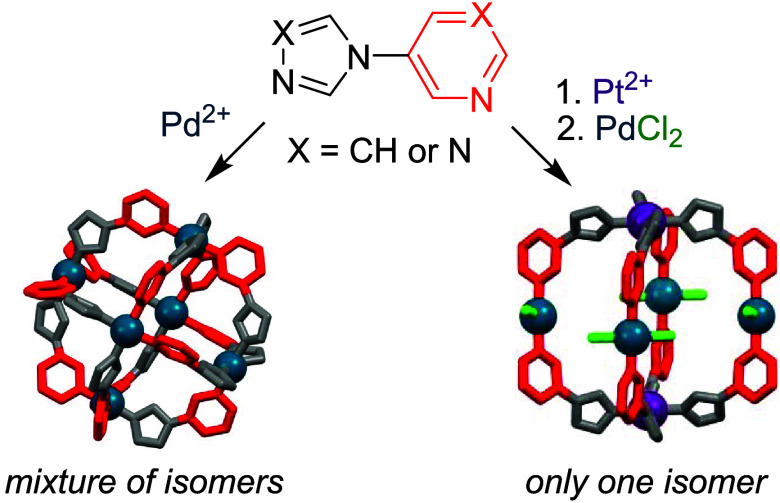

## Introduction

Coordination cages based on bridging, ditopic ligands tend to display high symmetry.^1–6^ While highly regular structures are aesthetically pleasing, they are not necessarily optimal for potential applications.^7^ One strategy for reducing the symmetry of the final assembly is the use of heteroditopic ligands.^8,9^ However, this strategy comes with a challenge: the self-assembly process can produce multiple isomers. This point is illustrated in Figure 1a for widely studied Pd_2_L_4_-type complexes.^10,11^ Ligands with two distinct donor groups can potentially give four isomers. These isomers differ in the relative orientation of the bridging ligands. Our group and several others have shown that *orientational self-sorting* can be achieved through appropriate ligand design.^12–34^

**Figure 1:**
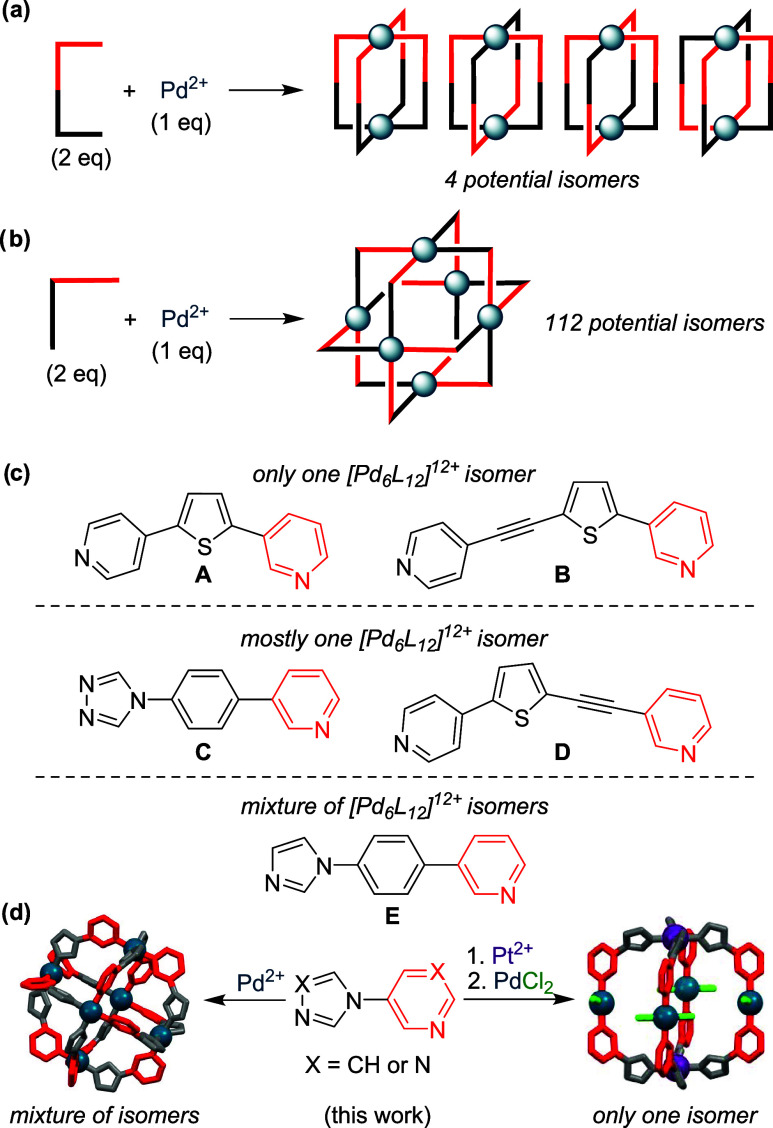
(a) Pd_2_L_4_-type cages based on heteroditopic ligands can potentially form four isomers. (b) Pd_6_L_12_-type cages based on heteroditopic ligands can potentially form 112 isomers, enantiomers excluded. Only one isomer is depicted. (c) Structures of heteroditopic ligands, which give rise to Pd_6_L_12_-type cages. (d) Synthesis of Pd_6_L_12_- and Pt_2_Pd_4_L_8_-type cages described in this work.

For metallosupramolecular complexes with more than four heteroditopic ligands, the number of possible isomers increases rapidly.^31–33^ In the case of Pd_6_L_12_-type complexes, for example, there are already 112 potential isomers, excluding enantiomers (Figure 1b). Despite the large number of possible isomers, the self-assembly process can still lead to a high degree of orientational self-sorting. When the dipyridyl ligands **A** or **B** were combined with Pd^2+^, structurally defined Pd_6_L_12_ complexes with exclusive *cis* coordination at the six vertices were observed (Figure 1c).^32,34^ Likewise, the use of the heteroditopic ligands **C** or **D** resulted predominantly in the formation of *cis*-isomers, with only minor amounts of side products.^34^ In the case of ligand **E**, however, two different Pd_6_L_12_ isomers could be characterized crystallographically, and the NMR data suggested the formation of a mixture of products.^34^

In continuation of these investigations, we have studied the formation of octahedral coordination cages based on heteroditopic ligands containing one pyridine or pyrimidine donor group and one imidazole or triazole donor group (Figure 1d). The ligands **L1** and **L3** represent structural analogs of the ligands **C** and **E**, with the phenylene spacer being replaced by a direct C–N link. We were interested in how the reduced size and flexibility would influence the self-assembly process. Thermodynamically controlled reactions of these ligands with Pd^2+^ were found to give mixtures of isomers, some of which could be characterized crystallographically (Figure 1d). In contrast, we were able to obtain octahedral cages as single isomers by stepwise reaction of the ligands with first Pt^2+^ and then PdCl_2_.

## Results and Discussion

For our investigations, we have employed the heteroditopic N-donor ligands **L1**–**L3**, the structures of which are depicted in Scheme 1. From a coordination chemistry point of view, these ligands can be regarded as shorter analogs of ligands **C** and **E** (Figure 1), since the coordinate vectors of the two N-donor groups cross at the same angle when a flat ligand conformation is considered. However, it should be noted that the ligand bent angle is not fixed, as the relative orientation of the coordinate vectors changes when the two heterocycles adopt noncoplanar conformations. With only one rotatable C–C single bond, the ligands **L1**–**L3** have an overall reduced conformational flexibility when compared to **C** and **E**. Alongside geometric considerations, the donor strengths of the heterocycles should also be taken into account. Strong donors such as imidazoles can result in the formation of kinetically trapped species, thereby hampering efficient self-sorting.^34^

**Scheme 1:**
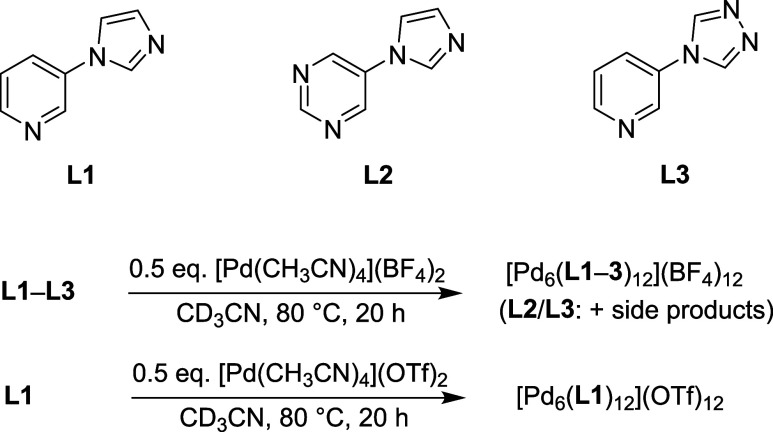
Synthesis of the Hexanuclear Coordination Cages [Pd_6_(**L1**–**3**)_12_]­(BF_4_)_12_ and [Pd_6_(**L1**)_12_]­(OTf)_12_

First, we examined if the ligands **L1**–**L3** could be used to construct Pd_6_L_12_-type cages. Two equivalents of the respective ligand were combined with one equivalent of [Pd­(CH_3_CN)_4_]­(BF_4_)_2_ in CD_3_CN. The mixtures were then heated to 80 °C for 20 h. To probe for anion effects, we have also combined ligand **L1** with [Pd­(CH_3_CN)_4_]­(OTf)_2_.

The ^1^H NMR spectrum of the mixture obtained with ligand **L1** showed broad, ill-defined peaks (Figure 2a, top spectrum). The peak-broadening was not caused by dynamic effects, as evidenced by the fact that the spectrum did not change substantially over a temperature range of 25 to 70 °C (see the Supporting Information (SI), Figure S16). The ^1^H NMR spectrum obtained from the mixture of **L1** and [Pd­(CH_3_CN)_4_]­(OTf)_2_ was similar to that obtained with [Pd­(CH_3_CN)_4_]­(BF_4_)_2_, suggesting that the anion had a minor influence on the reaction (Figure S7). Reactions with the ligands **L2** or **L3** gave ^1^H NMR spectra with broad peaks and additional sharper signals (Figure 2a). The DOSY NMR spectra (Figures S11 and S14) suggest that the sharper peaks do not originate from the cages, but from smaller assemblies. Some of the signals can be attributed to [Pd­(**L2**/**3**)_4_]­(BF_4_)_2_ complexes (see Figure S17 and HRMS data in Figures S18–S20).

**Figure 2:**
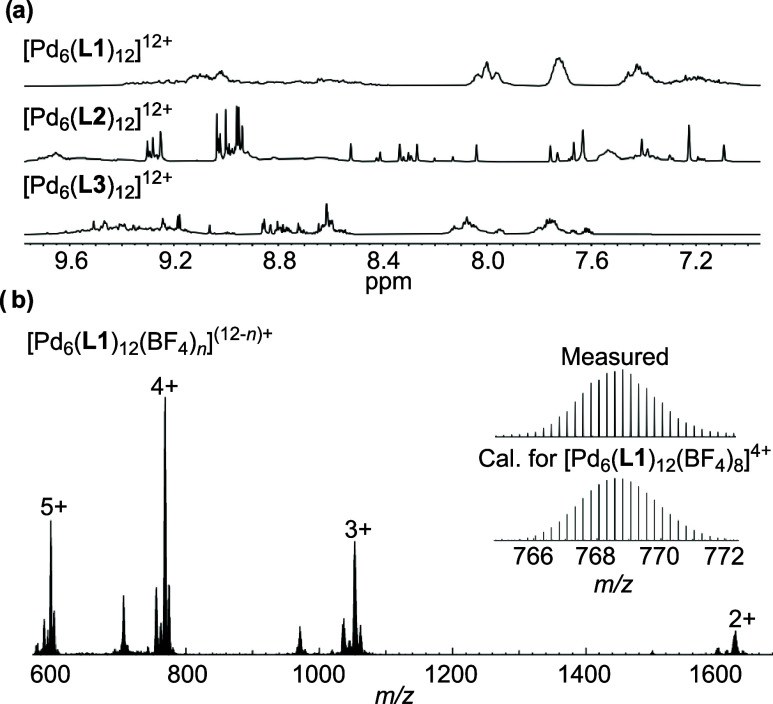
(a) ^1^H NMR spectra (800 MHz, CD_3_CN) of the cages [Pd_6_(**L1**–**3**)_12_]­(BF_4_)_12_. (b) High-resolution ESI mass spectrum of [Pd_6_(**L1**)_12_]­(BF_4_)_12_.

Since the NMR data were not very informative, we analyzed the mixtures by high-resolution electrospray ionization (ESI) mass spectrometry. The resulting spectra confirmed the formation of the expected hexanuclear cages, showing peaks for species of the formula [Pd_6_(**L1**–**3**)_12_(BF_4_)_*n*_]^(12–*n*)+^ (Figures 2b and S12 and S15). Taken together, these analytical results indicate that the reactions yielded hexanuclear cages, albeit as mixtures of isomers.

The hypothesis that the Pd_6_L_12_ cages can form different isomers was supported by single-crystal XRD analyses of [Pd_6_(**L1**)_12_]­(BF_4_)_12_, [Pd_6_(**L1**)_12_]­(OTf)_12_, [Pd_6_(**L2**)_12_]­(BF_4_)_12_, and [Pd_6_(**L3**)_12_]­(BF_4_)_12_. Distinct structures were observed. The cages [Pd_6_(**L1**)_12_]­(BF_4_)_12_ (Figure 3a) and [Pd_6_(**L1**)_12_]­(OTf)_12_ (see the SI, Figure S63) display approximate *S*_6_ symmetry with a *cis*-configuration at all six metal centers. This ligand arrangement corresponds to what was observed for cages based on the ligands **A**–**D** (Figure 1). A closely related geometry with *cis*-coordinated metal centers was also found for a hexanuclear copper cage based on ligand **L3**.^35^ In contrast, the crystallographically characterized Pd cage with **L3** shows approximate *C*_4h_ symmetry, with two types of metal centers: two [Pd­(triazole)_4_]^2+^ complexes and four [Pd­(pyridine)_3_(triazole)]^2+^ complexes (Figure 3b). Yet another structure was observed for [Pd_6_(**L2**)_12_]­(BF_4_)_12_. Disorder on two ligand positions gave rise to two possible isomers (Figure 3c,3d), showing approximate *C*_s_ symmetry. Both isomers feature two *cis*-[Pd­(pyrimidine)_2_(imidazole)_2_]^2+^ complexes, two *trans*-[Pd­(pyrimidine)_2_(imidazole)_2_]^2+^ complexes, one [Pd­(pyrimidine)­(imidazole)_3_]^2+^ complex, and one [Pd­(pyrimidine)_3_(imidazole)]^2+^ complex. However, the relative position of these complexes is different for the two isomers (Figure 3c).

**Figure 3:**
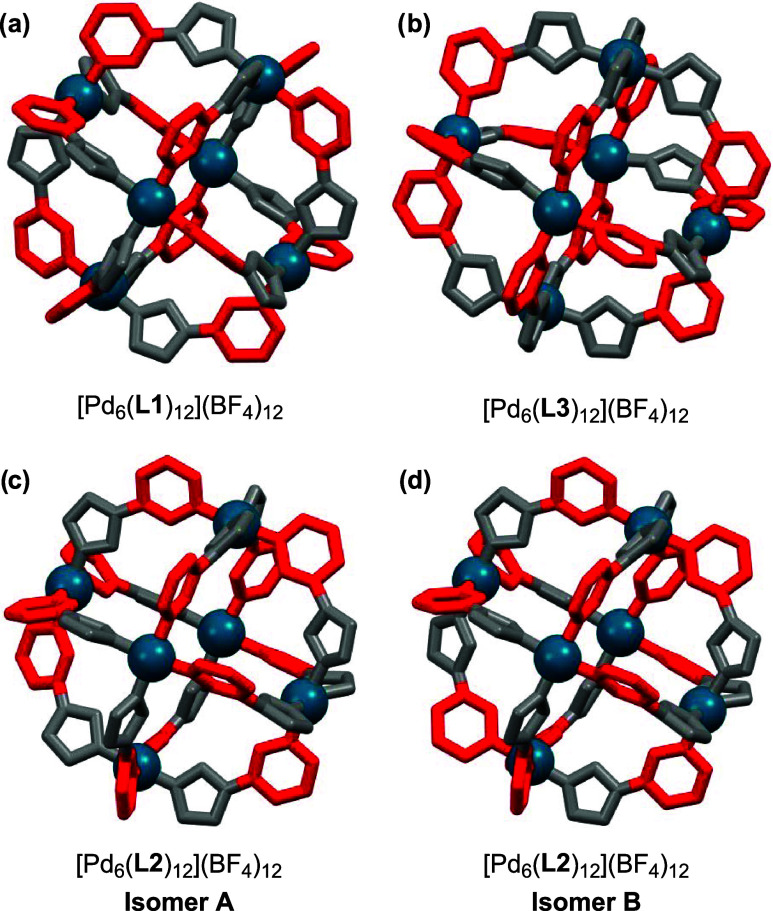
Molecular structures of the cages [Pd_6_(**L1**)_12_]­(BF_4_)_12_ (a), [Pd_6_(**L3**)_12_]­(BF_4_)_12_ (b), and [Pd_6_(**L2**)_12_]­(BF_4_)_12_ (c and d, two possible isomers due to disorder) in the solid state, as determined by single-crystal XRD. Hydrogen atoms and anions are not depicted. The color coding of the ligand aims to distinguish the two different donor groups.

While the relative orientation of the bridging ligands can vary, the cages based on **L1**, **L2**, and **L3** have similar sizes, with distances between the Pd atoms at opposite vertices of approximately 11.8 Å. It is worth noting that this value is low compared to what has been reported for other Pd_6_L_12_-type cages.^32,34,36–44^ Octahedral cages with a shorter Pd–Pd distance are only known for Pd_6_L_8_-type systems based on tritopic ligands (10.6 Å).^45^

The characterization of different isomers for cages based on structurally closely related ligands suggests that these isomers are close enough in energy so that they can coexist in solution. A plausible explanation for the ill-defined NMR spectra of the assemblies [Pd_6_(**L1**–**3**)_12_]­(BF_4_)_12_ and [Pd_6_(**L1**)_12_]­(OTf)_12_ is therefore the presence of multiple isomers. The lack of selectivity in the self-assembly of the cages [Pd_6_(**L1**–**3**)_12_]­(BF_4_)_12_ could be related to the fact that the two donor groups in **L1**–**3** are not well distinguished from a purely geometrical point of view. As outlined previously,^34^ orientational self-sorting in M_6_L_12_-type cages is expected for ligands with a bent angle of approximately 90° and a pronounced ‘asymmetry’, meaning that the distances from the two donor atoms to the intersection point of their coordination vectors are significantly different. This criterion is not met by **L1**–**3**. For **L1** and **L2**, kinetic factors may also contribute, as the strong binding affinity of the imidazole donors could promote the formation of kinetically trapped assemblies.

In order to obtain further information, we conducted high-resolution cyclic ion mobility-mass spectrometry (cIM-MS) analyses of the reaction mixtures. cIM-MS separates ions based on their size, shape, and charge by sending them through a gas-filled mobility ring under an electric field, where more compact ions travel faster than extended ones.

While ion mobility mass spectrometry has been used with great success in metallosupramolecular chemistry,^46–48^ we anticipated that the resolution of structurally closely related Pd_6_L_12_ isomers would represent a challenging task.

First, we examined the isomer compositions of previously reported cages [Pd_6_ **C**_12_]­(BF_4_)_12_ and [Pd_6_ **E**_12_]­(BF_4_)_12_.^34^ NMR analyses had shown that [Pd_6_ **C**_12_]­(BF_4_)_12_ is primarily present as a single isomer (*cis*-isomer), whereas [Pd_6_ **E**_12_]­(BF_4_)_12_ forms a mixture of multiple isomers. The cIM-MS mobilograms are in accordance with this assumption, showing one main peak for [Pd_6_ **C**_12_]­(BF_4_)_12_ (Figures 4a, S49, and S50) and several peaks for [Pd_6_ **E**_12_]­(BF_4_)_12_ (Figures 4b, S51, and S52).

**Figure 4:**
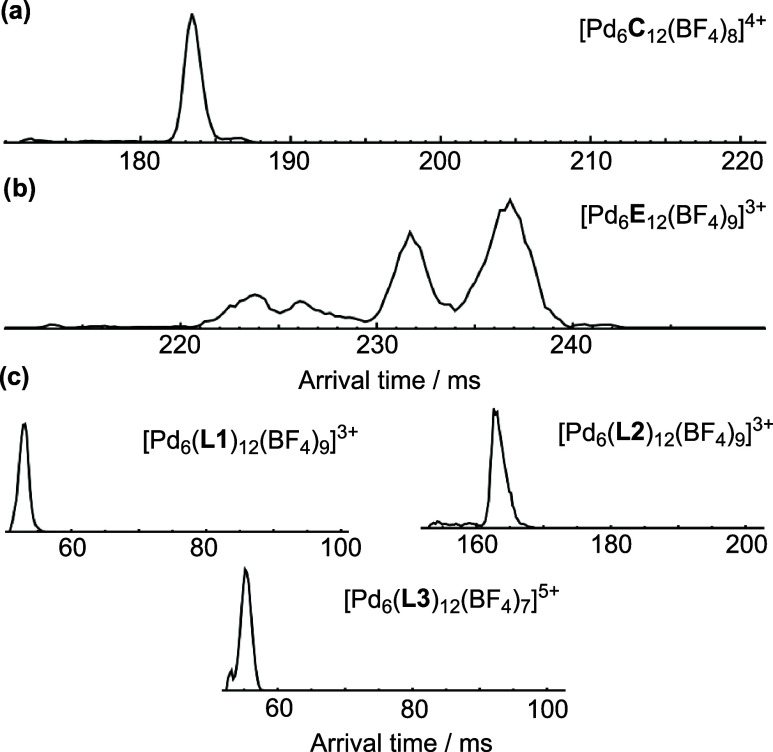
cIM-MS mobilograms of [Pd_6_ **C**_12_(BF_4_)_8_]^4+^ after 12 passes (a), of [Pd_6_ **E**_12_(BF_4_)_9_]^3+^ after 20 passes (b), and of [Pd_6_(**L1–3**)_12_(BF_4_)_*n*_]^(12+*n*)+^ (*n* = 7 or 9) after 12 passes (c).

Next, solutions of the smaller cages [Pd_6_(**L1–3**)_12_]­(BF_4_)_12_ were investigated. These cages were found to be less stable, resulting in the detection of only a few charged states and less “clean” spectra (see Figures S53, S55, and S57). For all three cages, the cIM-MS mobilograms showed one dominant peak (see Figures 4c, S56 and S58). The unequivocal separation of multiple isomers, as observed for [Pd_6_ **E**_12_]­(BF_4_)_12_, was not achieved.

With the goal of preparing structurally defined octahedral complexes based on the ligands **L1**–**L3**, we next investigated a stepwise synthetic approach. First, we prepared the homoleptic Pt^II^ complexes [Pt­(**L1**–**3**)_4_]­(BF_4_)_2_ by reaction of PtCl_2_(DMSO)_2_ with AgBF_4_ in CH_3_CN/DMSO (1:1), followed by addition of the respective ligand (4 equiv) (Scheme 2).

**Scheme 2:**
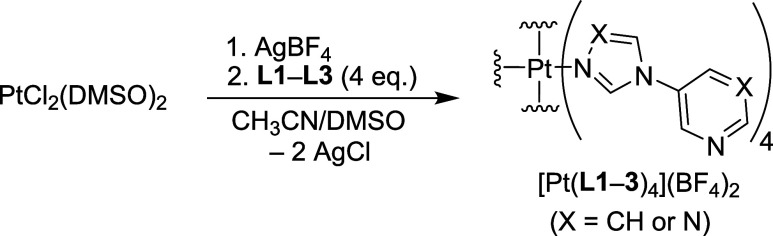
Synthesis of the Complexes [Pt­(**L1**–**3**)_4_]­(BF_4_)_2_

Crystallographic analyses of the complexes [Pt­(**L1**)_4_]­(BF_4_)_2_, [Pt­(**L2**)_4_]­(BF_4_)_2_, and [Pt­(**L3**)_4_]­(BF_4_)_2_ showed that the ligands are coordinated to the Pt center via the imidazole or triazole groups. The preferential complexation of the imidazole groups in [Pt­(**L1**)_4_]­(BF_4_)_2_ and [Pt­(**L2**)_4_]­(BF_4_)_2_ is in line with the higher basicity of these N-donors when compared to the pyridine or pyrimidine groups. In contrast, the coordination of **L3** to Pt^2+^ via the triazole group was unexpected, because the pyridine site is substantially more basic than the triazole group. A possible explanation for the preferential coordination of the triazole group in [Pt­(**L3**)_4_]­(BF_4_)_2_ could be the reduced steric repulsion between the triazole moieties around the Pt^2+^ center.

Due to the presence of four noncoordinated pyridine/pyrimidine groups, the complexes [Pt­(**L1**–**3**)_4_]­(BF_4_)_2_ can serve as metalloligands.^49–53^ As a linking metal complex, we decided to use PdCl_2_.^54–58^ When a mixture of [Pt­(**L1**)_4_]­(BF_4_)_2_ and PdCl_2_(CH_3_CN)_2_ in CD_3_CN was heated for 2 h at 80 °C, the formation of a defined complex was observed by ^1^H NMR spectroscopy (Figure 5), DOSY spectroscopy, and high-resolution ESI mass spectrometry (see the SI, Figures S37 and S38). A similar result was obtained when the reaction was performed in weakly coordinating CD_3_NO_2_. The assignment of the imidazole peaks could not be corroborated by COSY and NOESY NMR (see Figures S35 and S36).

**Figure 5:**
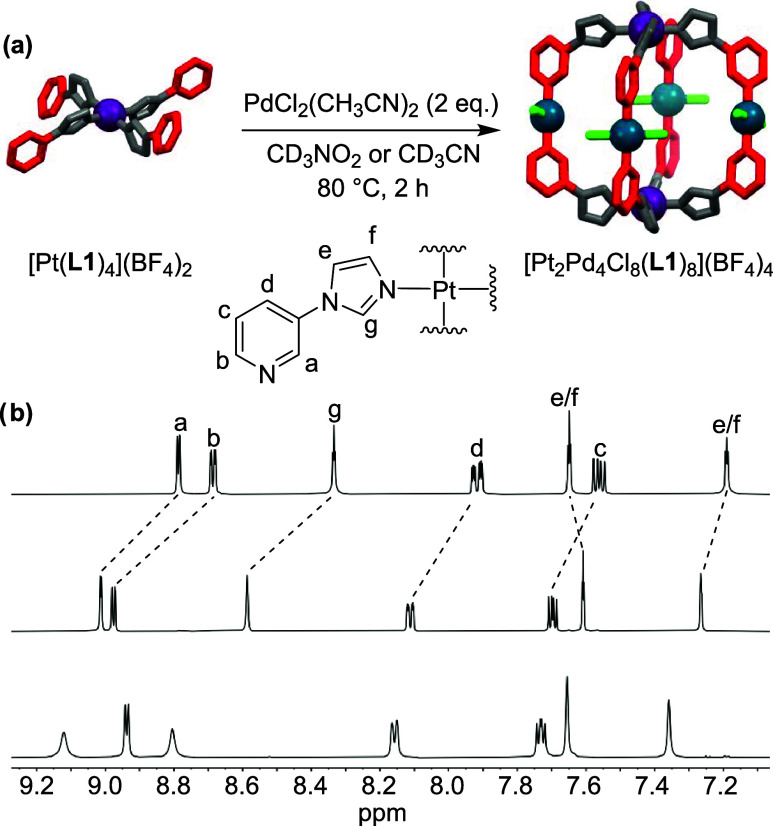
(a) Synthesis of cage [Pt_2_Pd_4_Cl_8_(**L1**)_8_]­(BF_4_)_4_, along with depictions of the molecular structures of [Pt­(**L1**)_4_]^2+^ and [Pt_2_Pd_4_Cl_8_(**L1**)_8_]^4+^, as determined by single-crystal XRD. (b) ^1^H NMR spectra of [Pt­(**L1**)_4_]­(BF_4_)_2_ in CD_3_CN (top, 400 MHz), of [Pt_2_Pd_4_Cl_8_(**L1**)_8_]­(BF_4_)_4_ in CD_3_CN (middle, 600 MHz), and of [Pt_2_Pd_4_Cl_8_(**L1**)_8_]­(BF_4_)_4_ in CD_3_NO_2_ (bottom, 600 MHz) with a peak assignment.

Single crystals of the product were obtained by slow vapor diffusion of diisopropyl ether into a solution of the cage in acetonitrile. The results of crystallographic analysis showed that the heterometallic cage [Pt_2_Pd_4_Cl_8_(**L1**)_8_]­(BF_4_)_4_ had formed.^59–61^

The heterometallic cage [Pt_2_Pd_4_Cl_8_(**L2**)_8_]­(BF_4_)_4_ was prepared as described for [Pt_2_Pd_4_Cl_8_(**L1**)_8_]­(BF_4_)_4_, with CD_3_NO_2_ as the solvent (Figure 6). Because the pyrimidine group in **L2** is less donating than the pyridine group in **L1**, the less coordinating solvent CD_3_NO_2_ was employed to maintain kinetic control. High-resolution ESI mass spectrometry confirmed the presence of the targeted cage (Figure 6c). A strong signal for the mononuclear complex [Pt­(**L2**)_4_]^2+^ was also observed. According to the ^1^H NMR data, this complex was not present in larger amounts in the reaction mixture (Figure 6b). Therefore, it is likely formed during the MS analysis.

**Figure 6:**
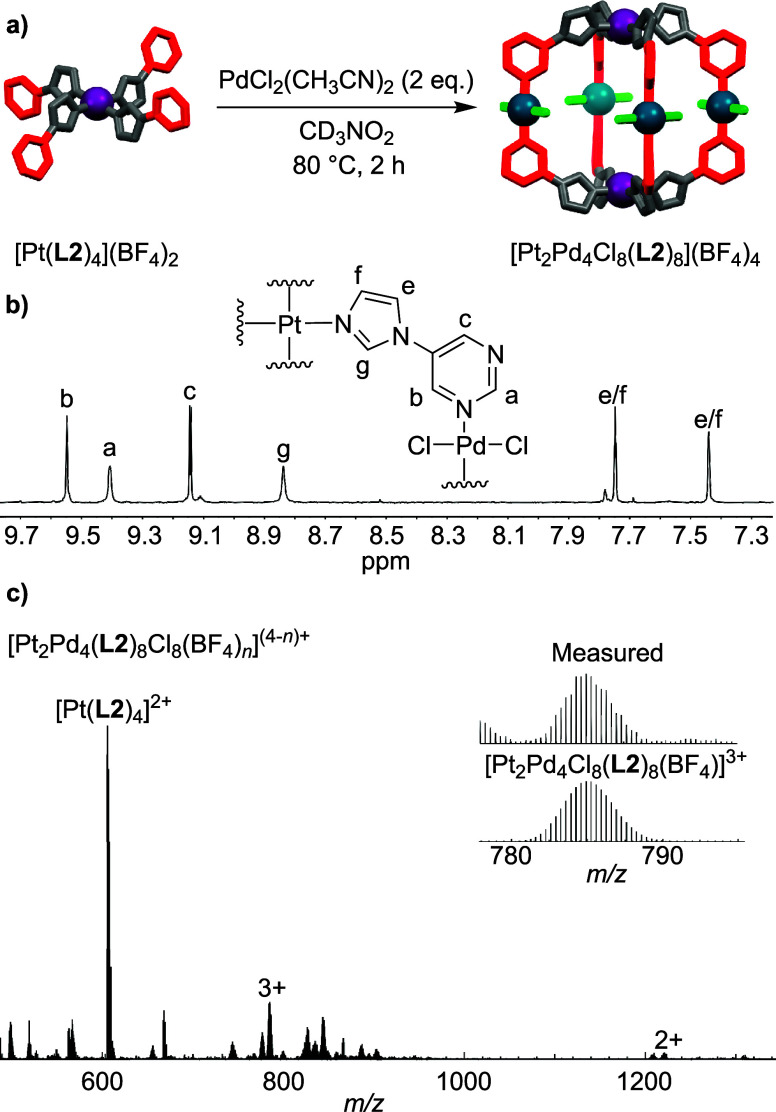
(a) Synthesis of cage [Pt_2_Pd_4_Cl_8_(**L2**)_8_]­(BF_4_)_4_, along with depictions of the molecular structures of [Pt­(**L2**)_4_]^2+^ and [Pt_2_Pd_4_Cl_8_(**L2**)_8_]^4+^, as determined by single-crystal XRD. (b) ^1^H NMR spectrum (600 MHz) of [Pt_2_Pd_4_Cl_8_(**L2**)_8_]­(BF_4_)_4_ in CD_3_NO_2_. (c) High-resolution ESI mass spectrum of the product.

Single crystals of [Pt_2_Pd_4_(**L2**)_8_Cl_8_]­(BF_4_)_4_ were obtained by slow (3 weeks) vapor diffusion of ^*i*^Pr_2_O into a solution of the cage in CD_3_NO_2_. The XRD analysis revealed a structure analogous to that of [Pt_2_Pd_4_Cl_8_(**L1**)_8_]­(BF_4_)_4_, with two [Pt­(imidazole)_4_]^2+^ complexes in apical position, and four [PdCl_2_(pyrimidine)_2_]^2+^ complexes in equatorial position (Figure 6a).

Lastly, [Pt­(**L3**)_4_]­(BF_4_)_2_ was combined with PdCl_2_(CH_3_CN)_2_ in CD_3_NO_2_ at room temperature. After 1 h at room temperature, a single set of peaks, distinct from the complex [Pt­(**L3**)_4_]­(BF_4_)_2_, were detected (Figure 7). A diffusion coefficient of 5.40 × 10^–10^ m^2^ s^–1^ was found by DOSY analysis, closely matching the values for [Pt_2_Pd_4_Cl_8_(**L1**)_8_]­(BF_4_)_4_ and [Pt_2_Pd_4_Cl_8_(**L2**)_8_]­(BF_4_)_4_ (5.40 and 5.30 × 10^–10^ m^2^ s^–1^, respectively, see Figures S37, S43, and S47). High-resolution ESI mass spectrometry further confirmed the presence of [Pt_2_Pd_4_Cl_8_(**L3**)_8_]­(BF_4_)_4_ (Figure S48).

**Figure 7:**
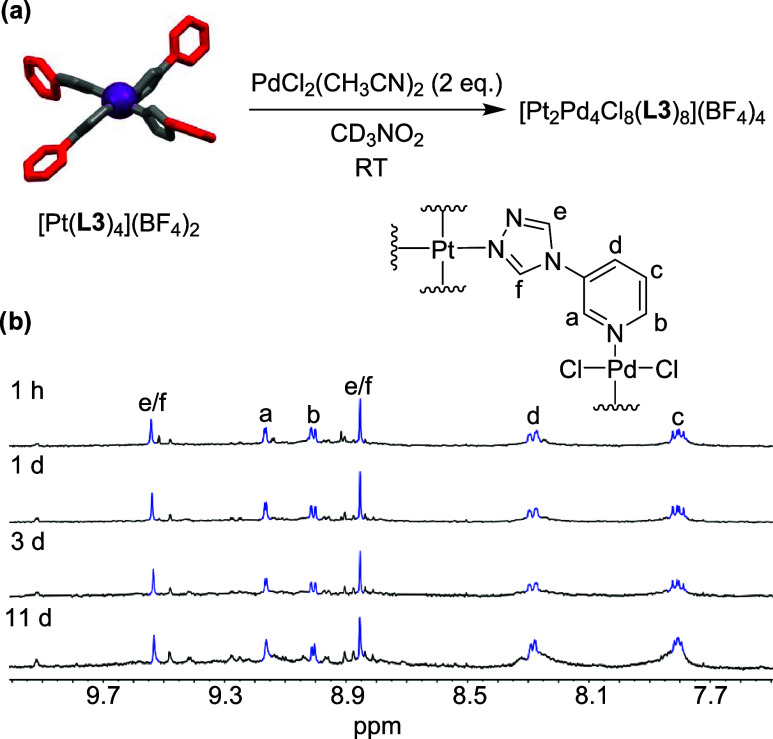
(a) Synthesis of cage [Pt_2_Pd_4_Cl_8_(**L3**)_8_]­(BF_4_)_4_, along with a depiction of the molecular structures of [Pt­(**L3**)_4_]^2+^, as determined by single-crystal XRD. (b) ^1^H NMR spectrum (600 MHz (11 d), 400 MHz (other)) of a mixture of [Pt­(**L3**)_4_]­(BF_4_)_2_ and PdCl_2_(CH_3_CN)_2_ in CD_3_NO_2_ after different time intervals.

The heterometallic cage [Pt_2_Pd_4_Cl_8_(**L3**)_8_]­(BF_4_)_4_ was found to display a reduced kinetic stability when compared to the analogous cages based on **L1** and **L2**. At room temperature, the slow conversion into new complexes was observed by ^1^H NMR spectroscopy (Figure 7b). The higher propensity of [Pt_2_Pd_4_Cl_8_(**L3**)_8_]­(BF_4_)_4_ to undergo ligand rearrangements can likely be linked to the diminished donor strength of the triazole heterocycle in **L3**.

## Conclusions

Pd_6_L_12_-type cages based on heteroditopic N-donor ligands can potentially form a vast number of structural isomers that differ in the relative orientation of the bridging ligands. Earlier work had shown that structurally defined complexes can be obtained with certain ligands. For the present study, we have examined the formation of cages using the heteroditopic N-donor ligands **L1**–**L3**. Compared to the ligands used in previous studies, the ligands **L1**–**L3** are relatively small, and the two donor groups are not well-differentiated from a purely geometric point of view.^34^

When the ligands **L1**–**L3** were combined with [Pd­(CH_3_CN)_4_]­(BF_4_)_2_, Pd_6_L_12_-type cages were obtained as evidenced by mass spectrometry. The complex NMR spectra of these cages suggest the presence of multiple isomers. Accordingly, we were able to characterize different cage isomers by single-crystal XRD.

Structurally defined heterometallic cages of the general formula [Pt_2_Pd_4_Cl_8_L_8_]­(BF_4_)_4_ were formed in kinetically controlled reactions of homoleptic [PtL_4_]­(BF_4_)_2_ complexes with PdCl_2_(CH_3_CN)_2_ (L = **L1**, **L2**, or **L3**). The combination of strongly donating imidazole groups and inert platinum precursor salts was found to be crucial in retaining the targeted isomer over time.

Overall, our work provides new insight into both the scope and the limitations of orientational self-sorting in coordination cages. Specifically, it highlights the structural features and conditions under which selective ligand orientation can be achieved, as well as the factors that lead to mixtures of isomers or reduced selectivity. The findings provide further guidance for the design of low-symmetry coordination cages with defined architectures.

## Experimental Section

### General

All chemicals were obtained from commercial sources and used without further purification unless stated otherwise. The ligands **L1**,^31^ **L2**,^62^ and **L3** ^63^ were prepared according to (or in analogy to) procedures described in the literature. NMR spectra were measured on a Bruker Avance III HD spectrometer (^1^H: 400 MHz) equipped with a BBFO-Plus_*z*_ 5 mm probe, a Bruker Avance III HD spectrometer (^1^H: 600 MHz) equipped with a CPPBBO_*z*_ 5 mm probe or a Bruker Avance II HD spectrometer (^1^H: 800 MHz) equipped with a CPTCI_XYZ_ 5 mm probe. High-resolution mass spectrometry experiments were carried out using a hybrid ion trap-Orbitrap Fourier transform mass spectrometer, Orbitrap Elite (Thermo Scientific) equipped with a TriVersa Nanomate (Advion) nanoelectrospray ionization source. The cIM-MS analyses were performed on a SELECT SERIES Cyclic Ion Mobility Mass Spectrometer (Waters, Manchester, UK), controlled via MassLynx software.

### Syntheses

#### [Pd_6_(**L1)**_12_]­(BF_4_)_12_

**L1** (2.0 eq., 2.4 μmol, 60 μL of a 40 mM stock solution in CD_3_CN), 500 μL CD_3_CN, and [Pd­(CH_3_CN)_4_]­(BF_4_)_2_ (1.0 eq., 1.2 μmol, 40 μL of a 30 mM stock solution in CD_3_CN) were added to an NMR tube and heated at 80 °C for 20 h to give a solution of cage [Pd_6_(**L1**)_12_]­(BF_4_)_12_. [Pd_6_(**L1**)_12_]­(OTf)_12_ was obtained similarly by heating a mixture of Pd­(OTf)_2_ and ligand **L1** at 86 °C overnight. A stock solution of Pd­(OTf)_2_ was obtained by a reaction of PdCl_2_(CH_3_CN)_2_ with AgOTf in CD_3_CN, followed by removal of AgCl with a syringe filter.

#### [Pd_6_(**L2**)_12_]­(BF_4_)_12_

**L2** (2.0 eq., 2.4 μmol, 60 μL of a 40 mM stock solution in CD_3_CN), 500 μL CD_3_CN, and [Pd­(CH_3_CN)_4_]­(BF_4_)_2_ (1.0 eq., 1.2 μmol, 40 μL of a 30 mM stock solution in CD_3_CN) were added to an NMR tube and heated at 80 °C for 20 h to give a solution of cage [Pd_6_(**L2**)_12_]­(BF_4_)_12_.

#### [Pd_6_(**L3**)_12_]­(BF_4_)_12_

**L3** (2.0 eq., 2.4 μmol, 60 μL of a 40 mM stock solution in CD_3_CN), 500 μL CD_3_CN, and [Pd­(CH_3_CN)_4_]­(BF_4_)_2_ (1.0 eq., 1.2 μmol, 40 μL of a 30 mM stock solution in CD_3_CN) were added to an NMR tube and heated at 80 °C for 20 h to give a solution of cage [Pd_6_(**L3**)_12_]­(BF_4_)_12_.

#### [Pt­(**L1**)_4_]­(BF_4_)_2_

A solution of *cis*-PtCl_2_(DMSO)_2_ in DMSO-*d*_6_ (80 mM, 2 mL, 160 μmol) was mixed with a solution of AgBF_4_ in dry acetonitrile (160 mM, 2 mL). After 30 min, the precipitate was removed using a syringe filter to yield a 40 mM solution of [Pt­(CH_3_CN)_4_]­(BF_4_)_2_ in CH_3_CN:DMSO-*d*_6_ (1:1). Ligand **L1** (20 mg, 138 μmol, 4 equiv), acetonitrile (861 μL), and [Pt­(CH_3_CN)_4_]­(BF_4_)_2_ (40 mM, 861 μL, 34.4 μmol, 1 equiv) were combined and the mixture was heated at 80 °C overnight. The mixture was allowed to cool to RT and was filtered using a syringe filter (0.22 μm). The product was precipitated with toluene (13 mL) and washed with toluene (14 mL) using a centrifuge. The product was dried in air overnight and then dried under reduced pressure at RT for 1 h. [Pt­(**L1**)_4_]­(BF_4_)_2_ was obtained as a white powder (24.0 mg, 25.2 μmol, 73%). ^1^H (400 MHz, CD_3_CN) δ 8.79 (dd, *J* = 2.7, 0.8 Hz, 4H), 8.69 (dd, *J* = 4.8, 1.4 Hz, 4H), 8.33 (t, *J* = 1.4 Hz, 4H), 7.92 (ddd, *J* = 8.3, 2.8, 1.4 Hz, 4H), 7.65 (t, *J* = 1.7 Hz, 4H), 7.56 (ddd, *J* = 8.3, 4.8, 0.8 Hz, 4H), 7.19 (dd, *J* = 1.9, 1.3 Hz, 4H). ^13^C­{^1^H} (101 MHz, CD_3_CN) δ 151.24, 144.28, 139.26, 133.42, 130.80, 125.40, 121.77.

#### [Pt­(**L2**)_4_]­(BF_4_)_2_

A solution of *cis*-PtCl_2_(DMSO)_2_ in DMSO-*d*_6_ (80 mM, 2 mL, 160 μmol) was mixed with a solution of Ag­(BF_4_) in dry acetonitrile (160 mM, 2 mL). After 30 min, the precipitate was removed using a syringe filter to yield a 40 mM solution of [Pt­(CH_3_CN)_4_]­(BF_4_)_2_ in CH_3_CN:DMSO-*d*_6_ (1:1). Ligand **L2** (20.1 mg, 138 μmol, 4 equiv), acetonitrile (861 μL), and [Pt­(CH_3_CN)_4_]­(BF_4_)_2_ (40 mM, 861 μL, 34.4 μmol, 1 equiv) were combined and the mixture was heated at 80 °C overnight. The mixture was allowed to cool to RT and was filtered using a syringe filter (0.22 μm). The product was precipitated with toluene (13 mL) and washed with toluene (14 mL) using a centrifuge. The product was dried in air overnight and then dried under reduced pressure at RT for 1 h. [Pt­(**L2**)_4_]­(BF_4_)_2_ was obtained as a white powder (30.0 mg, 31.5 μmol, 91%). ^1^H (400 MHz, CD_3_CN) δ 9.25 (s, 4H), 8.98 (s, 8H), 8.39 (t, *J* = 1.4 Hz, 4H), 7.68 (t, *J* = 1.7 Hz, 4H), 7.26 (dd, *J* = 1.9, 1.3 Hz, 4H). ^13^C­{^1^H} (101 MHz, CD_3_CN) δ 159.43, 151.67, 139.39, 132.20, 131.10, 121.72.

#### [Pt­(**L3**)_4_]­(BF_4_)_2_

A solution of *cis*-PtCl_2_(DMSO)_2_ in DMSO-*d*_6_ (80 mM, 2 mL, 160 μmol) was mixed with a solution of Ag­(BF_4_) in dry acetonitrile (160 mM, 2 mL). After 30 min, the precipitate was removed using a syringe filter to yield a 40 mM solution of [Pt­(CH_3_CN)_4_]­(BF_4_)_2_ in CH_3_CN:DMSO-*d*_6_ (1:1). Ligand **L3** (20.1 mg, 138 μmol, 4 equiv), acetonitrile (861 μL), and [Pt­(CH_3_CN)_4_]­(BF_4_)_2_ (40 mM, 861 μL, 34.4 μmol, 1 equiv) were combined and the mixture was heated at 80 °C overnight. The mixture was allowed to cool to RT and was filtered using a syringe filter (0.22 μm). The product was precipitated with toluene (13 mL) and washed with toluene (14 mL) using a centrifuge. The product was dried in air overnight and then dried under reduced pressure at RT for 1 h. [Pt­(**L3**)_4_]­(BF_4_)_2_ was obtained as a white powder (25.7 mg, 27.0 μmol, 78%). ^1^H (400 MHz, CD_3_CN) δ 9.29 (s, 4H), 8.85 (s, 4H), 8.82 (dd, *J* = 2.8, 0.8 Hz, 4H), 8.77 (dd, *J* = 4.8, 1.4 Hz, 4H), 7.98 (ddd, *J* = 8.3, 2.7, 1.4 Hz, 4H), 7.62 (ddd, *J* = 8.3, 4.8, 0.8 Hz, 4H). ^13^C­{^1^H} (101 MHz, CD_3_CN) δ 152.28, 145.59, 144.80, 143.69, 131.71, 130.63, 125.61.

#### [Pt_2_Pd_4_(**L1**)_8_Cl_8_]­(BF_4_)_4_

[Pt­(**L1**)_4_]­(BF_4_)_2_ (1.0 eq., 0.8 μmol, 200 μL of a 4 mM stock solution in CD_3_NO_2_), 320 μL CD_3_NO_2_, and PdCl_2_(CH_3_CN)_2_ (2.0 eq., 1.6 μmol, 80 μL of a 20 mM stock solution in CD_3_NO_2_) were added to an NMR tube and heated at 80 °C for 20 h to give a 0.66 mM solution of cage [Pt_2_Pd_4_(**L1**)_8_Cl_8_]­(BF_4_)_4_. ^1^H (600 MHz, CD_3_NO_2_) δ 9.12 (s, 8H), 8.94 (d, *J* = 5.6 Hz, 8H), 8.80 (s, 8H), 8.16 (dd, *J* = 8.2, 2.0 Hz, 8H), 7.73 (dd, *J* = 8.5, 5.6 Hz, 8H), 7.66 (t, *J* = 1.8 Hz, 8H), 7.36 (s, 8H). ^13^C­{^1^H} (151 MHz, CD_3_NO_2_) δ 154.87, 147.40, 139.85, 135.21, 134.55, 131.61, 127.65, 122.34, 119.10.

#### [Pt_2_Pd_4_(**L2**)_8_Cl_8_]­(BF_4_)_4_

[Pt­(**L2**)_4_]­(BF_4_)_2_ (1.0 eq., 0.8 μmol, 200 μL of a 4 mM stock solution in CD_3_NO_2_), 320 μL CD_3_NO_2_, and PdCl_2_(CH_3_CN)_2_ (2.0 eq., 1.6 μmol, 80 μL of a 20 mM stock solution in CD_3_NO_2_) were added to an NMR tube and heated at 80 °C for 20 h to give a 0.66 mM solution of cage [Pt_2_Pd_4_(**L1**)_8_Cl_8_]­(BF_4_)_4_. ^1^H (600 MHz, CD_3_CN) δ 9.55 (s, 8H), 9.41 (d, *J* = 2.9 Hz, 8H), 9.14 (d, *J* = 2.9 Hz, 8H), 8.84 (s, 8H), 7.75 (t, *J* = 1.8 Hz, 8H), 7.47–7.42 (m, 8H). ^13^C­{^1^H} (151 MHz, CD_3_CN) δ 161.52, 154.54, 140.19, 133.02, 131.93, 126.23, 122.22, 119.09.

#### [Pt_2_Pd_4_(**L3**)_8_Cl_8_]­(BF_4_)_4_

[Pt­(**L3**)_4_]­(BF_4_)_2_ (1.0 eq., 0.8 μmol, 200 μL of a 4 mM stock solution in CD_3_NO_2_), 320 μL CD_3_NO_2_, and PdCl_2_(CH_3_CN)_2_ (2.0 eq., 1.6 μmol, 80 μL of a 20 mM stock solution in CD_3_NO_2_) were added to an NMR tube and equilibrated at RT for 1 d to yield cage [Pt_2_Pd_4_(**L3**)_8_Cl_8_]­(BF_4_)_4_. ^1^H (400 MHz, CD_3_NO_2_) δ 9.54 (s, 8H), 9.17 (d, *J* = 2.4 Hz, 8H), 9.03–8.99 (m, 8H), 8.85 (s, 8H), 8.28 (d, *J* = 9.3 Hz, 8H), 7.81 (dd, *J* = 8.6, 5.7 Hz, 8H).

### X-ray Crystallography

Data were collected using a XtaLAB Synergy R, DW system, HyPix-Arc 150 diffractometer operating at 99.98(16) K, 140.00(10) K, or 229.99(10) K. Data were measured using ω scans with Cu Kα or Mo Kα radiation. Details of the crystallographic refinements are provided in the SI. The crystallographic data have been deposited with the Cambridge Crystallographic Data Centre under accession codes 2541152–2541160.

## Supplementary Material



## Notes

Synthetic procedures, analytical data; crystallographic data (PDF)
