# Supplementary material for: Orientational Self-Sorting in Octahedral Coordination Cages Based on Small, Heteroditopic N‑Donor Ligands
Source: Inorg Chem. 2026 Jul 27;65(31):18074–81. doi: 10.1021/acs.inorgchem.6c02256 (PMC13463681; doi:10.1021/acs.inorgchem.6c02256)
Supplement: Supplementary file 1 [file ic6c02256_si_001.pdf]

Supporting Information

Orientational Self-Sorting in Octahedral Coordination Cages Based on Small, Heteroditopic N-Donor Ligands

Jean de Montmollin,[a] Cédric Przybylski,[b] Jean-Yves Salpin,[b] Daniel Ortiz,[a] Pol Gorrea-Acín,[a] Atena B. Solea,[a] Farzaneh Fadaei-Tirani,[a] and Kay Severin*[a]

[a] *Institute of Chemical Sciences and Engineering, École Polytechnique Fédérale de Lausanne (EPFL), 1015 Lausanne, Switzerland. Email : kay.severin@epfl.ch*

[b] *Université Paris-Saclay, Univ. Evry, CY Cergy Paris Université, CNRS, LAMBE, 91025, Evry-Courcouronnes, France.*

Table of Contents

1 General 3
2 Ligand and Homoleptic Cage Syntheses 4
2.1 Synthesis of L1 4
2.2 Synthesis of L2 5
2.3 Synthesis of $[Pd_6(L1)_{12}](BF_4)_{12}$ 6
2.4 Synthesis of $[Pd_6(L2)_{12}](BF_4)_{12}$ 8
2.5 Synthesis of $[Pd_6(L3)_{12}](BF_4)_{12}$ 10
2.6 Variable temperature NMR with $[Pd_6(L1–3)_{12}](BF_4)_{12}$ 12
2.7 $[Pd(L1–3)_4](BF_4)_2$ complexes and $[Pd_6(L1–3)_{12}](BF_4)_{12}$ cages comparison 13
3 Complex and Heterometallic Cage Syntheses 16
3.1 Synthesis of $[Pt(L1)_4](BF_4)_2$ 16
3.2 Synthesis of $[Pt(L2)_4](BF_4)_2$ 19
3.3 Synthesis of $[Pt(L3)_4](BF_4)_2$ 21
3.4 Synthesis of $[Pt_2Pd_4(L1)_8Cl_8](BF_4)_4$ 23
3.5 Synthesis of $[Pt_2Pd_4(L2)_8Cl_8](BF_4)_4$ 26
3.6 Synthesis of $[Pt_2Pd_4(L3)_8Cl_8](BF_4)_4$ 29
4 Cyclic Ion Mobility-Mass spectrometry (cIM-MS) 32
5 Crystallographic analyses 40
6 References 54

1 General

All chemicals were obtained from commercial sources and used without further purification unless stated otherwise. The ligands **L1**,[S1] **L2**,[S1] and **L3**[S2] (Figure S1) were prepared according to (or in analogy to) procedures described in the literature.

NMR spectra were measured on a Bruker Avance III HD spectrometer (^{1}H: 400 MHz) equipped with a BBFO-Plus$_z$ 5 mm probe, a Bruker Avance III HD spectrometer (^{1}H: 600 MHz) equipped with a CPPBBO$_z$ 5 mm probe or a Bruker Avance II HD spectrometer (^{1}H: 800 MHz) equipped with a CPTCI$_{XYZ}$ 5 mm probe.

High-resolution mass spectrometry experiments were carried out using a hybrid ion trap-Orbitrap Fourier transform mass spectrometer, Orbitrap Elite (Thermo Scientific) equipped with a TriVersa Nanomate (Advion) nano-electrospray ionization source.

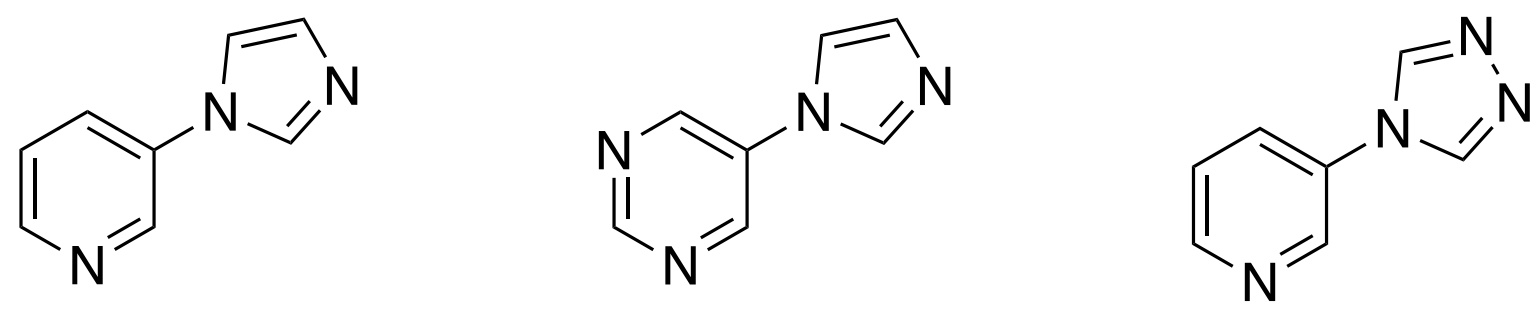

L1 **L2** **L3**

Figure S1. Structures of the ligands **L1–L3**.

2 Ligand and Homoleptic Cage Syntheses

2.1 Synthesis of **L1**

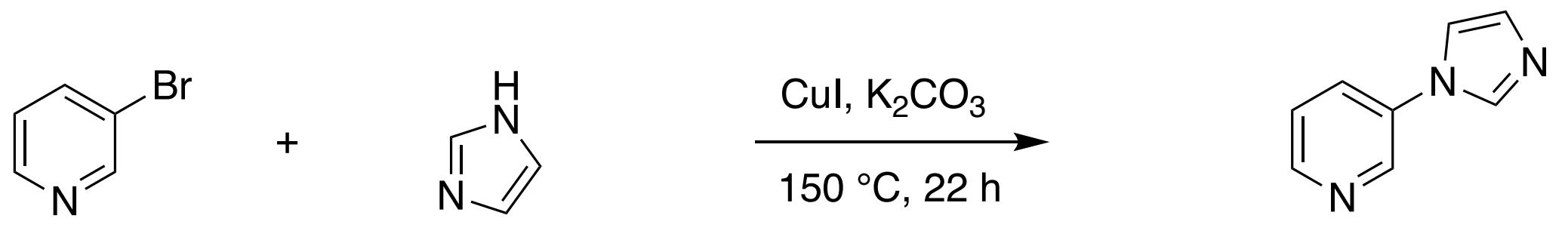

3-Bromopyridine (1.0 eq., 1 mL, 10.3 mmol), imidazole (2.0 eq., 1.40 g, 20.6 mmol), potassium carbonate (1.5 eq., 2.13 g, 15.4 mmol), and copper(I) iodide (0.1 eq., 196 mg, 1.03 mmol) were added to a 25 mL Schlenk tube. The flask was evacuated and refilled with nitrogen 3 times and was heated without solvent to 150 °C overnight. The mixture was allowed to cool down to RT and transferred with 50 mL water and 100 mL DCM to a separatory funnel. The organic phase was washed with water (4 × 100 mL) and brine (1 × 100 mL). The organic phase was dried over $MgSO_4$ and the solvent was evaporated at 700 mbar/50 °C (caution: the product is volatile). The remaining DCM was allowed to evaporate at open air overnight to yield **L1** in the form of a yellow oil (566 mg, 3.9 mmol, 38%). ^{1}H (400 MHz, CD_3CN) δ 8.79 (dd, *J* = 2.8, 0.8 Hz, 1H), 8.57 (dd, *J* = 4.8, 1.5 Hz, 1H), 7.97 (s, 1H), 7.89 (ddd, *J* = 8.2, 2.7, 1.4 Hz, 1H), 7.55 – 7.43 (m, 2H), 7.21 – 7.09 (m, 1H). ^{13}C{^{1}H} (101 MHz, CD_3CN) δ 149.23, 143.42, 136.77, 134.84, 131.20, 129.56, 125.22, 119.20. HRMS (nanochip-ESI/LTQ-Orbitrap) *m/z*: [M + H]$^+$ Calculated for $C_8H_8N_3^+$ 146.0713; Found 146.0711.

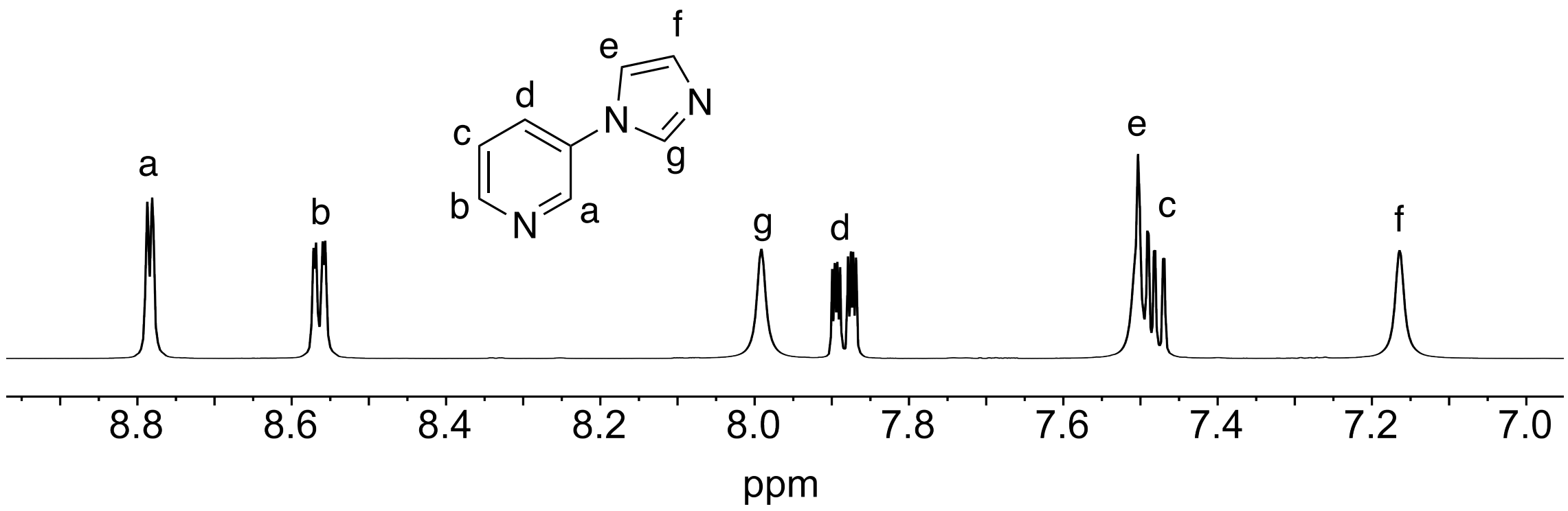

Figure S2. ^{1}H NMR spectrum (400 MHz, CD_3CN) of **L1**.

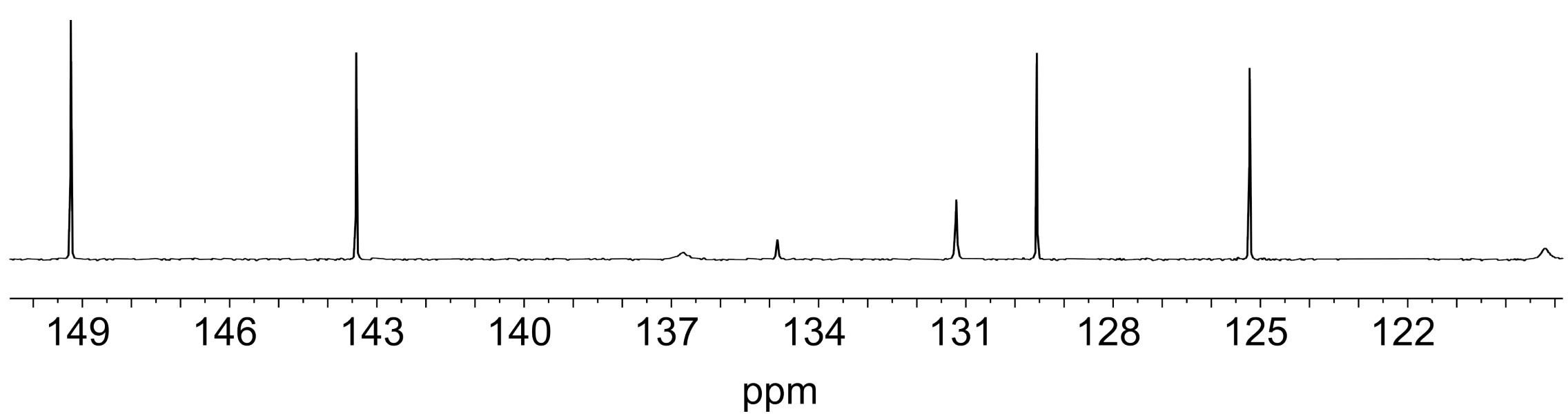

Figure S3. ^{13}C{^{1}H} NMR spectrum (101 MHz, CD_3CN) of **L1**.

2.2 Synthesis of **L2**

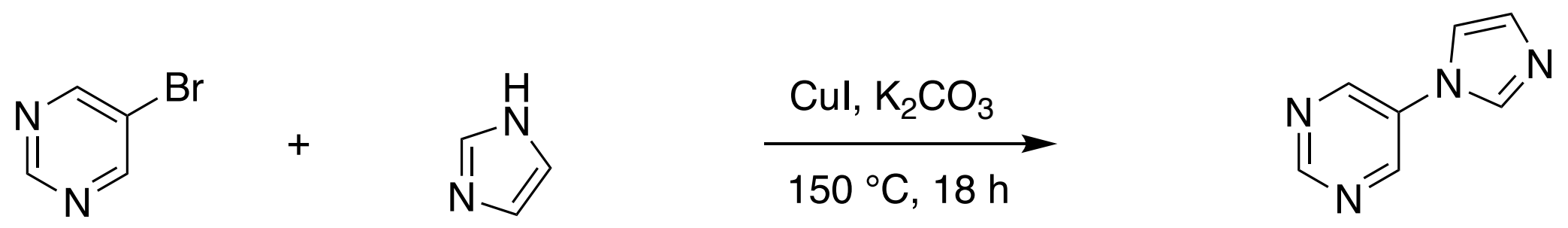

L2

5-Bromopyrimidine (1.0 eq., 245 mg, 1.54 mmol), imidazole (10.0 eq., 1.08 g, 15.4 mmol), potassium carbonate (1.56 eq., 333 mg, 1.41 mmol), and copper(I) iodide (0.1 eq., 29.4 mg, 0.154 mmol) were added to a 25 mL Schlenk tube. The flask was evacuated and refilled with nitrogen 3 times and was heated without solvent to 150 °C overnight. The mixture was allowed to cool down to RT and transferred with 50 mL water to a separatory funnel and extracted with DCM (2 × 100 mL). The organic phase was washed with water (1 × 100 mL) and brine (1 × 100 mL). The organic phase was dried over $MgSO_4$ and the solvent was evaporated under reduced pressure to yield **L2** as a white solid (200 mg, 1.37 mmol, 89%). ^{1}H (400 MHz, CD_3CN) δ 9.13 (s, 1H), 8.96 (s, 2H), 8.01 (t, *J* = 1.1 Hz, 1H), 7.53 (t, *J* = 1.4 Hz, 1H), 7.20 (t, *J* = 1.1 Hz, 1H). ^{13}C{^{1}H} (101 MHz, CD_3CN) δ 157.71, 150.37, 136.64, 133.52, 131.78, 118.85. HRMS (nanochip-ESI/LTQ-Orbitrap) *m/z*: $[M + H]^+$ Calculated for $C_7H_6N_4^+$ 147.06684; Found 147.06686.

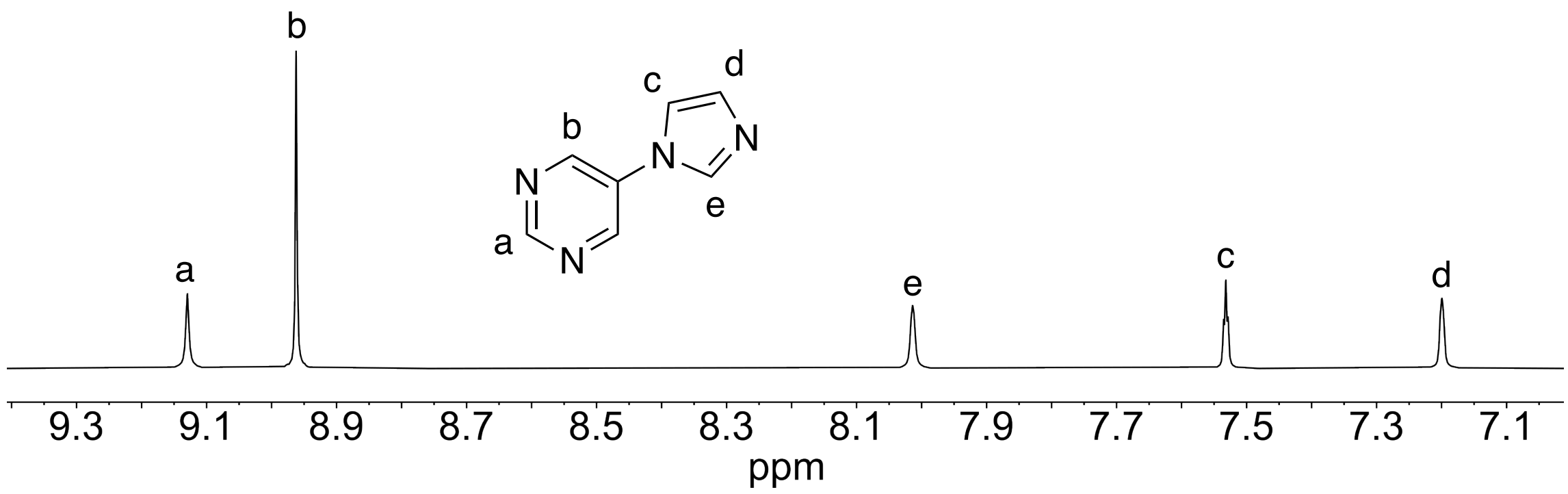

Figure S4. ^{1}H NMR spectrum (400 MHz, CD_3CN) of **L2**.

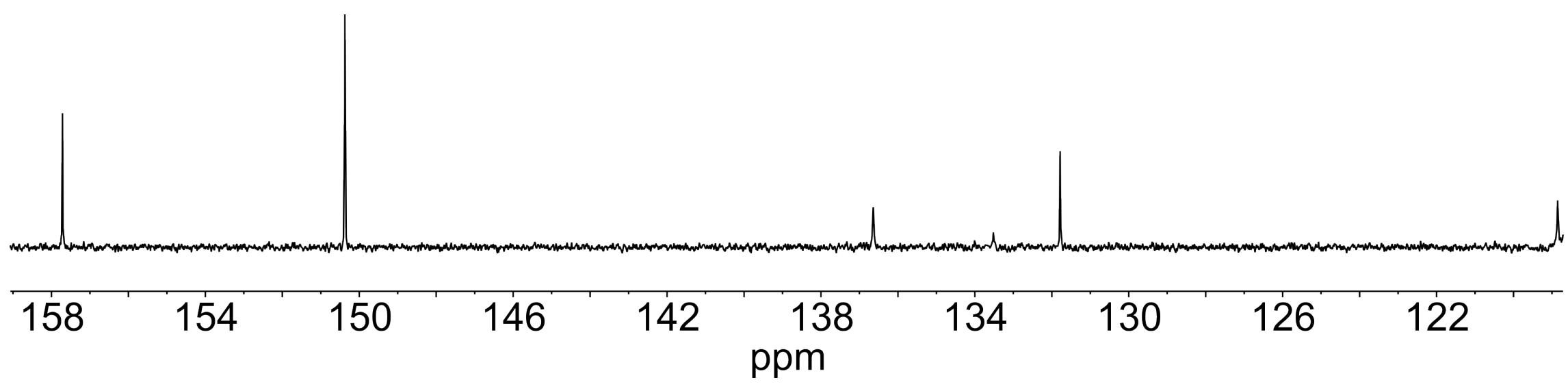

Figure S5. ^{13}C{^{1}H} NMR spectrum (101 MHz, CD_3CN) of **L2**.

2.3 Synthesis of $[Pd_6(\mathbf{L1})_{12}](BF_4)_{12}$

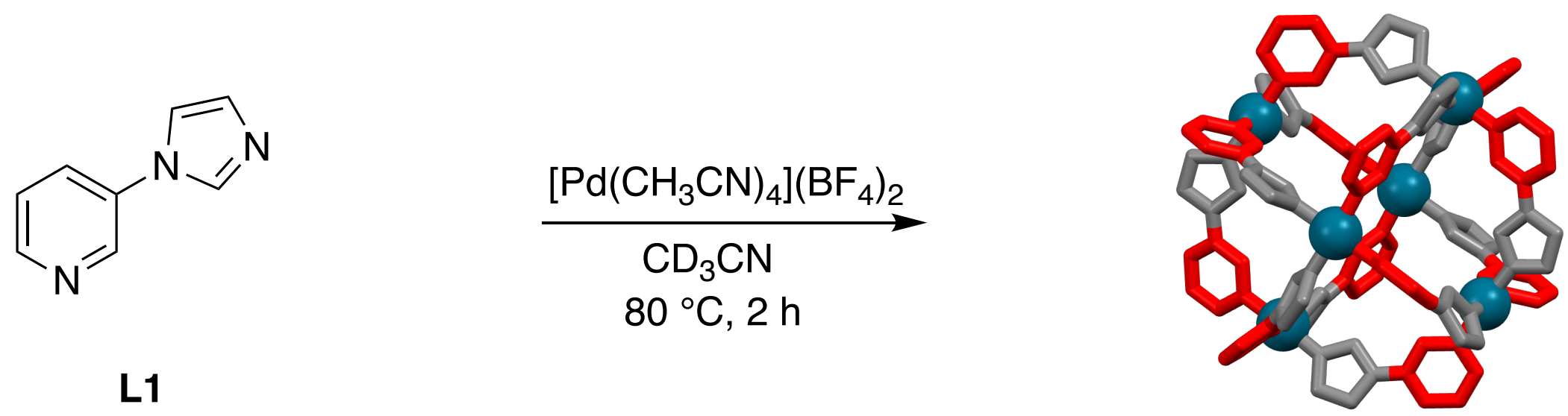

L1

$[Pd_6(\mathbf{L1})_{12}](BF_4)_{12}$

L1 (2.0 eq., 2.4 µmol, 60 µL of a 40 mM stock solution in CD_3CN), 500 µL CD_3CN, and $[Pd(CH_3CN)_4](BF_4)_2$ (1.0 eq., 1.2 µmol, 40 µL of a 30 mM stock solution in CD_3CN) were added to an NMR tube and heated at 80 °C for 20 h to give a solution of cage $[Pd_6(\mathbf{L1})_{12}](BF_4)_{12}$. $[Pd_6(\mathbf{L1})_{12}](OTf)_{12}$ was obtained similarly by heating a mixture of $Pd(OTf)_2$ and ligand **L1** at 86 °C overnight. A stock solution of $Pd(OTf)_2$ was obtained by a reaction of $PdCl_2(CH_3CN)_2$ with AgOTf in CD_3CN, followed by removal of AgCl with a syringe filter.

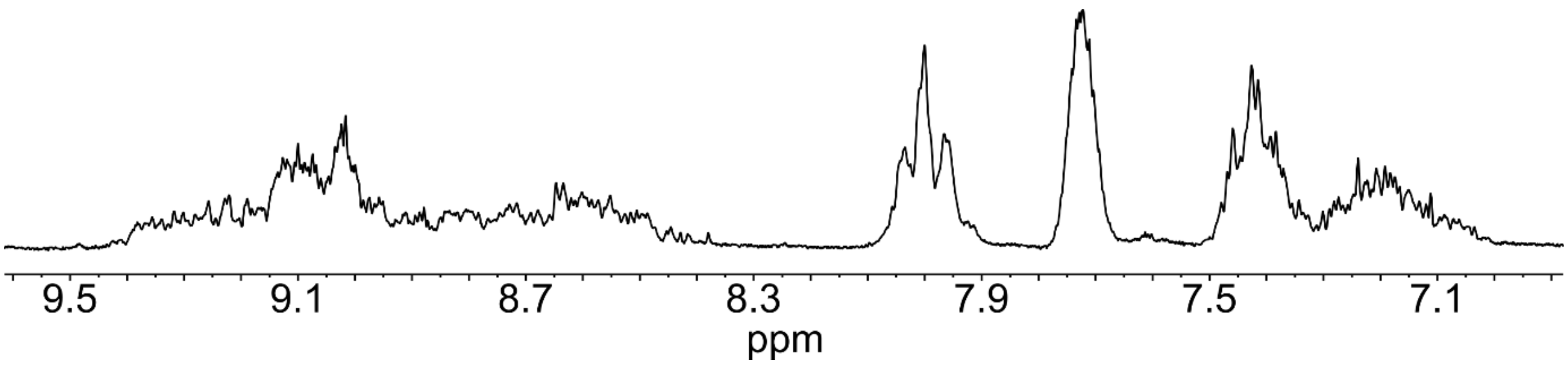

Figure S6. ^{1}H NMR spectrum (800 MHz, CD_3CN) of a solution containing **L1** (2 equiv.) and $[Pd(CH_3CN)_4](BF_4)_2$ (1 equiv.) after thermal equilibration.

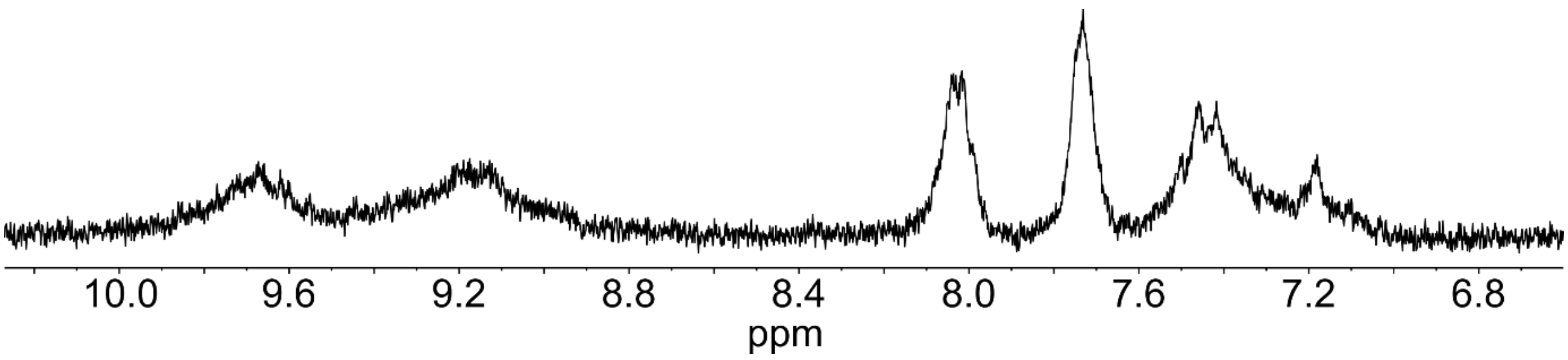

Figure S7. ^{1}H NMR spectrum (400 MHz, CD_3CN) of a solution containing **L1** (2 equiv.) and $Pd(OTf)_2$ (1 equiv.) after thermal equilibration.

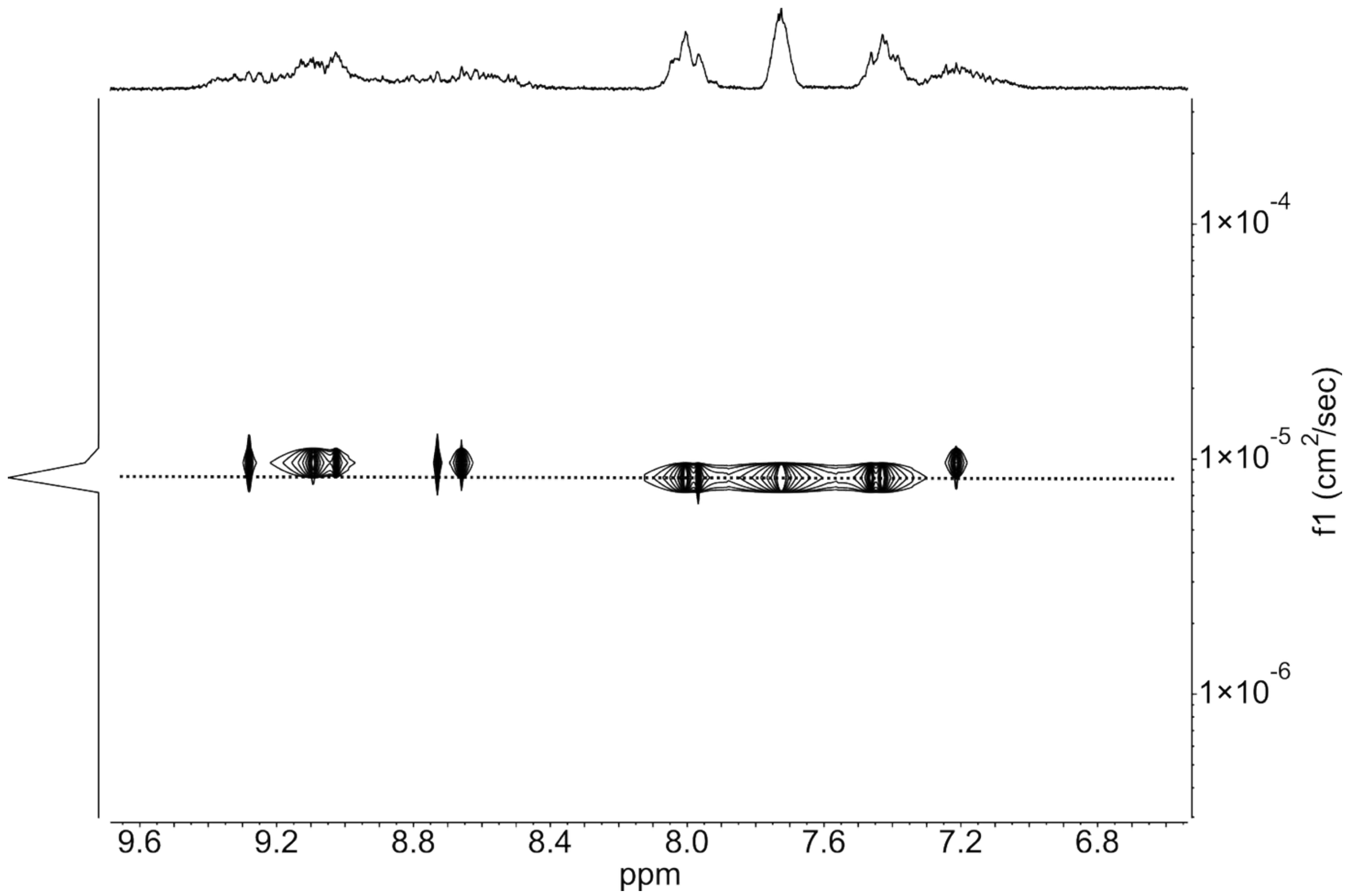

Figure S8. DOSY NMR spectrum (600 MHz, CD_3CN) of a solution containing **L1** (2 equiv.) and $[Pd(CH_3CN)_4](BF_4)_2$ (1 equiv.) after thermal equilibration (D = 8.39 x 10^{-6} cm^2 s^{-1}).

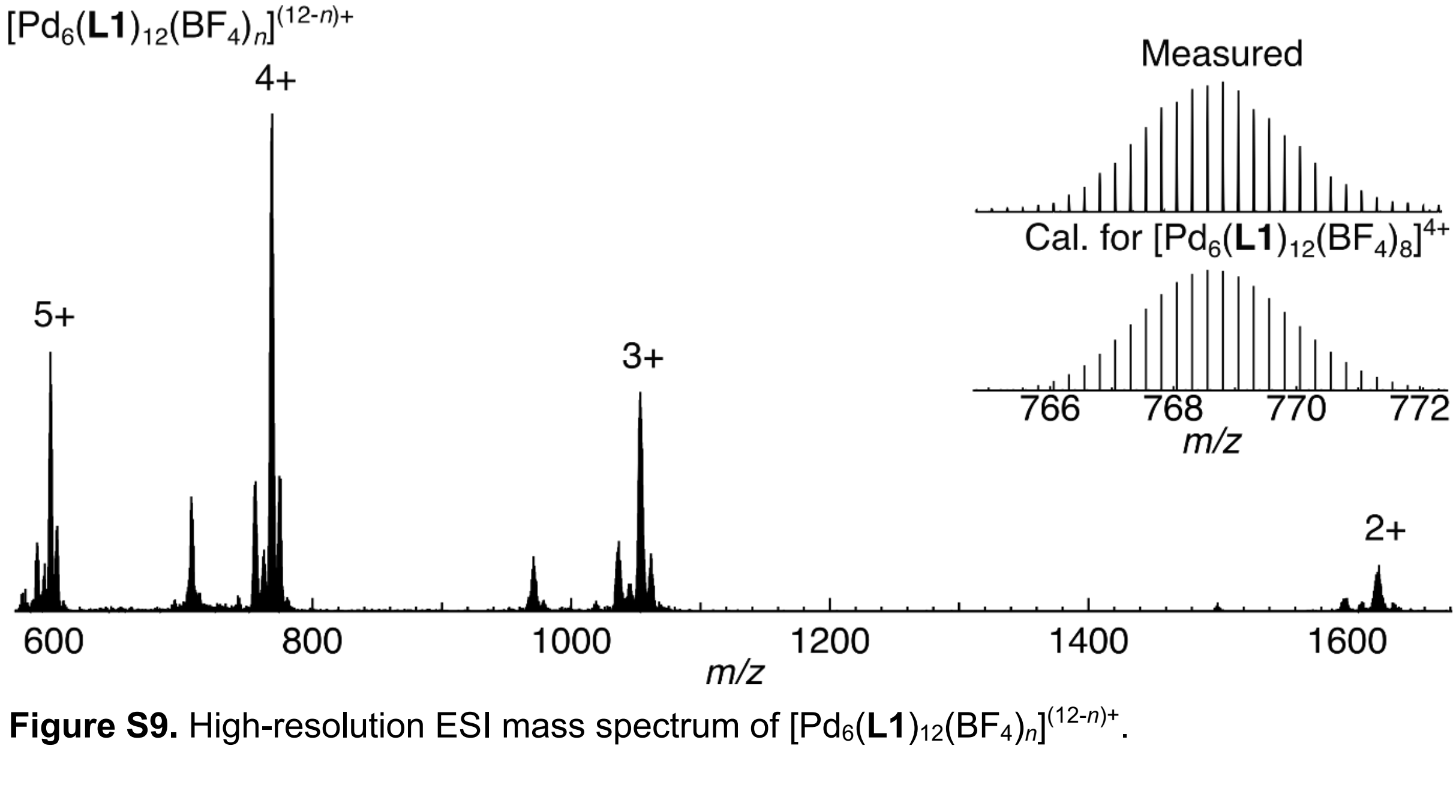

Figure S9. High-resolution ESI mass spectrum of $[Pd_6(\mathbf{L1})_{12}(BF_4)_n]^{(12-n)+}$.

2.4 Synthesis of $[Pd_6(\mathbf{L2})_{12}](BF_4)_{12}$

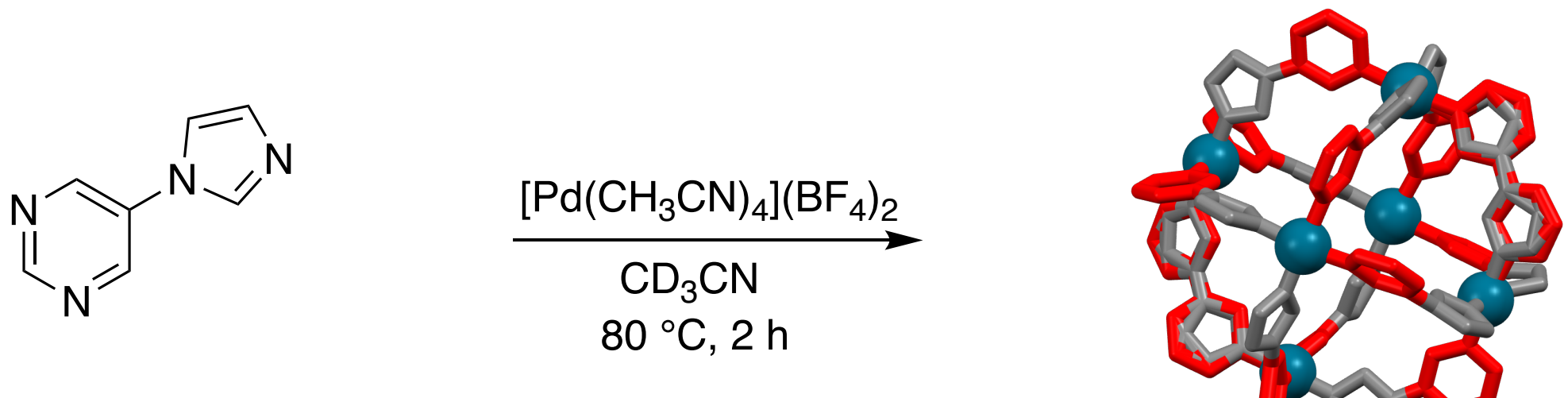

L2

$[Pd_6(\mathbf{L2})_{12}](BF_4)_{12}$ (+ side products)

L2 (2.0 eq., 2.4 µmol, 60 µL of a 40 mM stock solution in CD_3CN), 500 µL CD_3CN, and $[Pd(CH_3CN)_4](BF_4)_2$ (1.0 eq., 1.2 µmol, 40 µL of a 30 mM stock solution in CD_3CN) were added to an NMR tube and heated at 80 °C for 20 h to give a solution of cage $[Pd_6(\mathbf{L2})_{12}](BF_4)_{12}$.

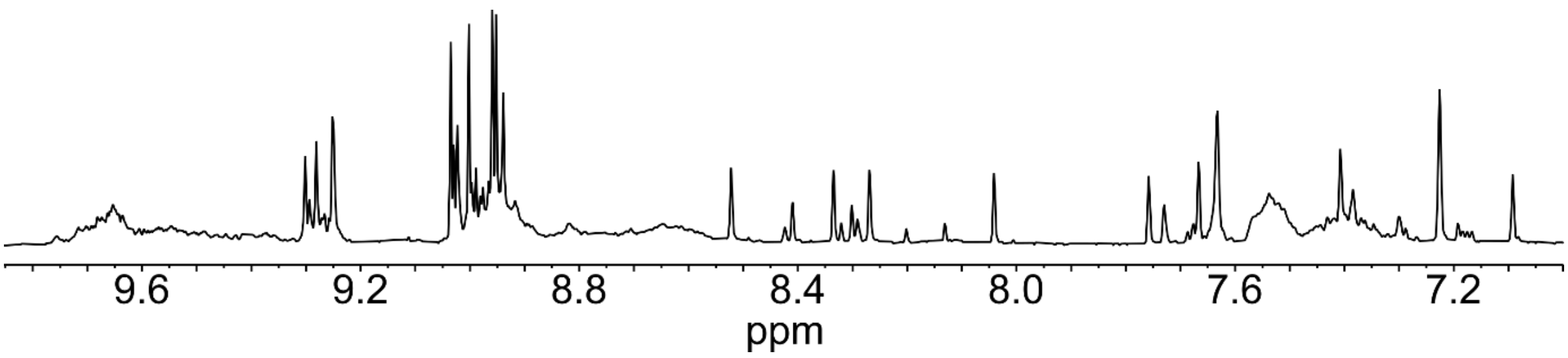

Figure S10. ^{1}H NMR spectrum (800 MHz, CD_3CN) of a solution containing **L2** (2 equiv.) and $[Pd(CH_3CN)_4](BF_4)_2$ (1 equiv.) after thermal equilibration.

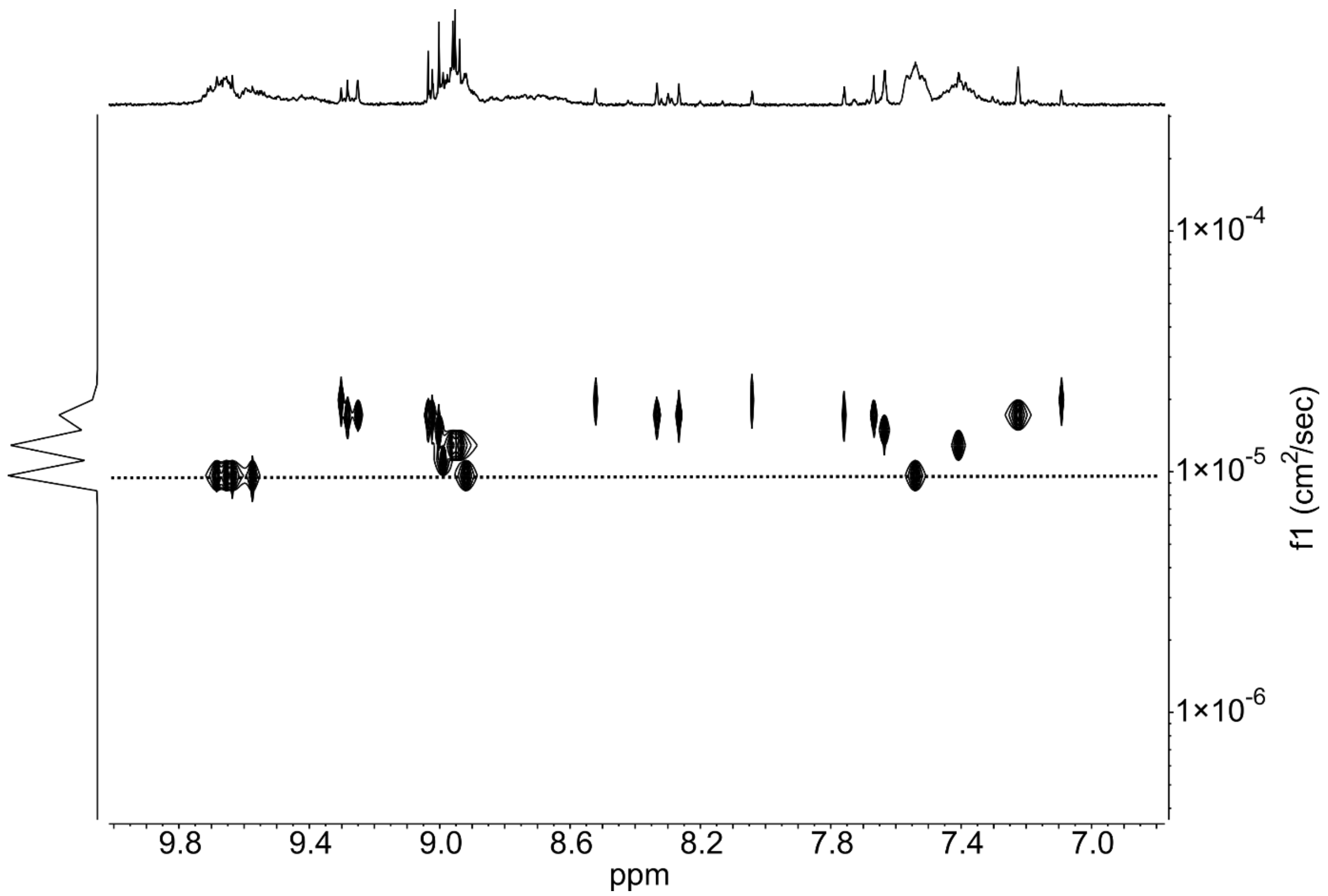

Figure S11. DOSY NMR spectrum (600 MHz, CD_3CN) of a solution containing **L2** (2 equiv.) and $[Pd(CH_3CN)_4](BF_4)_2$ (1 equiv.) after thermal equilibration. Dashed line: $[Pd_6(\mathbf{L2})_{12}](BF_4)_{12}$ (D = 9.65 x 10^{-6} cm^2 s^{-1}).

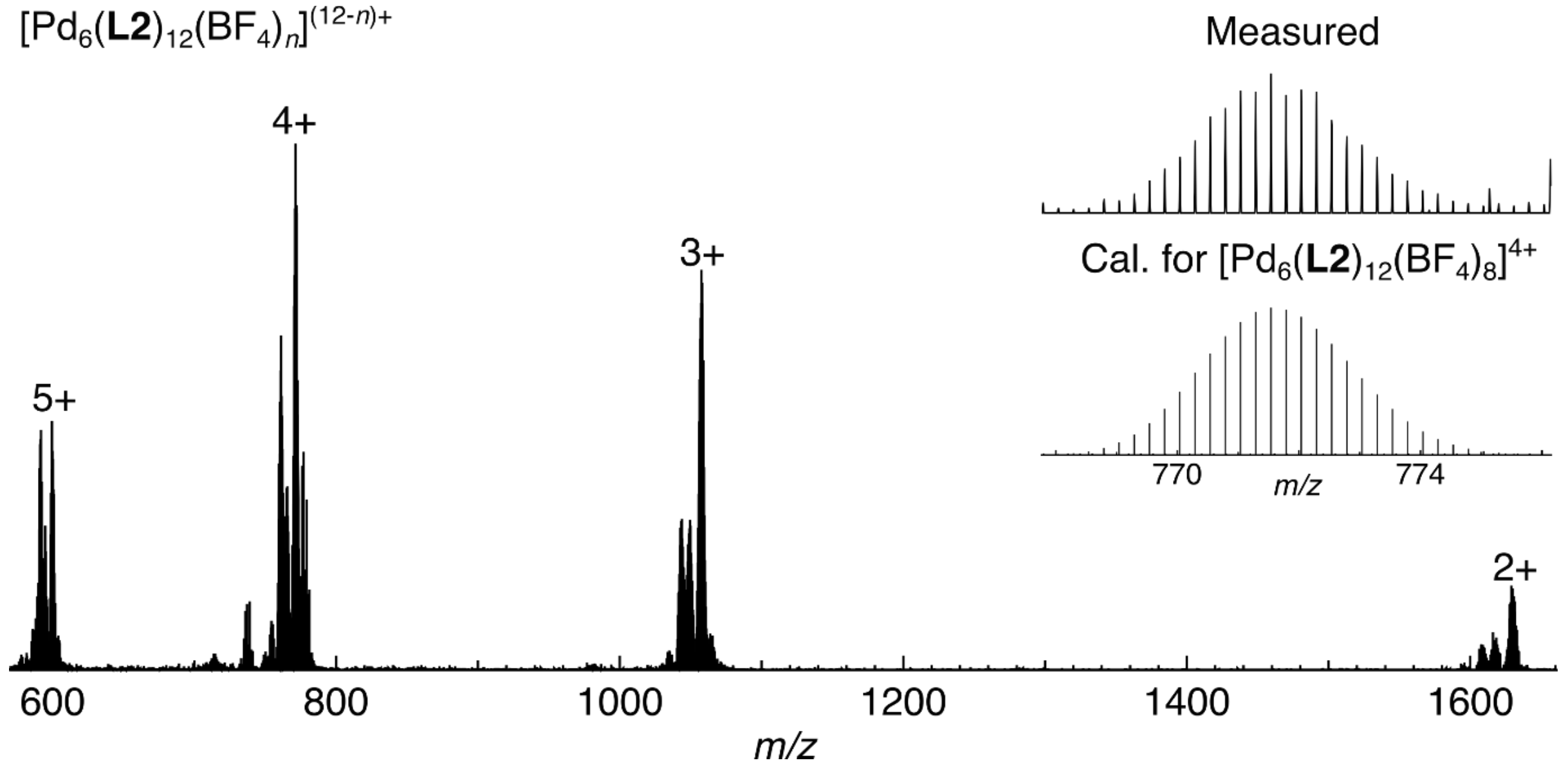

Figure S12. High-resolution ESI mass spectrum of $[Pd_6(\mathbf{L2})_{12}(BF_4)_n]^{(12-n)+}$.

2.5 Synthesis of $[Pd_6(\mathbf{L3})_{12}](BF_4)_{12}$

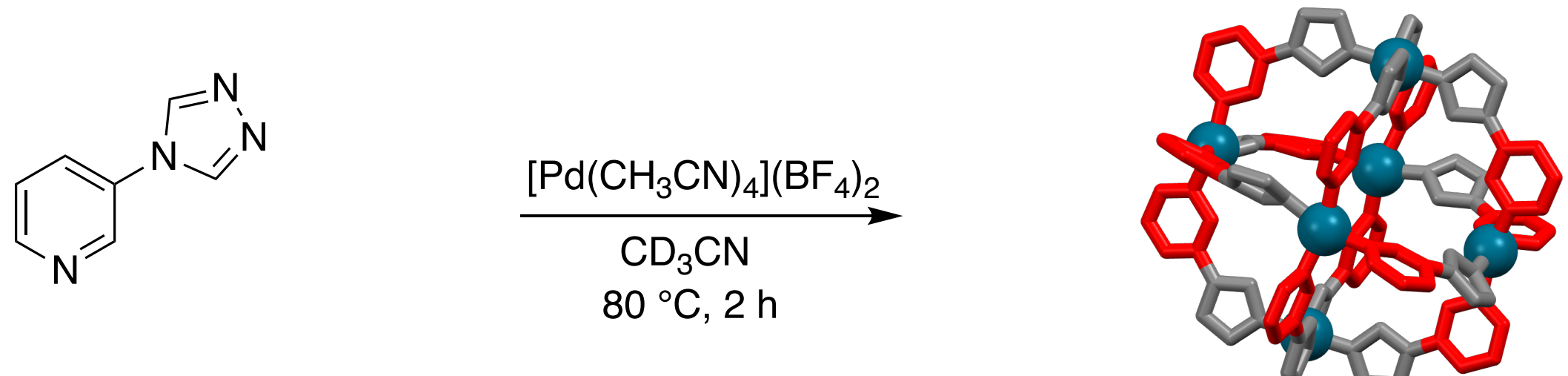

L3

$[Pd_6(\mathbf{L3})_{12}](BF_4)_{12}$ (+ side products)

L3 (2.0 eq., 2.4 μmol, 60 μL of a 40 mM stock solution in CD_3CN), 500 μL CD_3CN, and $[Pd(CH_3CN)_4](BF_4)_2$ (1.0 eq., 1.2 μmol, 40 μL of a 30 mM stock solution in CD_3CN) were added to an NMR tube and heated at 80 °C for 20 h to give a solution of cage $[Pd_6(\mathbf{L3})_{12}](BF_4)_{12}$.

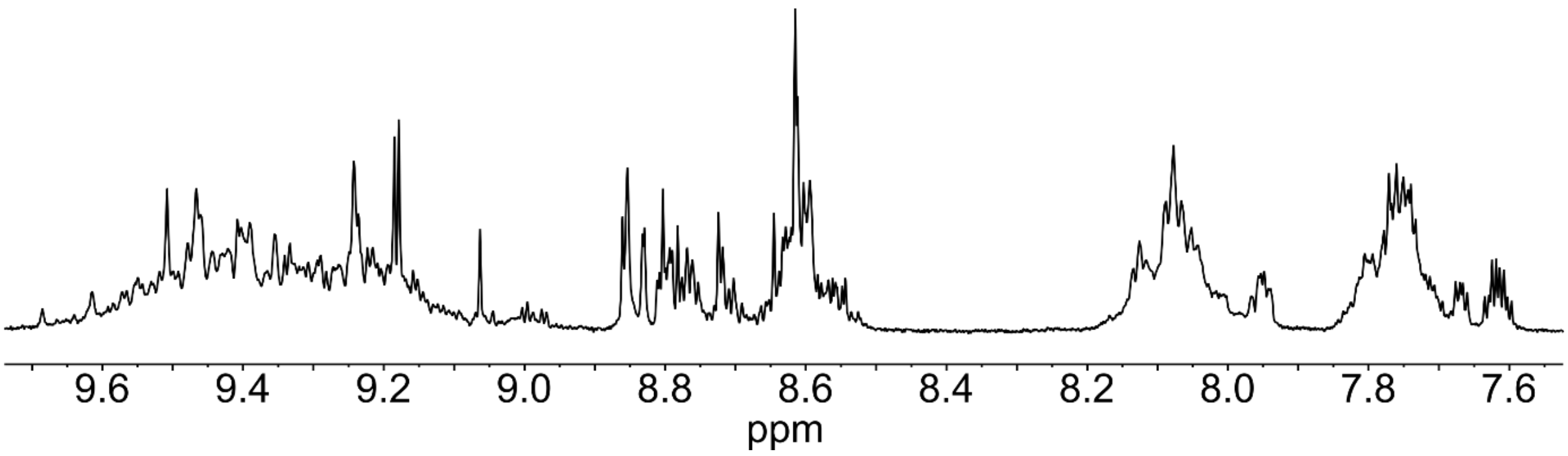

Figure S13. ^{1}H NMR spectrum (800 MHz, CD_3CN) of a solution containing **L3** (2 equiv.) and $[Pd(CH_3CN)_4](BF_4)_2$ (1 equiv.) after thermal equilibration.

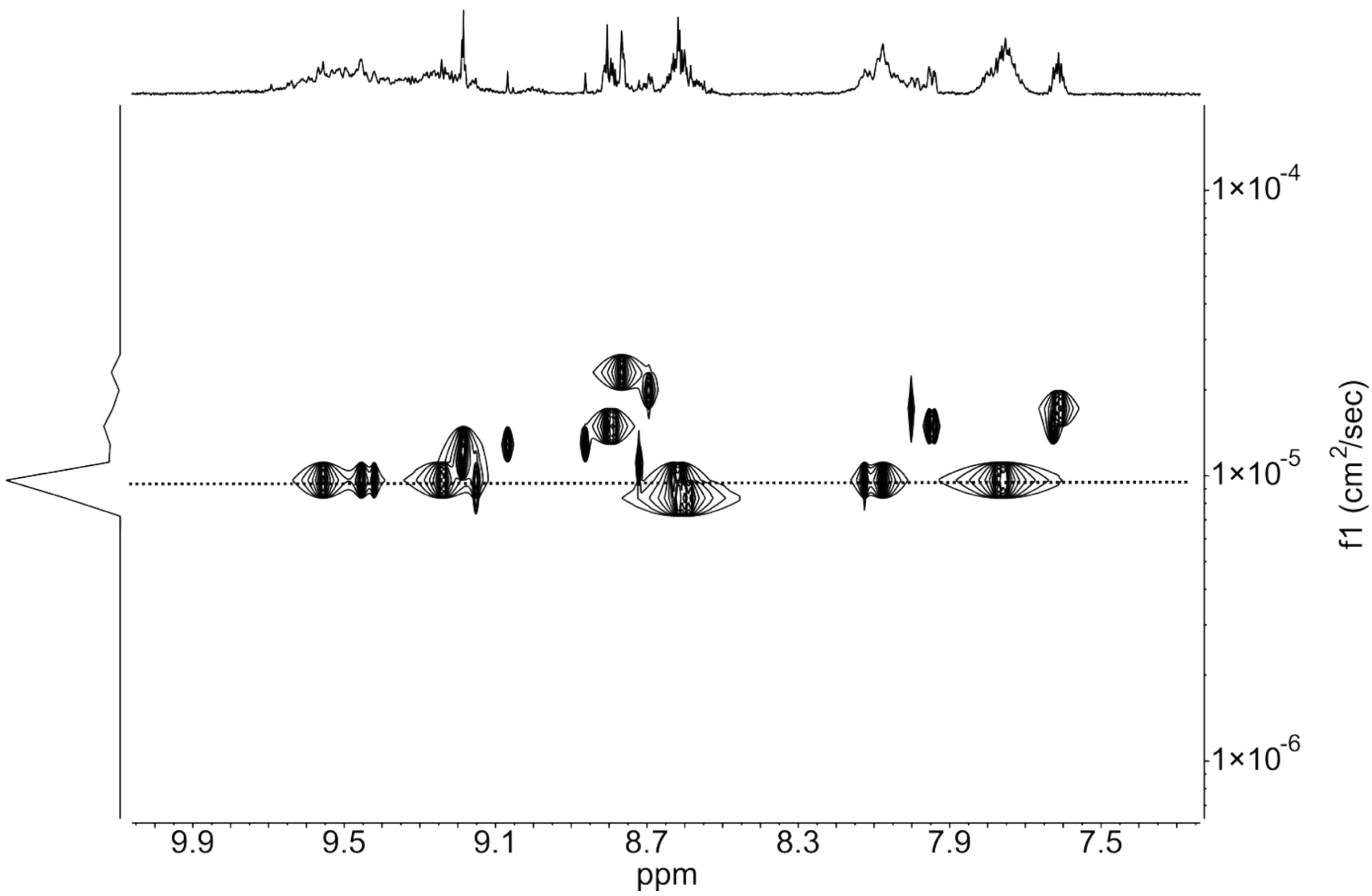

Figure S14. DOSY NMR spectrum (600 MHz, CD_3CN) of a solution containing **L3** (2 equiv.) and $[Pd(CH_3CN)_4](BF_4)_2$ (1 equiv.) after thermal equilibration. Dashed line: $[Pd_6(\mathbf{L3})_{12}](BF_4)_{12}$ (D = 9.66 x 10^{-6} cm^2 s^{-1}).

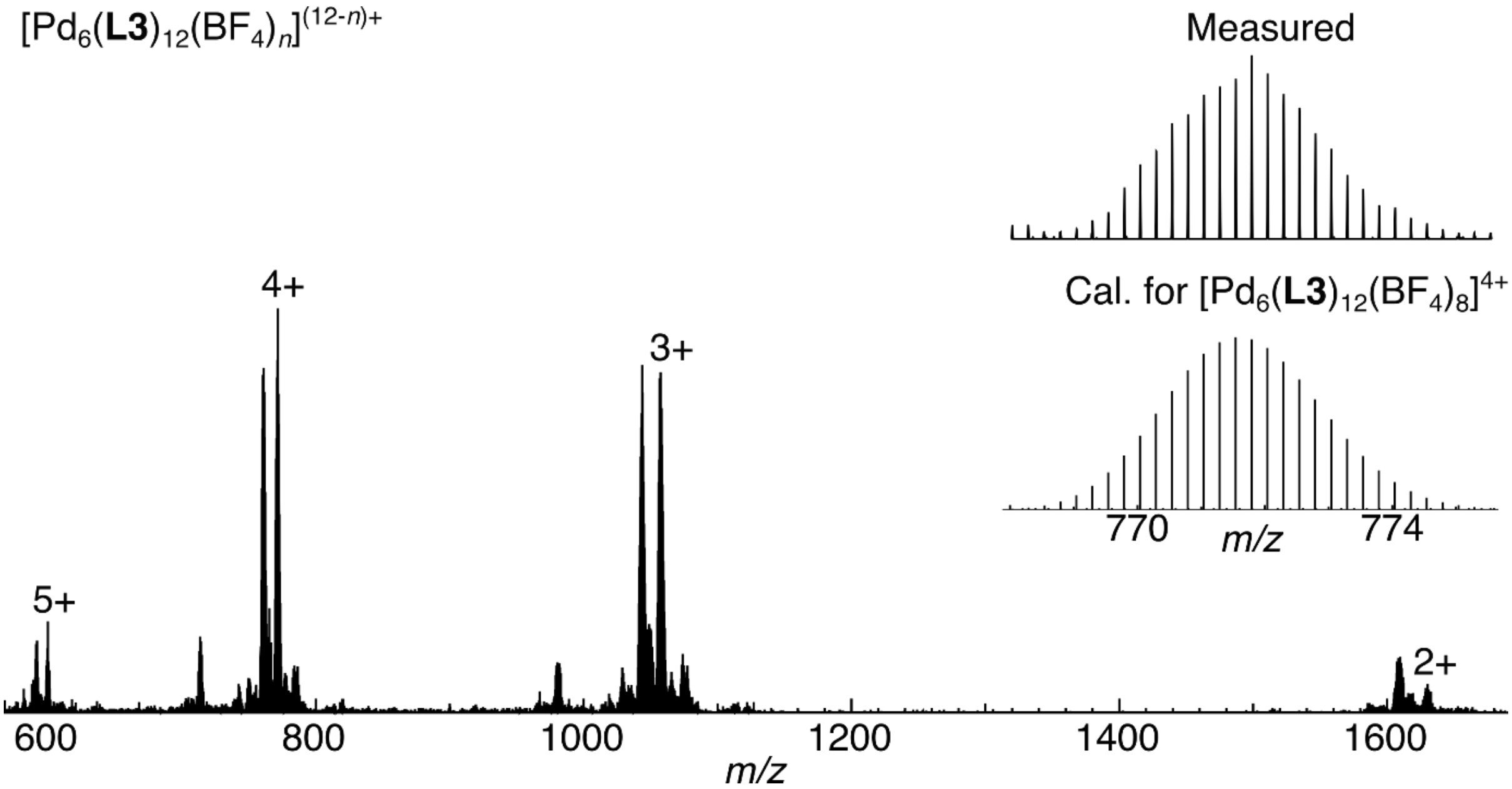

Figure S15. High-resolution ESI mass spectrum of $[Pd_6(\mathbf{L3})_{12}(BF_4)_n]^{(12-n)+}$.

2.6 Variable temperature NMR with $[Pd_6(\mathbf{L1–3})_{12}](BF_4)_{12}$

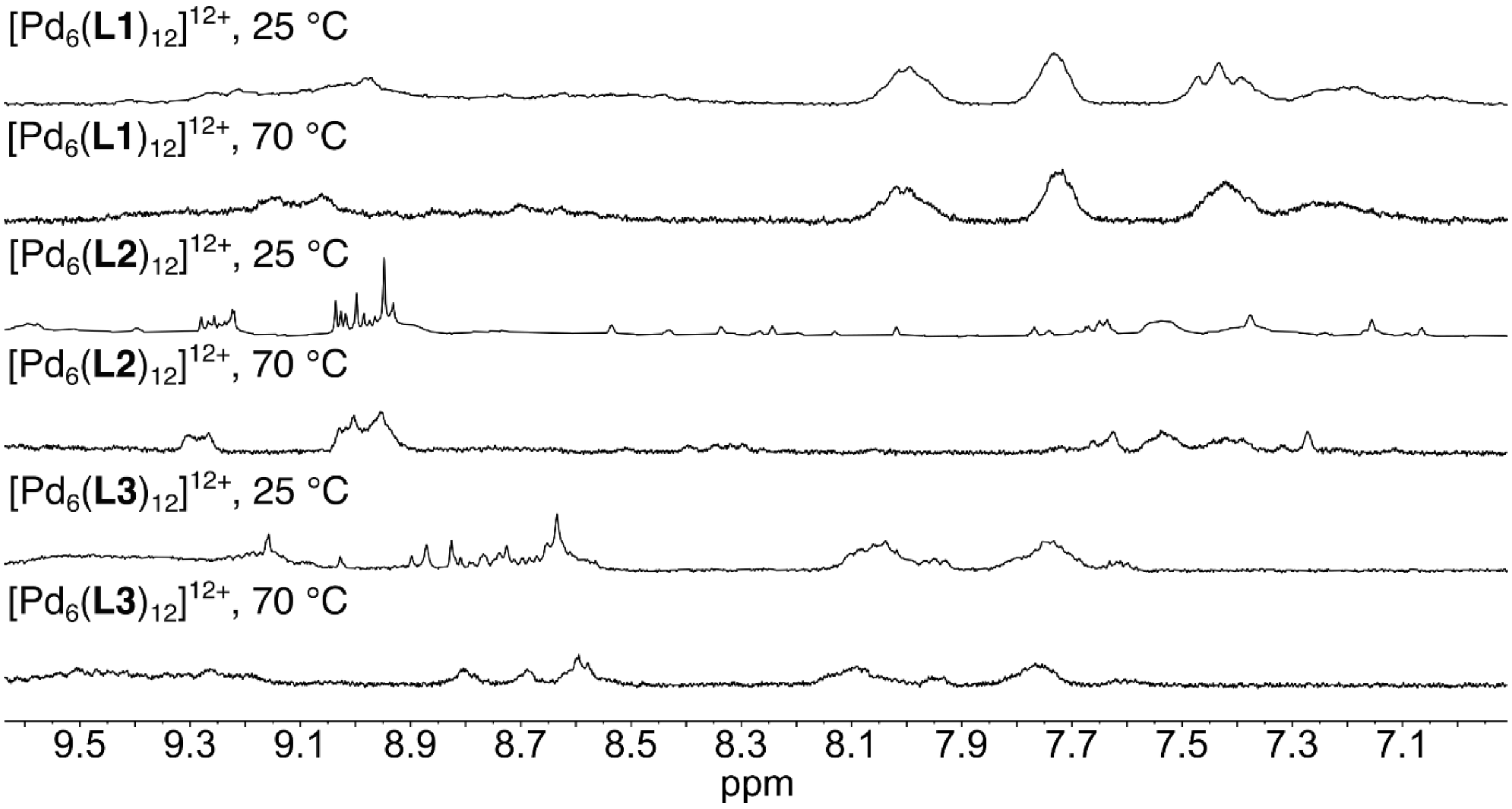

Figure S16. ^{1}H NMR spectrum (400 MHz, CD_3CN) of $[Pd_6(\mathbf{L1–3})_{12}](BF_4)_{12}$ at 25 °C and 70 °C.

2.7 [Pd(**L1–3**)$_4$](BF_4)$_2$ complexes and [Pd$_6$(**L1–3**)$_{12}$](BF_4)$_{12}$ cages comparison

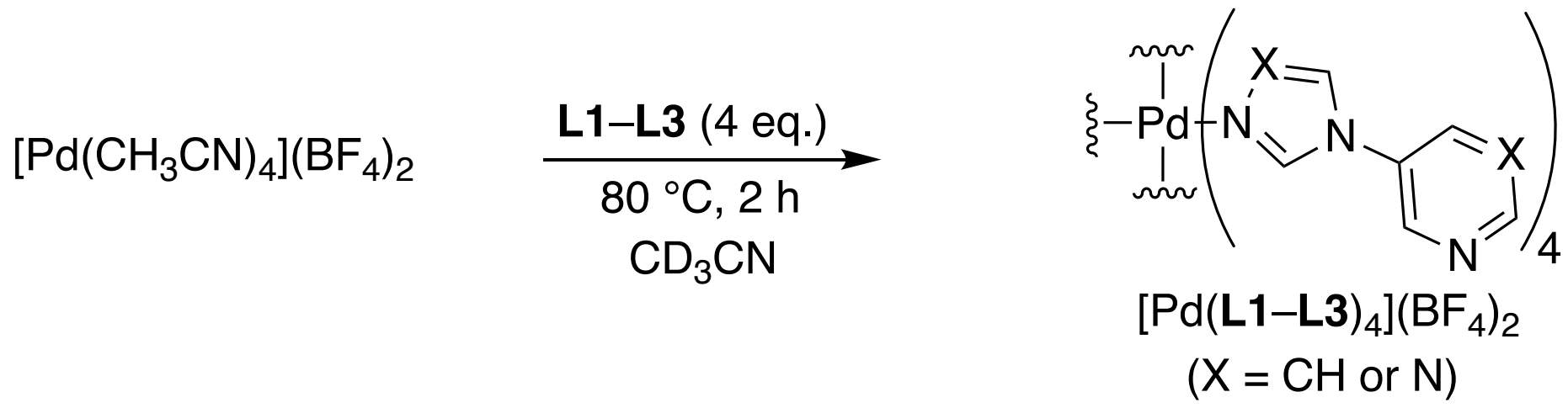

L1–3 (4.0 eq., 2.4 μmol, 60 μL of a 40 mM stock solution in CD_3CN), 500 μL CD_3CN, and [Pd(CH_3CN)$_4$](BF_4)$_2$ (1.0 eq., 0.6 μmol, 20 μL of a 30 mM stock solution in CD_3CN) were added to an NMR tube and heated at 80 °C for 2 h to give a solutions of complexes [Pd(**L1–3**)$_4$](BF_4)$_2$.

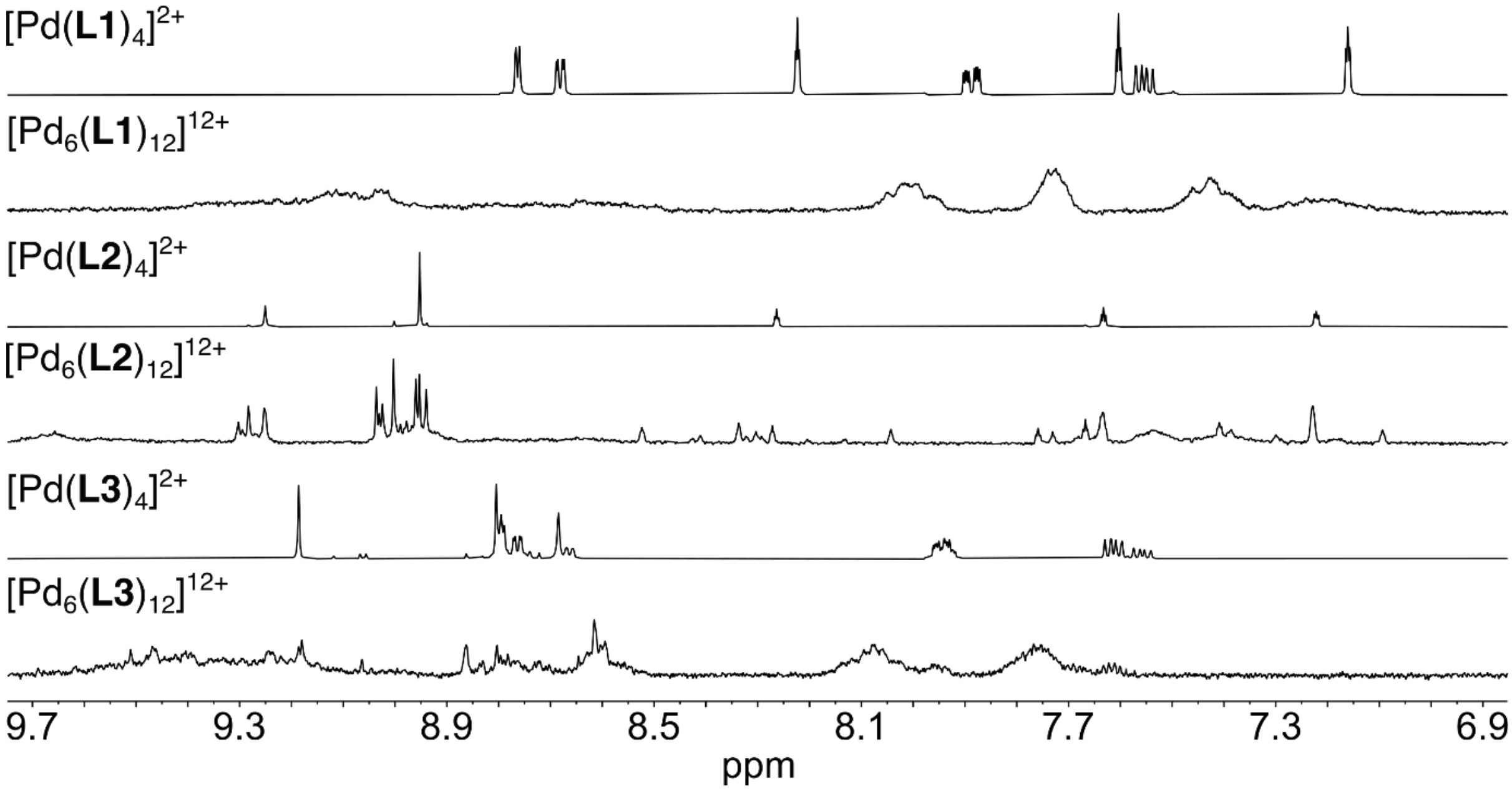

Figure S17. ^{1}H NMR spectrum (400 MHz, CD_3CN) of [Pd(**L1–3**)$_4$](BF_4)$_2$ stacked with [Pd$_6$(**L1–3**)$_{12}$](BF_4)$_{12}$.

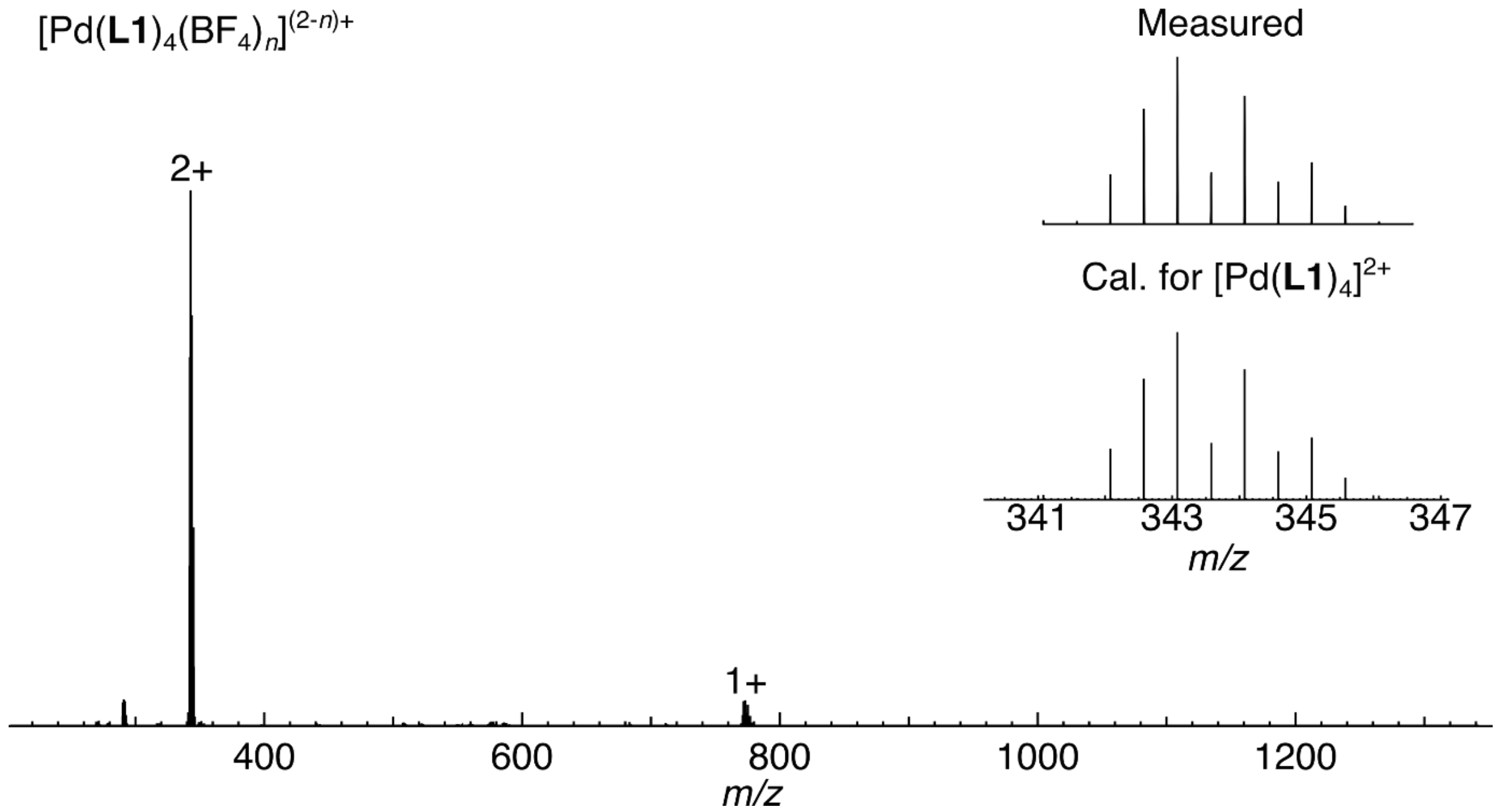

Figure S18. High-resolution ESI mass spectrum of [Pd(**L1**)$_4$(BF$_4$)$_n$]$^{(2-n)+}$.

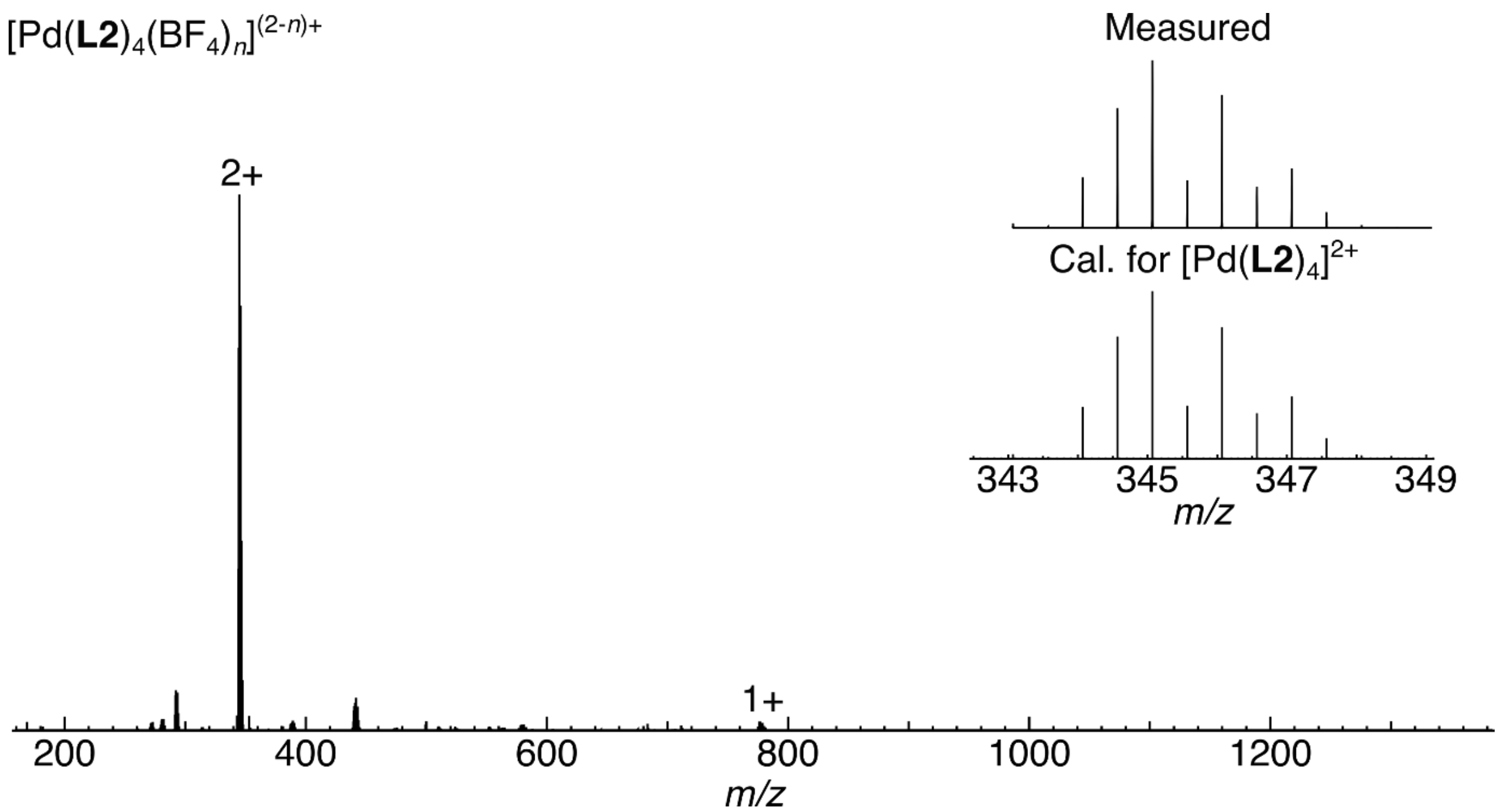

Figure S19. High-resolution ESI mass spectrum of [Pd(**L2**)$_4$(BF$_4$)$_n$]$^{(2-n)+}$.

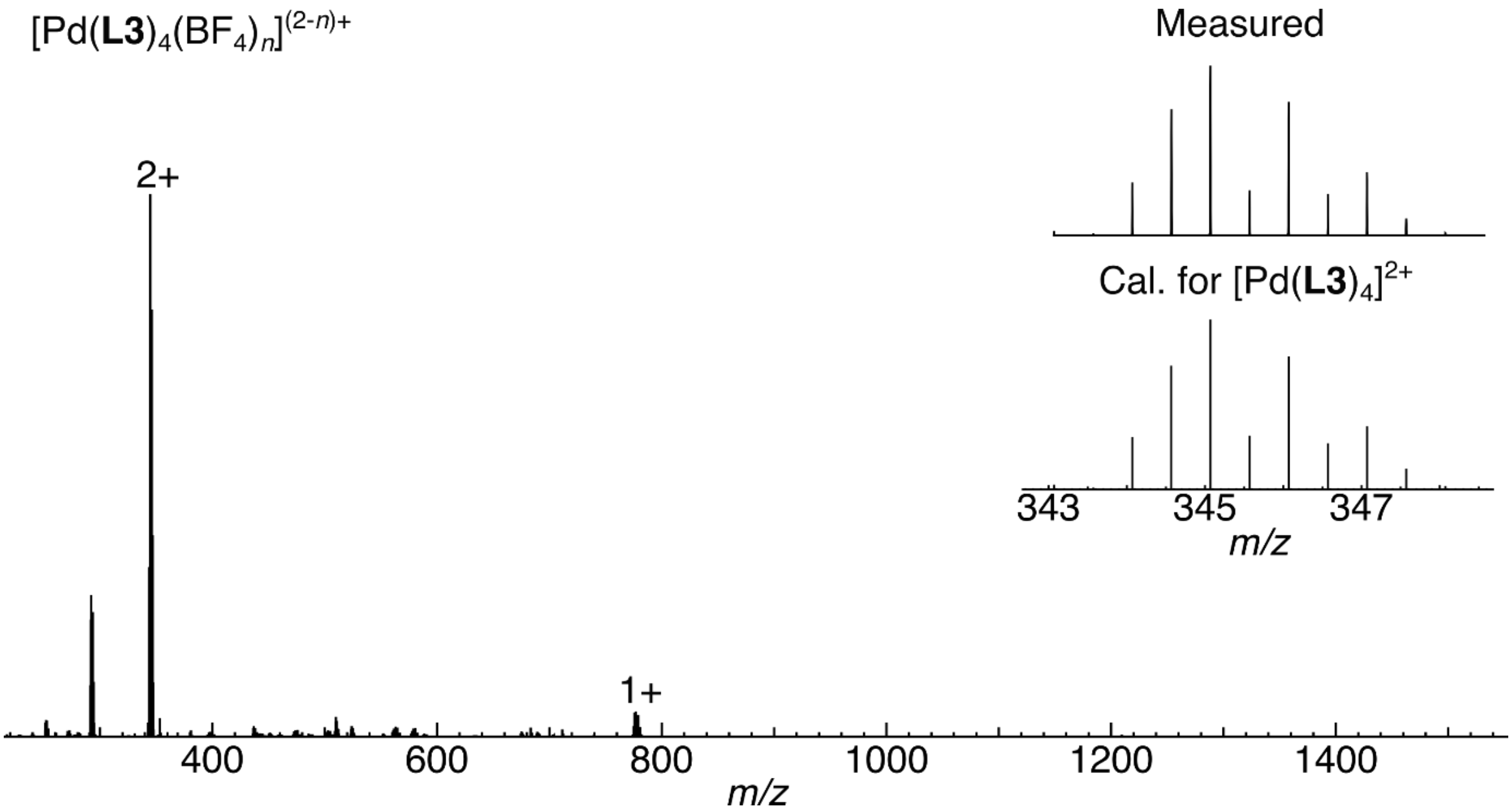

Figure S20. High-resolution ESI mass spectrum of [Pd(**L3**)$_4$(BF$_4$)$_n$]$^{(2-n)+}$.

3 Complex and Heterometallic Cage Syntheses

3.1 Synthesis of $[Pt(\mathbf{L1})_4](BF_4)_2$

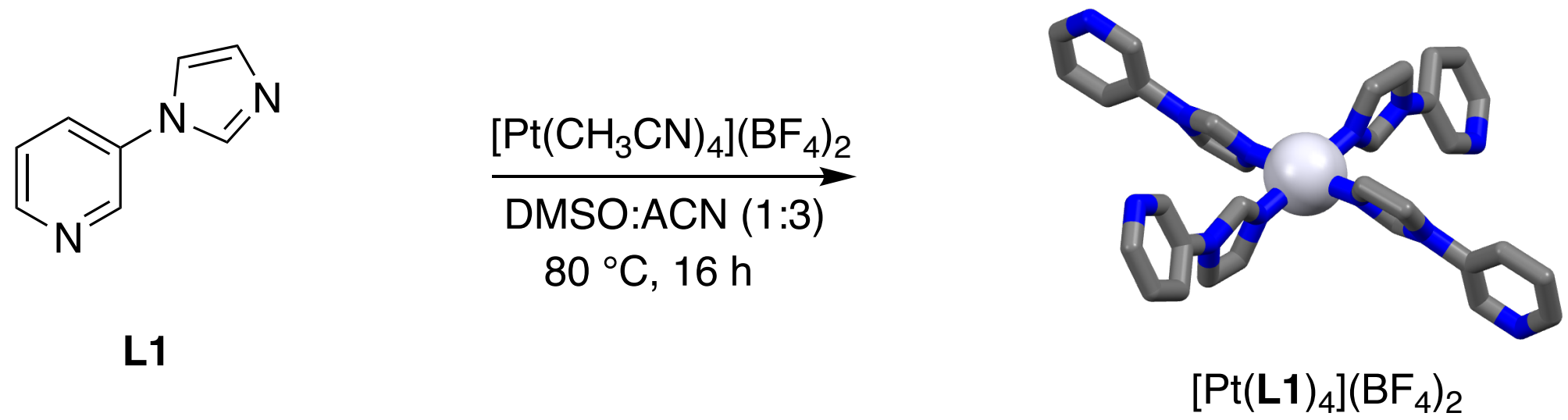

A solution of *cis*-$PtCl_2(DMSO)_2$ in DMSO-d_6 (80 mM, 2 mL, 160 μmol) was mixed with a solution of $AgBF_4$ in dry acetonitrile (160 mM, 2 mL). After 30 min, the precipitate was removed using a syringe filter to yield a 40 mM solution of $[Pt(CH_3CN)_4](BF_4)_2$ in CH_3CN:DMSO-d_6 (1:1). Ligand **L1** (20 mg, 138 μmol, 4 eq.), acetonitrile (861 μL), and $[Pt(CH_3CN)_4](BF_4)_2$ (40 mM, 861 μL, 34.4 μmol, 1 eq.) were combined and the mixture was heated at 80 °C overnight. The mixture was allowed to cool to RT and was filtered using a syringe filter (0.22 μm). The product was precipitated with toluene (13 mL) and washed with toluene (14 mL) using a centrifuge. The product was dried in air overnight and then dried under reduced pressure at RT for 1 h. $[Pt(\mathbf{L1})_4](BF_4)_2$ was obtained as a white powder (24.0 mg, 25.2 μmol, 73 %). ^{1}H (400 MHz, CD_3CN) δ 8.79 (dd, *J* = 2.7, 0.8 Hz, 4H), 8.69 (dd, *J* = 4.8, 1.4 Hz, 4H), 8.33 (t, *J* = 1.4 Hz, 4H), 7.92 (ddd, *J* = 8.3, 2.8, 1.4 Hz, 4H), 7.65 (t, *J* = 1.7 Hz, 4H), 7.56 (ddd, *J* = 8.3, 4.8, 0.8 Hz, 4H), 7.19 (dd, *J* = 1.9, 1.3 Hz, 4H). $^{13}C\{^1H\}$ (101 MHz, CD_3CN) δ 151.24, 144.28, 139.26, 133.42, 130.80, 125.40, 121.77.

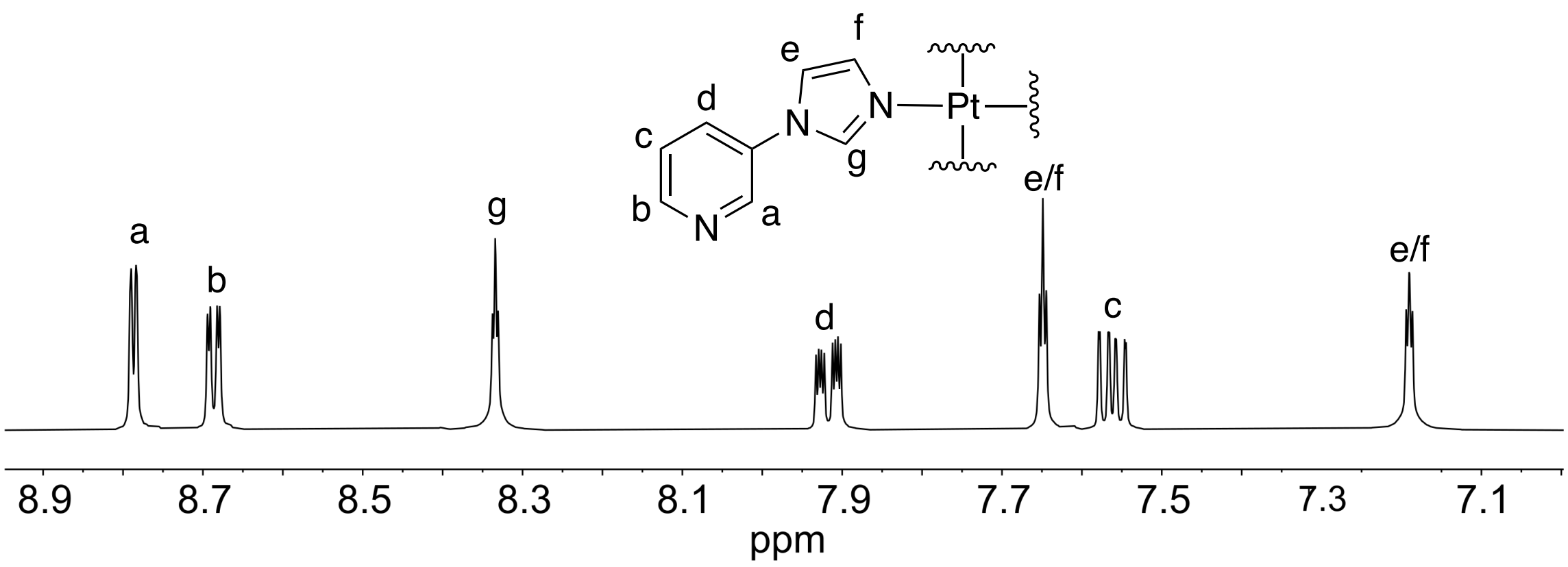

Figure S21. ^{1}H NMR spectrum (400 MHz, CD_3CN) of $[Pt(\mathbf{L1})_4](BF_4)_2$.

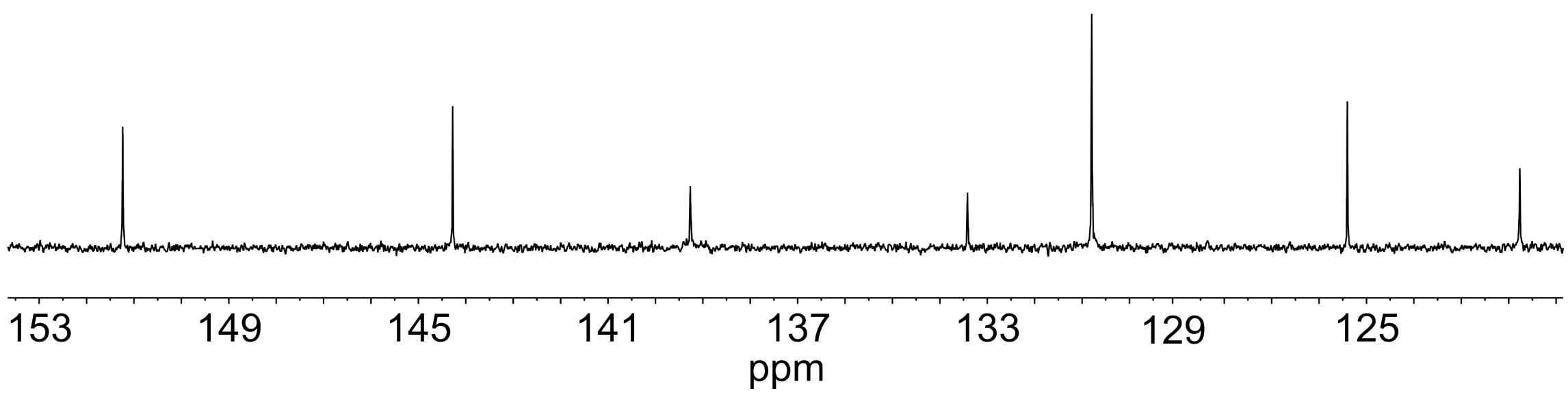

Figure S22. $^{13}C\{^1H\}$ NMR spectrum (101 MHz, CD_3CN) of $[Pt(\mathbf{L1})_4](BF_4)_2$.

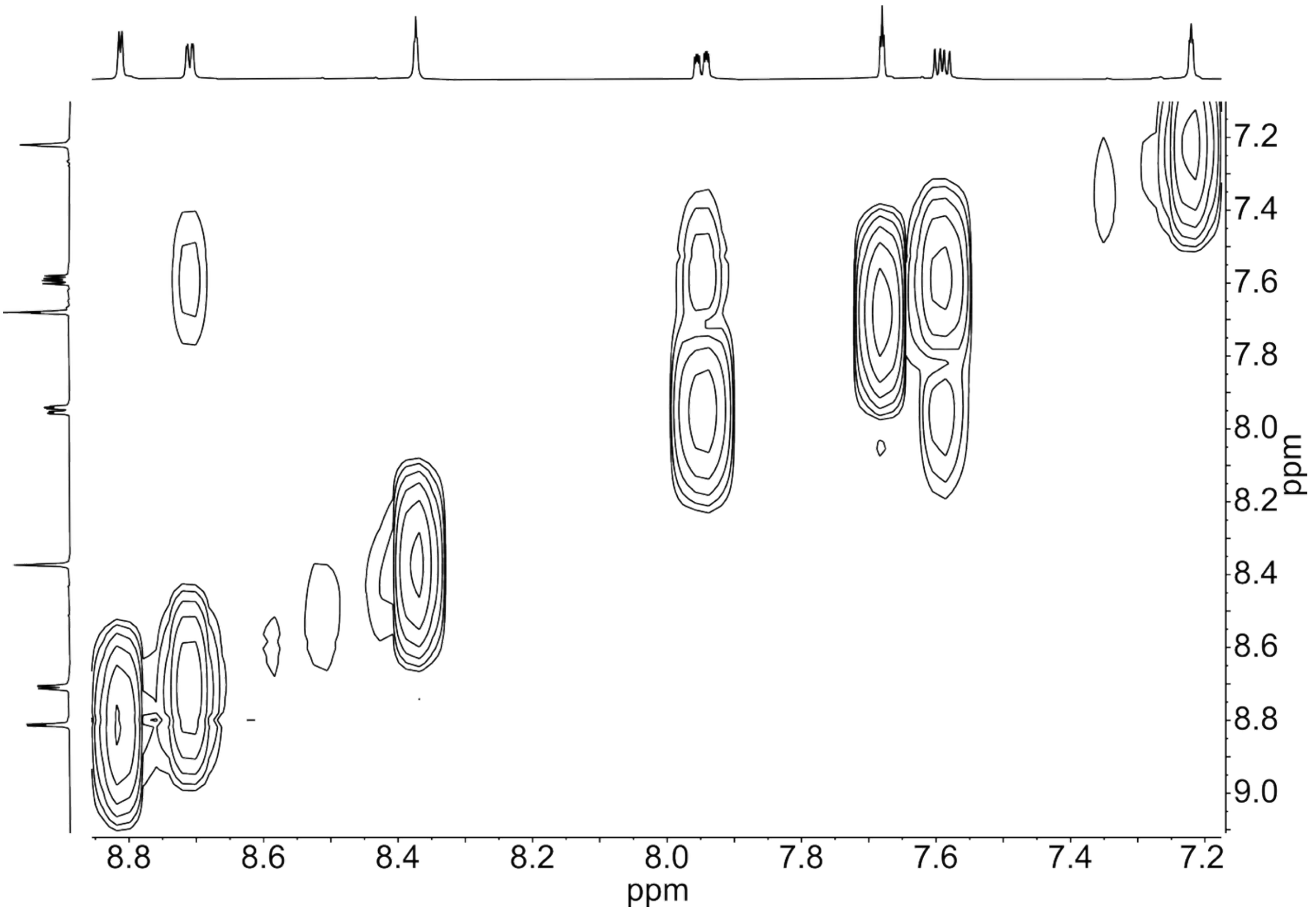

Figure S23. ^1H-^1H COSY NMR spectrum (600 MHz, CD_3CN) of $[Pt(\mathbf{L1})_4](BF_4)_2$.

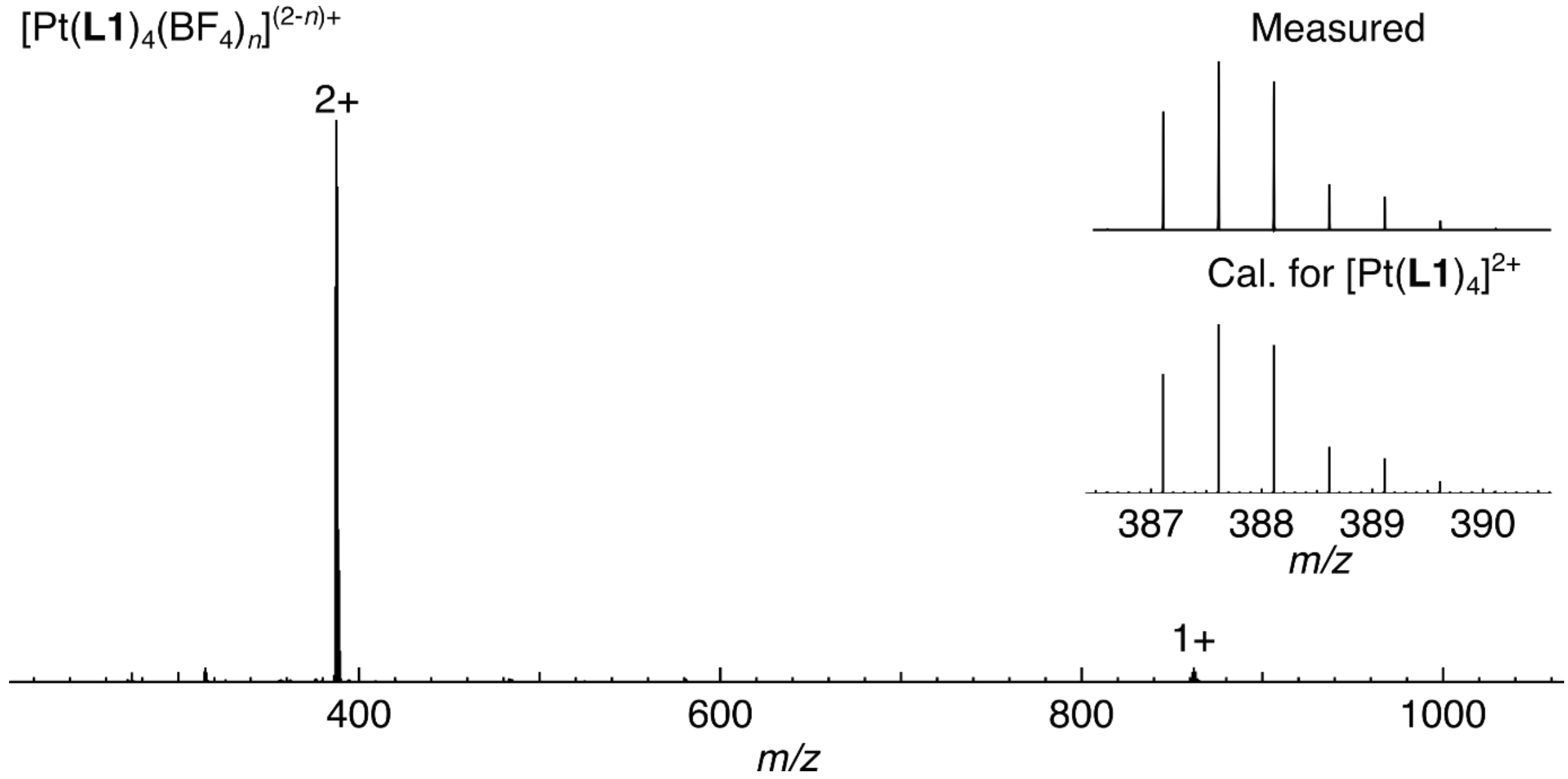

Figure S24. High-resolution ESI mass spectrum of $[Pt(\mathbf{L1})_4](BF_4)_n]^{(2-n)+}$.

3.2 Synthesis of $[Pt(\mathbf{L2})_4](BF_4)_2$

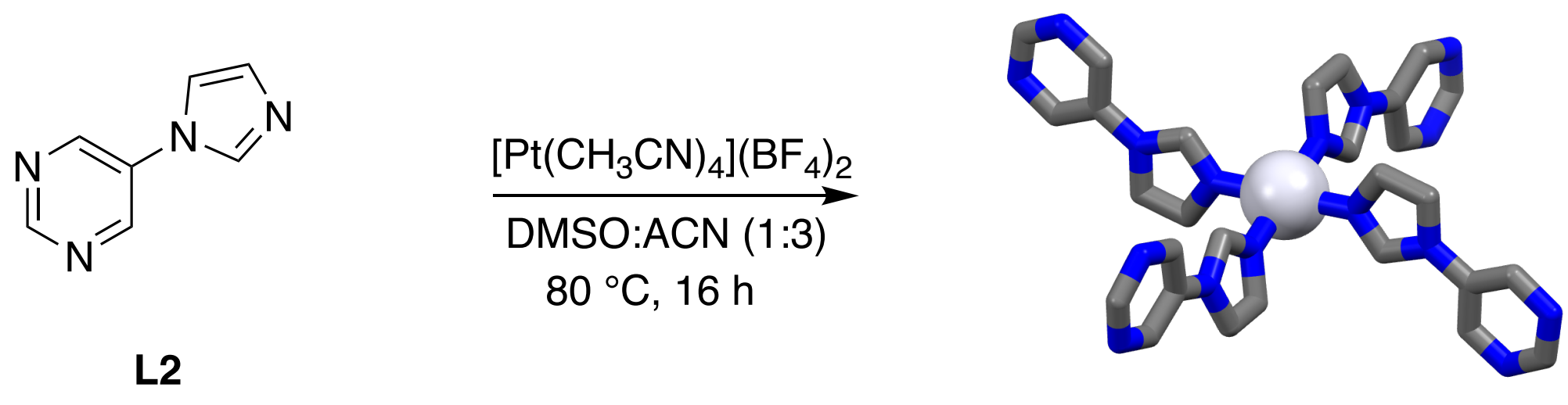

$[Pt(\mathbf{L2})_4](BF_4)_2$

A solution of *cis*-$PtCl_2(DMSO)_2$ in DMSO-d_6 (80 mM, 2 mL, 160 μmol) was mixed a solution of $Ag(BF_4)$ in dry acetonitrile (160 mM, 2 mL). After 30 min, the precipitate was removed using a syringe filter to yield a 40 mM solution of $[Pt(CH_3CN)_4](BF_4)_2$ in CH_3CN:DMSO-d_6 (1:1). Ligand **L2** (20.1 mg, 138 μmol, 4 eq.), acetonitrile (861 μL), and $[Pt(CH_3CN)_4](BF_4)_2$ (40 mM, 861 μL, 34.4 μmol, 1 eq.) were combined and the mixture was heated at 80 °C overnight. The mixture was allowed to cool to RT and was filtered using a syringe filter (0.22 μm). The product was precipitated with toluene (13 mL) and washed with toluene (14 mL) using a centrifuge. The product was dried in air overnight and then dried under reduced pressure at RT for 1 h. $[Pt(\mathbf{L2})_4](BF_4)_2$ was obtained as a white powder (30.0 mg, 31.5 μmol, 91 %). ^1H (400 MHz, CD_3CN) δ 9.25 (s, 4H), 8.98 (s, 8H), 8.39 (t, J = 1.4 Hz, 4H), 7.68 (t, J = 1.7 Hz, 4H), 7.26 (dd, J = 1.9, 1.3 Hz, 4H). $^{13}C\{^1H\}$ (101 MHz, CD_3CN) δ 159.43, 151.67, 139.39, 132.20, 131.10, 121.72.

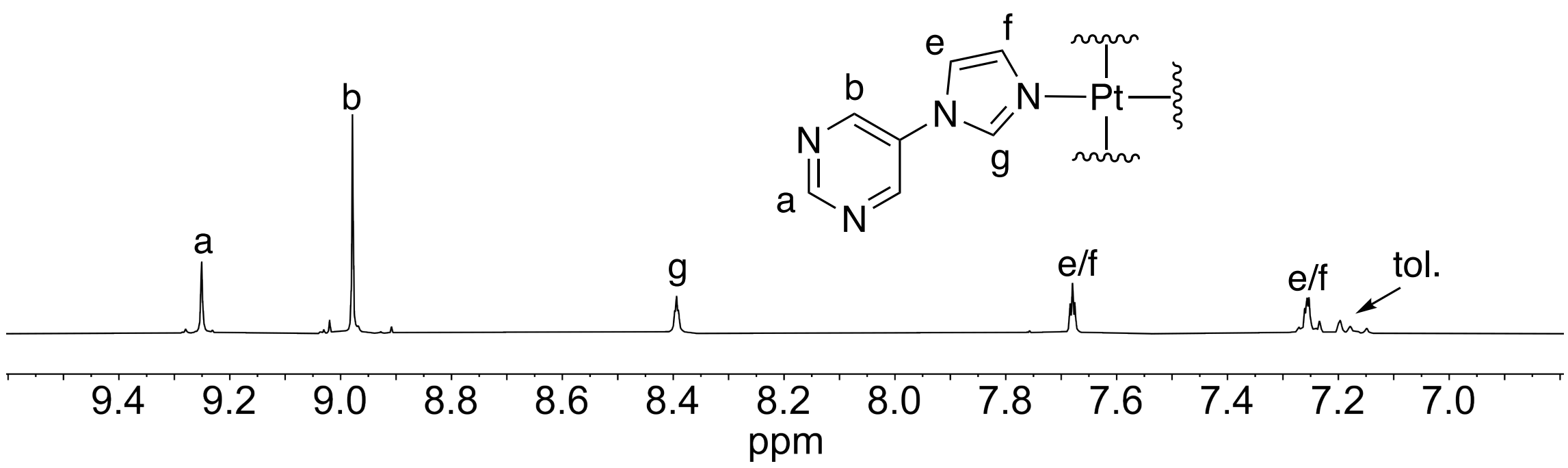

Figure S25. ^1H NMR spectrum (400 MHz, CD_3CN) of $[Pt(\mathbf{L2})_4](BF_4)_2$.

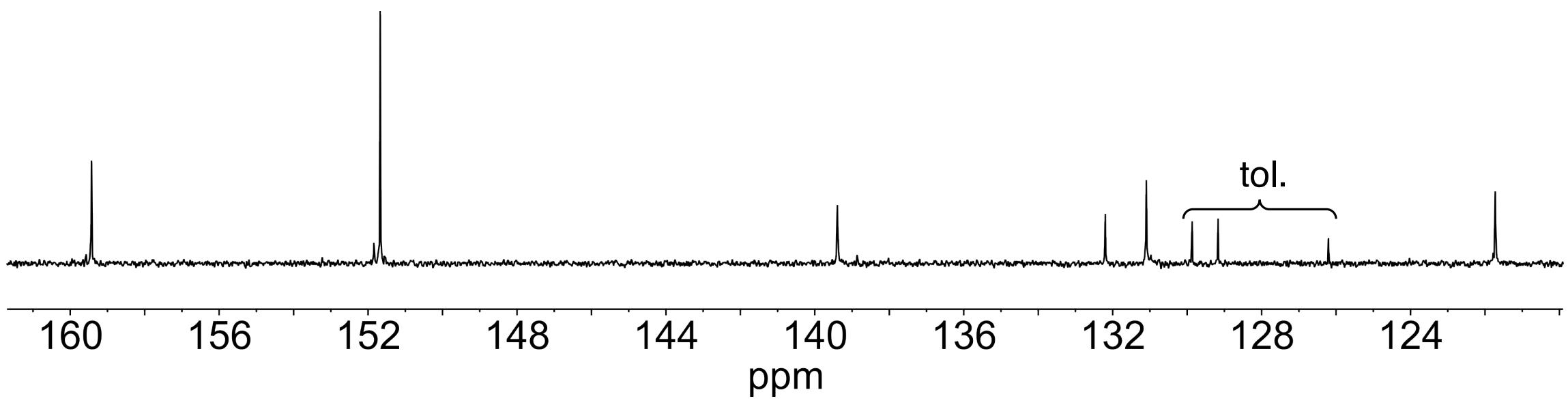

Figure S26. $^{13}C\{^1H\}$ NMR spectrum (101 MHz, CD_3CN) of $[Pt(\mathbf{L2})_4](BF_4)_2$.

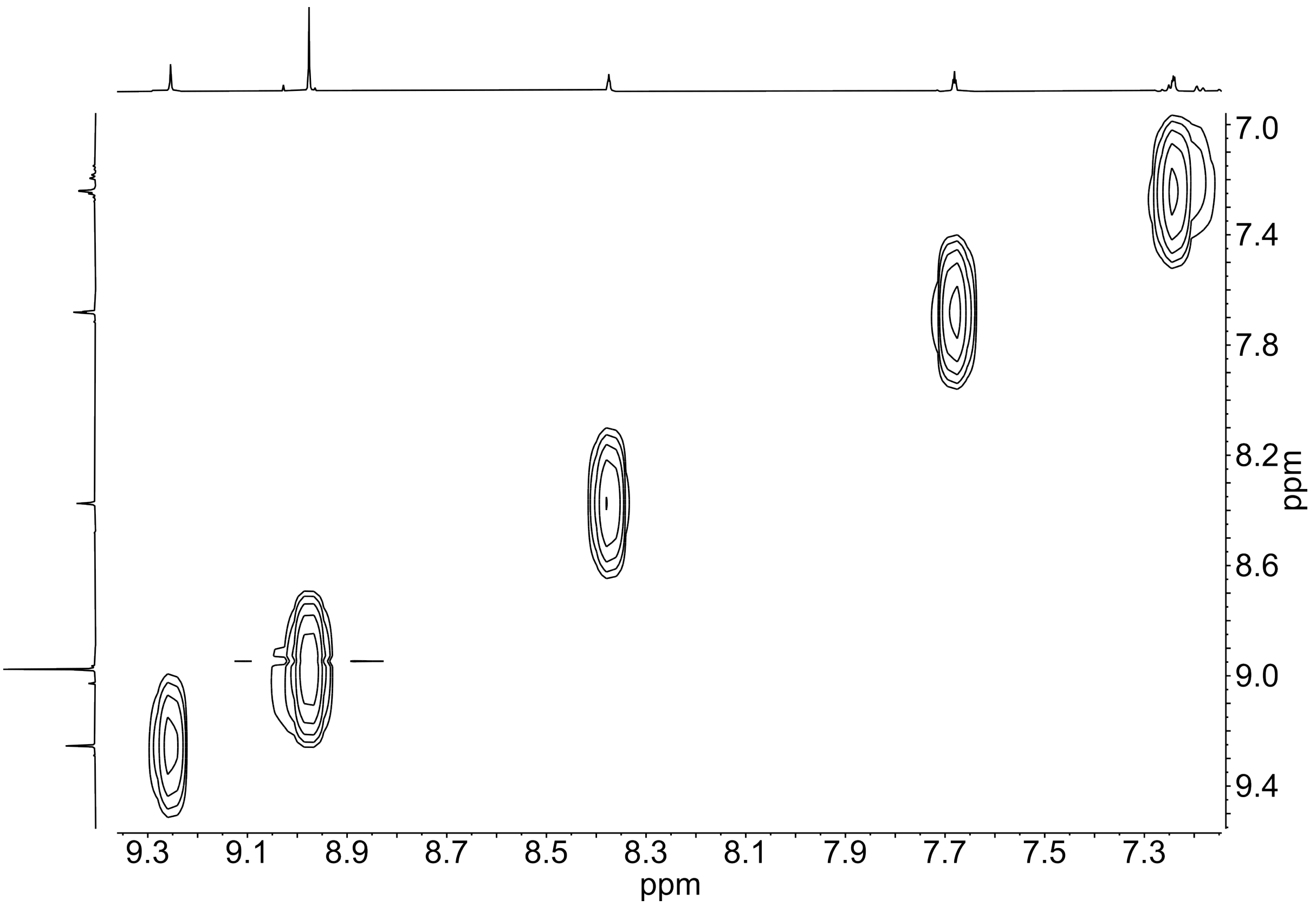

Figure S27. ^{1}H-^{1}H COSY NMR spectrum (600 MHz, CD_3CN) of [Pt(**L2**)$_4$](BF_4)$_2$.

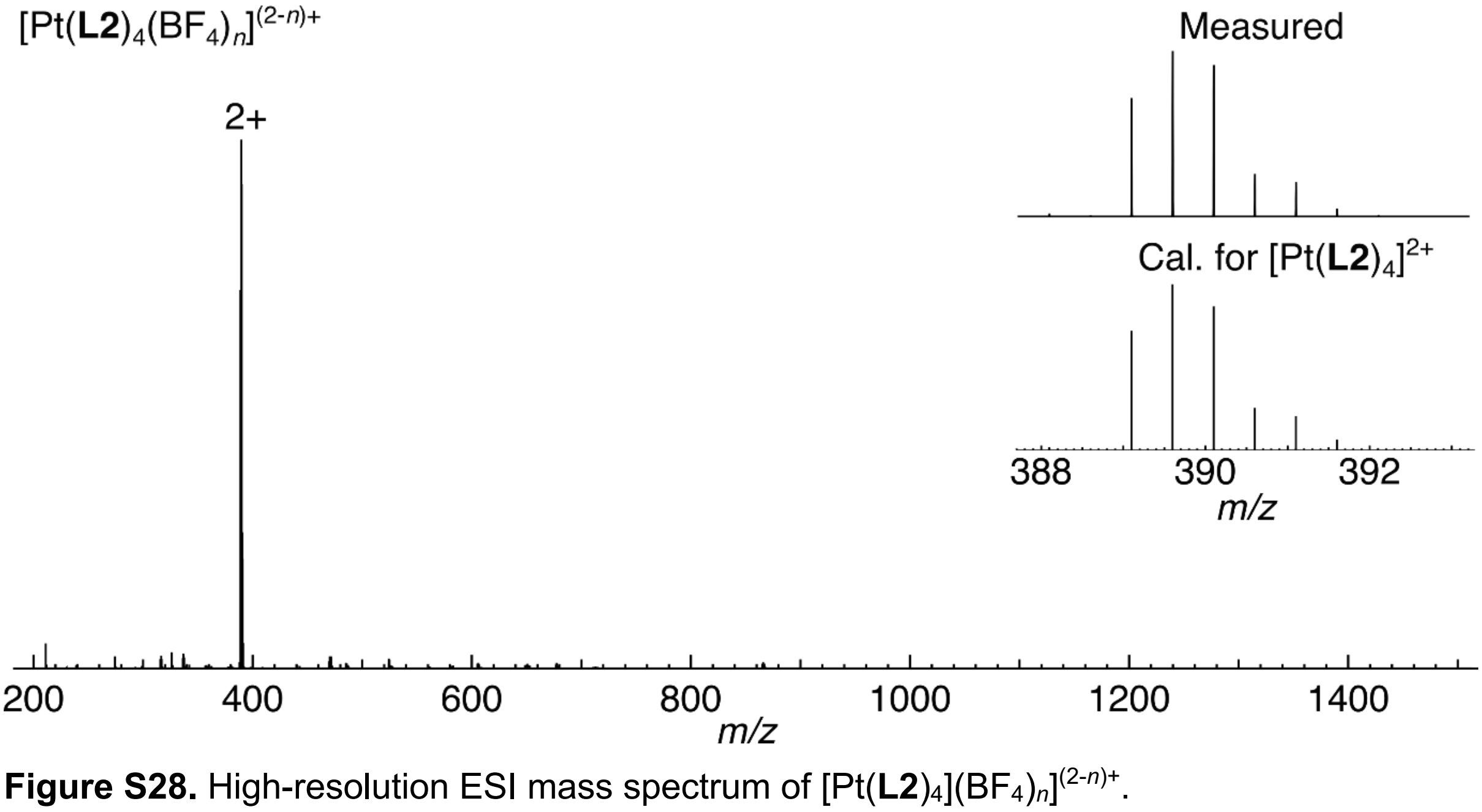

Figure S28. High-resolution ESI mass spectrum of [Pt(**L2**)$_4$](BF_4)$_n$]$^{(2-n)+}$.

3.3 Synthesis of $[Pt(\mathbf{L3})_4](BF_4)_2$

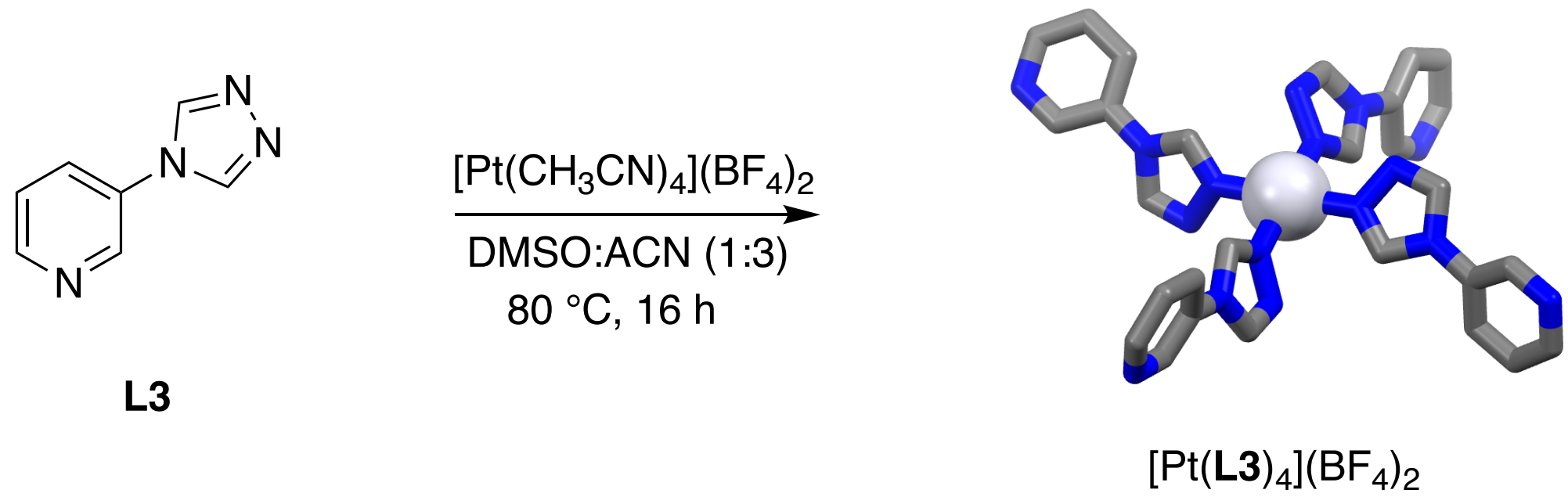

A solution of *cis*-$PtCl_2(DMSO)_2$ in DMSO-d_6 (80 mM, 2 mL, 160 µmol) was mixed with a solution of $Ag(BF_4)$ in dry acetonitrile (160 mM, 2 mL). After 30 min, the precipitate was removed using a syringe filter to yield a 40 mM solution of $[Pt(CH_3CN)_4](BF_4)_2$ in CH_3CN:DMSO-d_6 (1:1). Ligand **L3** (20.1 mg, 138 µmol, 4 eq.), acetonitrile (861 µL), and $[Pt(CH_3CN)_4](BF_4)_2$ (40 mM, 861 µL, 34.4 µmol, 1 eq.) were combined and the mixture was heated at 80 °C overnight. The mixture was allowed to cool to RT and was filtered using a syringe filter (0.22 µm). The product was precipitated with toluene (13 mL) and washed with toluene (14 mL) using a centrifuge. The product was dried in air overnight and then dried under reduced pressure at RT for 1 h. $[Pt(\mathbf{L3})_4](BF_4)_2$ was obtained as a white powder (25.7 mg, 27.0 µmol, 78 %). ^1H (400 MHz, CD_3CN) δ 9.29 (s, 4H), 8.85 (s, 4H), 8.82 (dd, J = 2.8, 0.8 Hz, 4H), 8.77 (dd, J = 4.8, 1.4 Hz, 4H), 7.98 (ddd, J = 8.3, 2.7, 1.4 Hz, 4H), 7.62 (ddd, J = 8.3, 4.8, 0.8 Hz, 4H). $^{13}C\{^1H\}$ (101 MHz, CD_3CN) δ 152.28, 145.59, 144.80, 143.69, 131.71, 130.63, 125.61.

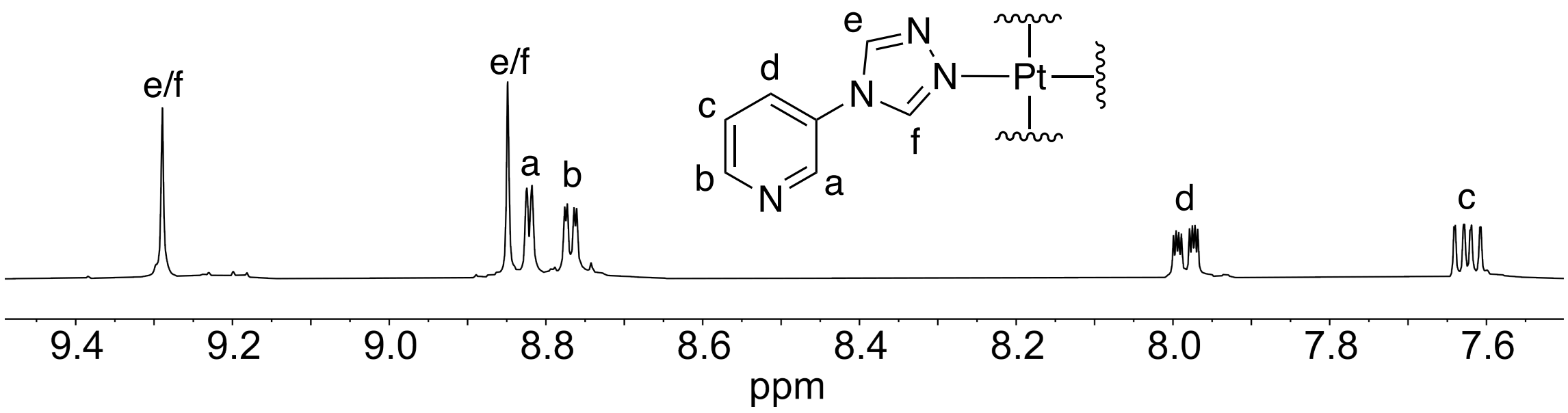

Figure S29. ^1H NMR spectrum (400 MHz, CD_3CN) of $[Pt(\mathbf{L3})_4](BF_4)_2$.

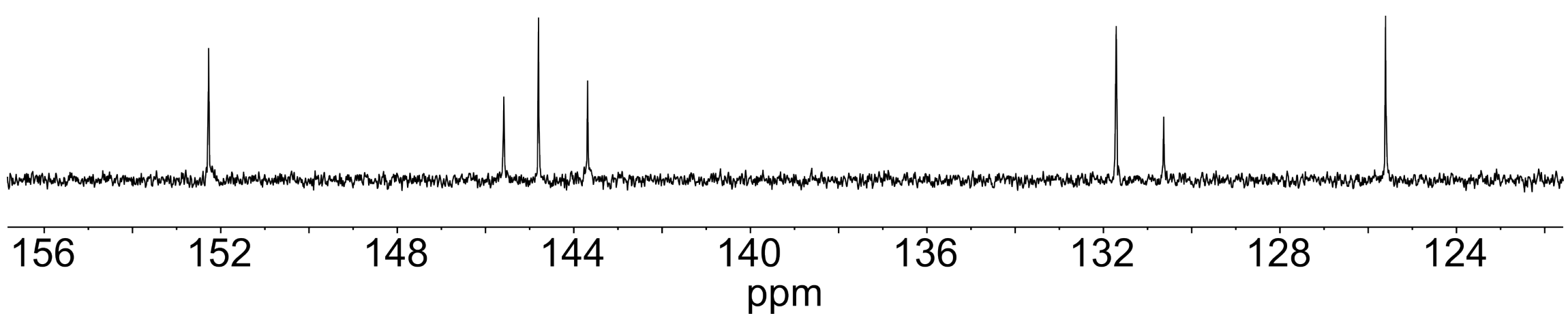

Figure S30. $^{13}C\{^1H\}$ NMR spectrum (101 MHz, CD_3CN) of $[Pt(\mathbf{L3})_4](BF_4)_2$.

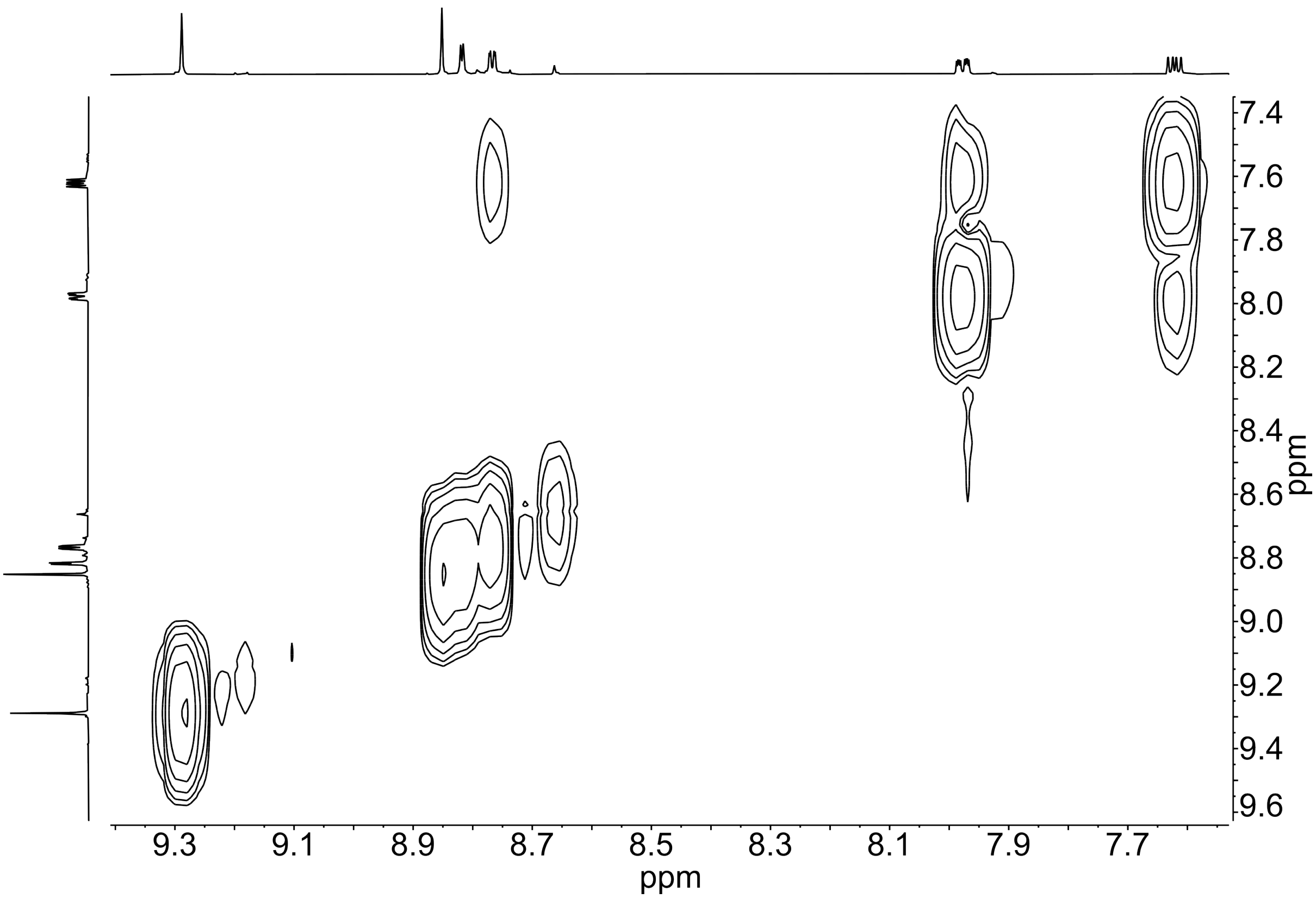

Figure S31. ^1H-^1H COSY NMR spectrum (600 MHz, CD_3CN) of [Pt(**L3**)$_4$](BF_4)$_2$.

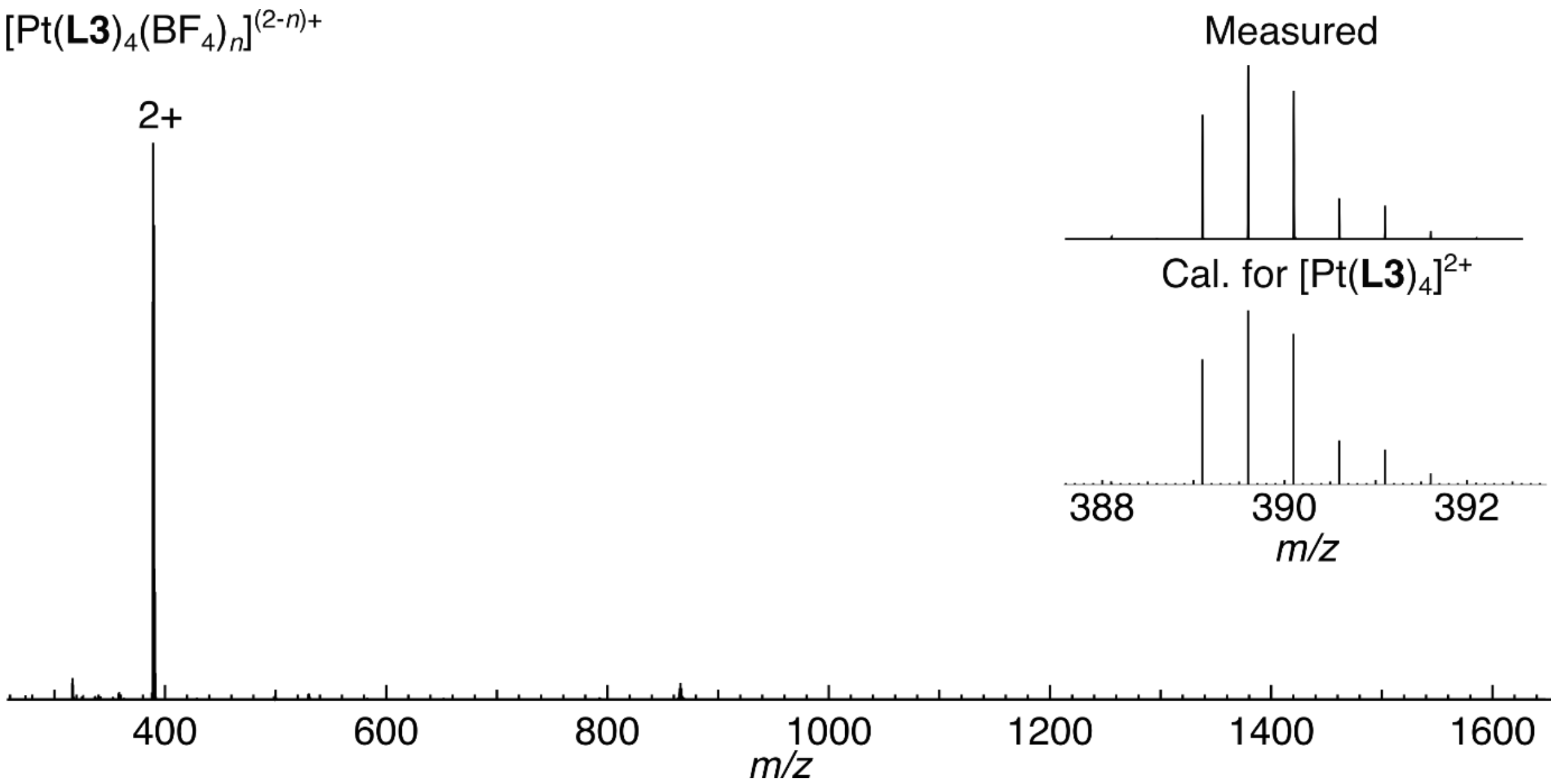

Figure S32. High-resolution ESI mass spectrum of [Pt(**L3**)$_4$](BF_4)$_n$]$^{(2-n)+}$.

3.4 Synthesis of $[Pt_2Pd_4(\mathbf{L1})_8Cl_8](BF_4)_4$

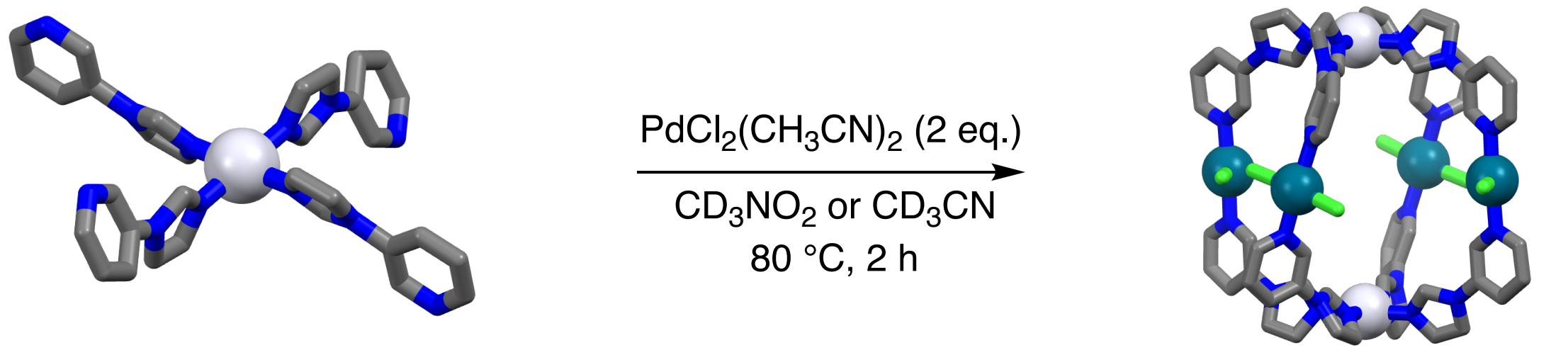

$[Pt(\mathbf{L1})_4](BF_4)_2$ $[Pt_2Pd_4(\mathbf{L1})_8Cl_8](BF_4)_4$

$[Pt(\mathbf{L1})_4](BF_4)_2$ (1.0 eq., 0.8 μmol, 200 μL of a 4 mM stock solution in CD_3NO_2), 320 μL CD_3NO_2, and $PdCl_2(CH_3CN)_2$ (2.0 eq., 1.6 μmol, 80 μL of a 20 mM stock solution in CD_3NO_2) were added to an NMR tube and heated at 80 °C for 20 h to give a 0.66 mM solution of cage $[Pt_2Pd_4(\mathbf{L1})_8Cl_8](BF_4)_4$. ^{1}H (600 MHz, CD_3NO_2) δ 9.12 (s, 8H), 8.94 (d, J = 5.6 Hz, 8H), 8.80 (s, 8H), 8.16 (dd, J = 8.2, 2.0 Hz, 8H), 7.73 (dd, J = 8.5, 5.6 Hz, 8H), 7.66 (t, J = 1.8 Hz, 8H), 7.36 (s, 8H). ^{13}C{^{1}H} (151 MHz, CD_3NO_2) δ 154.87, 147.40, 139.85, 135.21, 134.55, 131.61, 127.65, 122.34, 119.10.

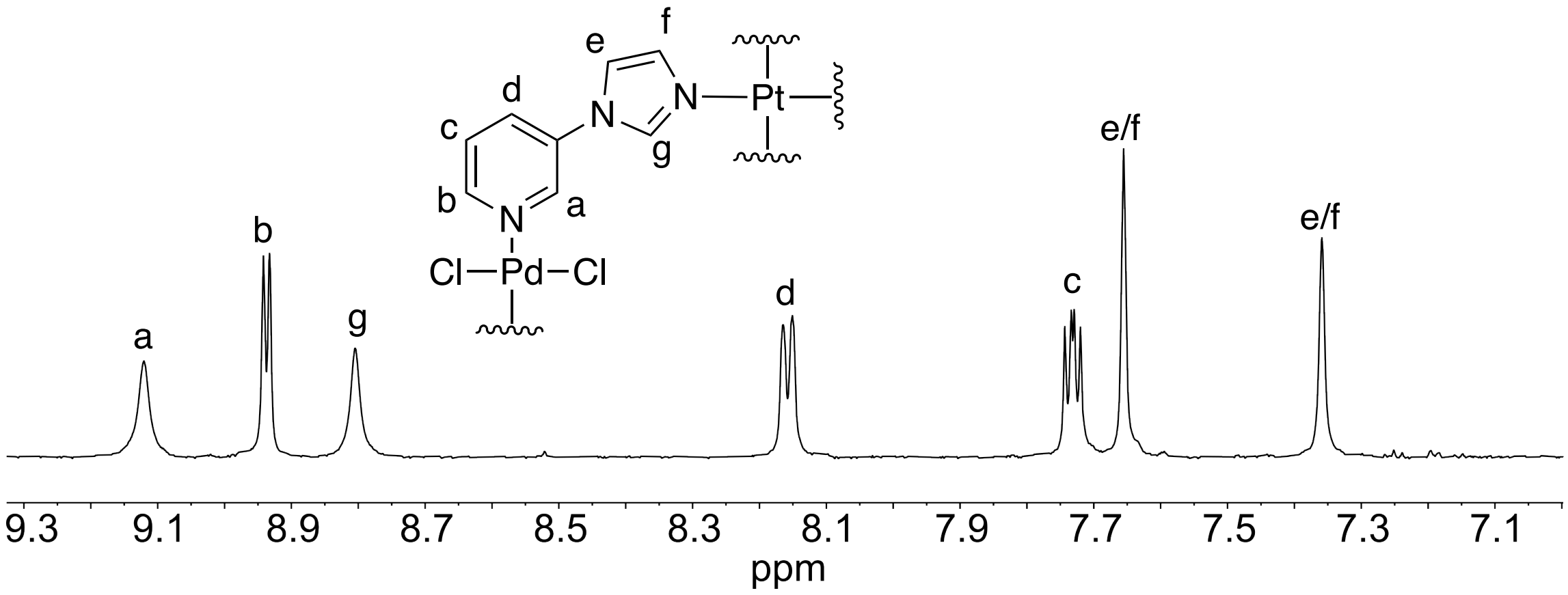

Figure S33. ^{1}H NMR spectrum (600 MHz, CD_3NO_2) of $[Pt_2Pd_4(\mathbf{L1})_8Cl_8](BF_4)_4$.

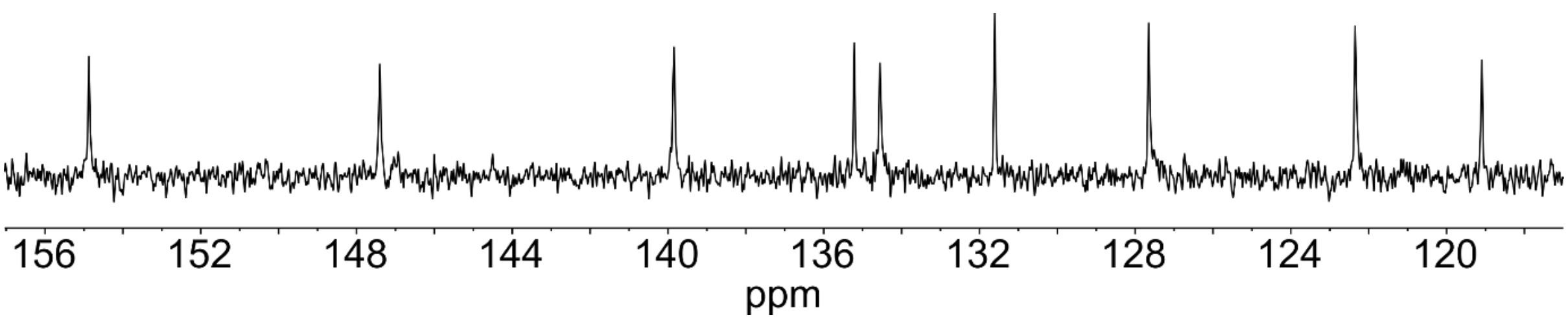

Figure S34. ^{13}C{^{1}H} NMR spectrum (151 MHz, CD_3NO_2) of $[Pt_2Pd_4(\mathbf{L1})_8Cl_8](BF_4)_4$.

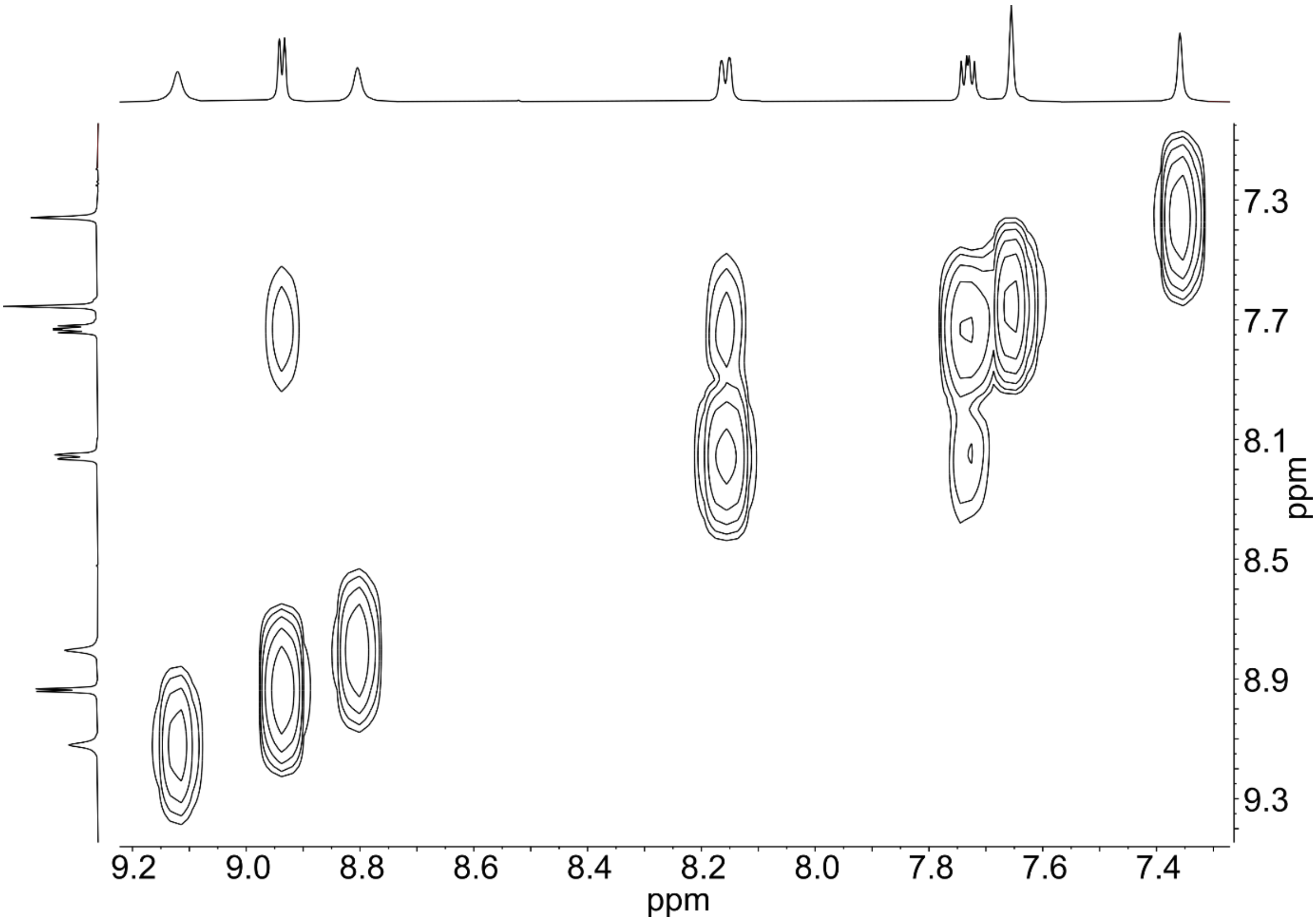

Figure S35. ^1H-^1H COSY NMR spectrum (600 MHz, CD_3NO_2) of $[Pt_2Pd_4(\mathbf{L1})_8Cl_8](BF_4)_4$.

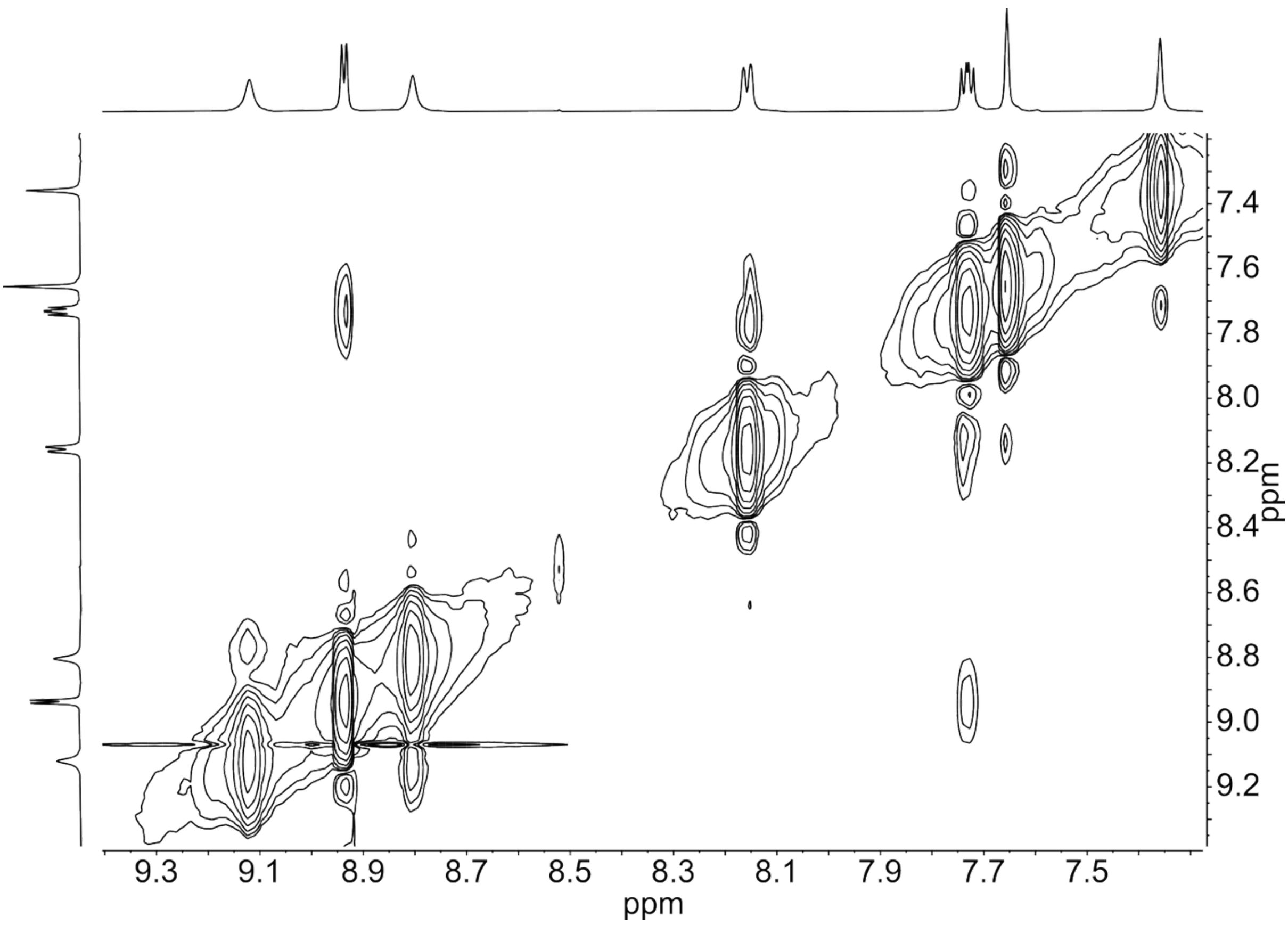

Figure S36. ^1H-^1H NOESY NMR spectrum (600 MHz, CD_3NO_2) of $[Pt_2Pd_4(\mathbf{L1})_8Cl_8](BF_4)_4$.

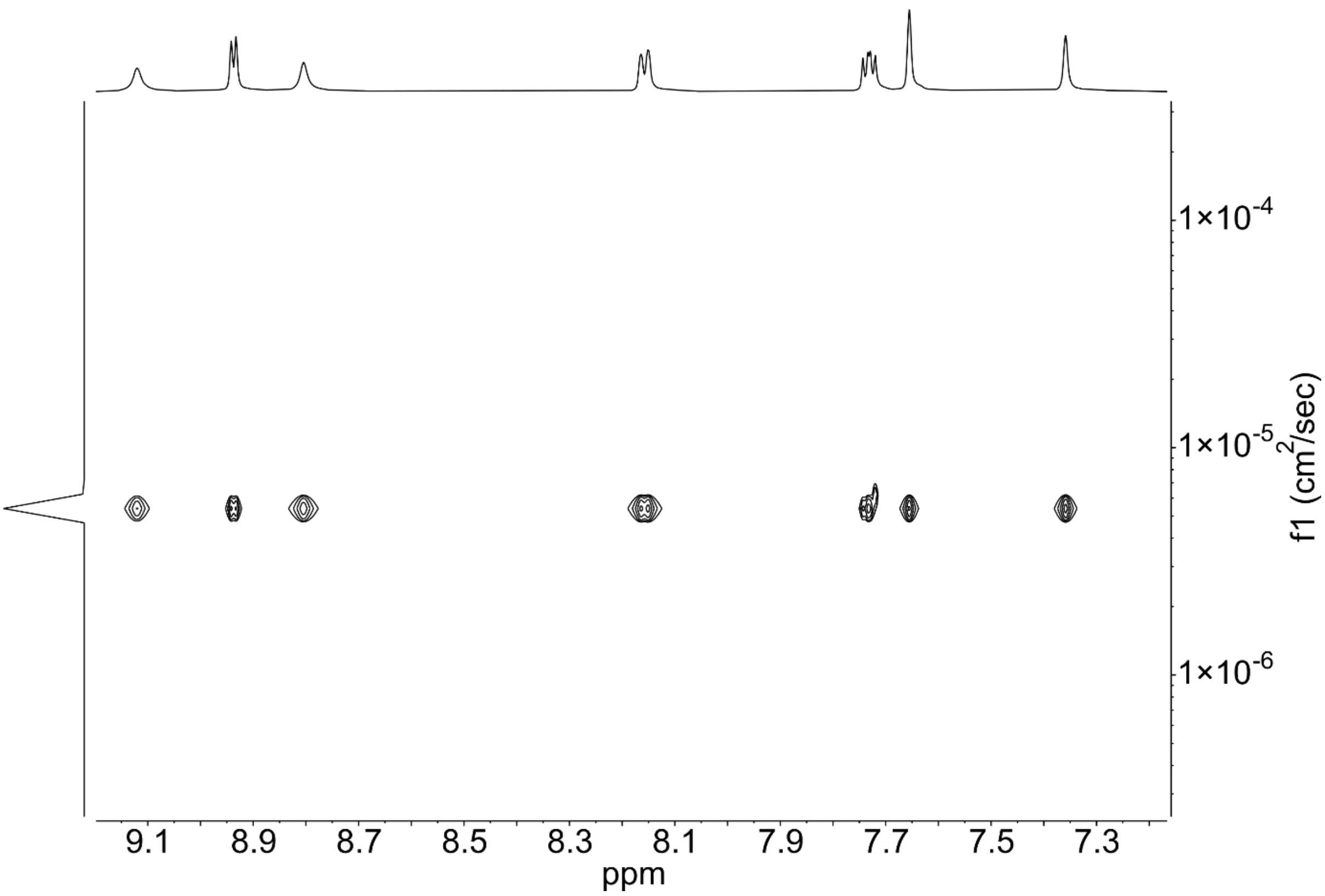

Figure S37. DOSY NMR spectrum (600 MHz, CD_3NO_2) of $[Pt_2Pd_4(\mathbf{L1})_8Cl_8](BF_4)_4$ (D = 5.40 x 10^{-6} cm^2 s^{-1}).

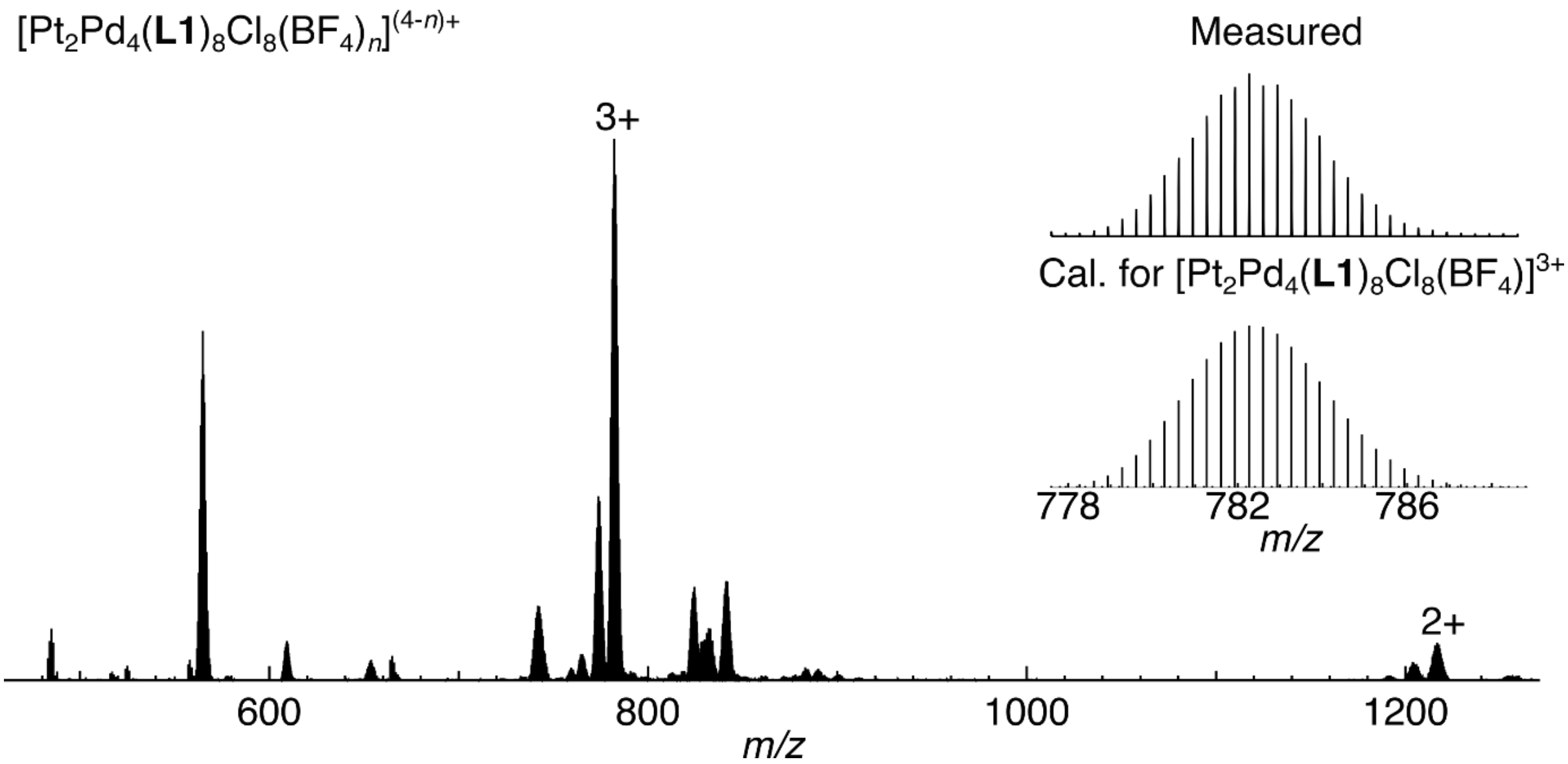

Figure S38. High-resolution ESI mass spectrum of $[Pt_2Pd_4(\mathbf{L1})_8Cl_8(BF_4)_n]^{(4-n)+}$.

3.5 Synthesis of $[Pt_2Pd_4(\mathbf{L2})_8Cl_8](BF_4)_4$

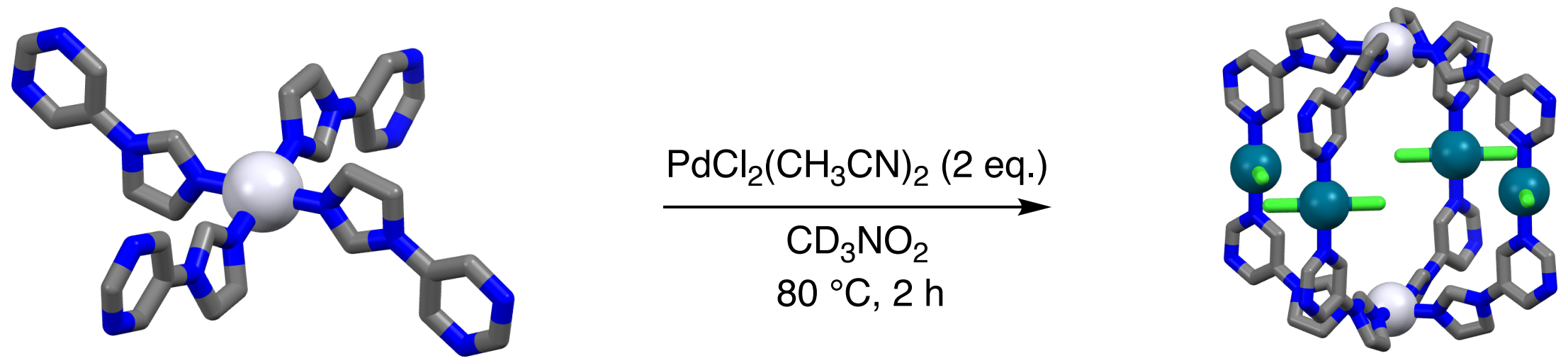

$[Pt(\mathbf{L2})_4](BF_4)_2$ $[Pt_2Pd_4(\mathbf{L2})_8Cl_8](BF_4)_4$

$[Pt(\mathbf{L2})_4](BF_4)_2$ (1.0 eq., 0.8 µmol, 200 µL of a 4 mM stock solution in CD_3NO_2), 320 µL CD_3NO_2, and $PdCl_2(CH_3CN)_2$ (2.0 eq., 1.6 µmol, 80 µL of a 20 mM stock solution in CD_3NO_2) were added to an NMR tube and heated at 80 °C for 20 h to give a 0.66 mM solution of cage $[Pt_2Pd_4(\mathbf{L1})_8Cl_8](BF_4)_4$. ^1H (600 MHz, CD_3CN) δ 9.55 (s, 8H), 9.41 (d, *J* = 2.9 Hz, 8H), 9.14 (d, *J* = 2.9 Hz, 8H), 8.84 (s, 8H), 7.75 (t, *J* = 1.8 Hz, 8H), 7.47 – 7.42 (m, 8H). $^{13}C\{^1H\}$ (151 MHz, CD_3CN) δ 161.52, 154.54, 140.19, 133.02, 131.93, 126.23, 122.22, 119.09.

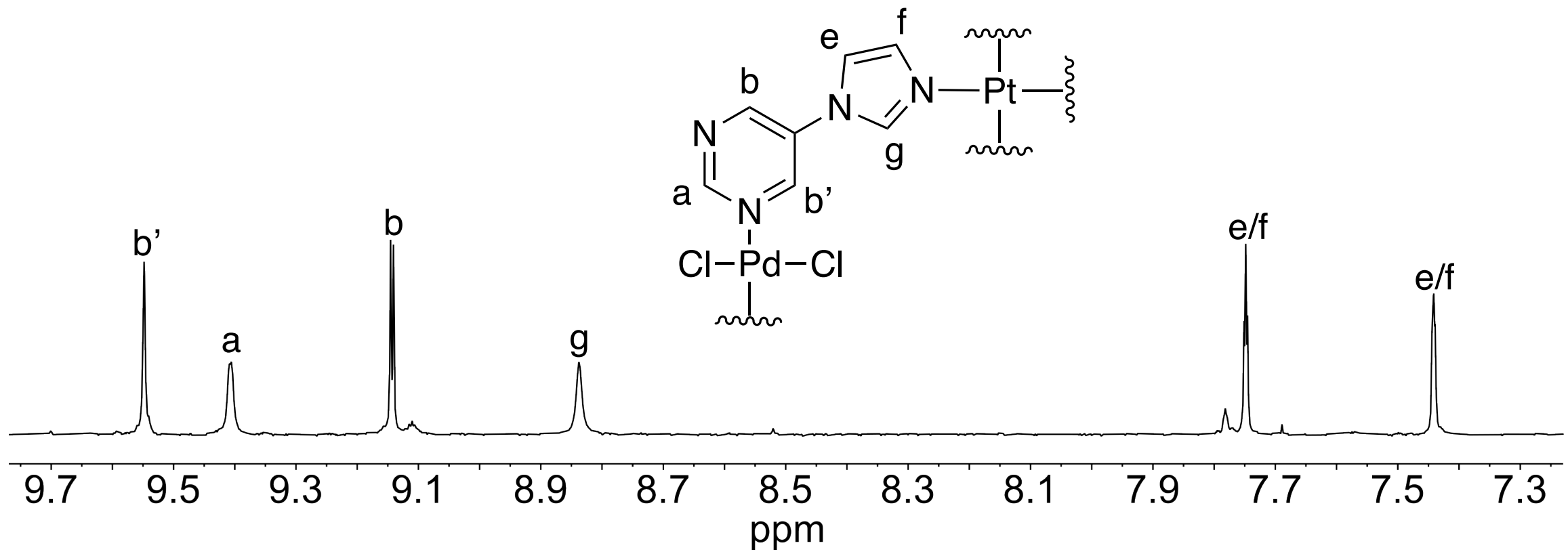

Figure S39. ^1H NMR spectrum (600 MHz, CD_3NO_2) of $[Pt_2Pd_4(\mathbf{L2})_8Cl_8](BF_4)_4$.

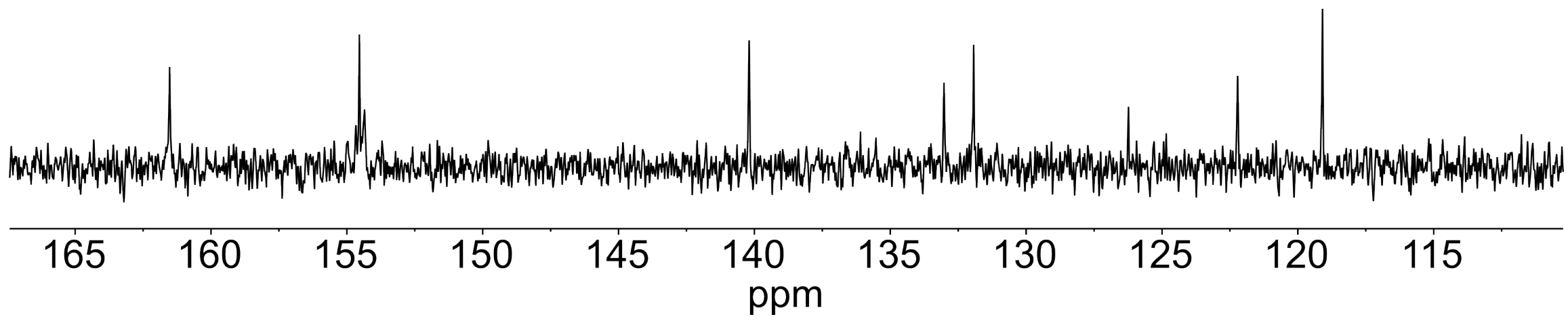

Figure S40. $^{13}C\{^1H\}$ NMR spectrum (151 MHz, CD_3NO_2) of $[Pt_2Pd_4(\mathbf{L2})_8Cl_8](BF_4)_4$.

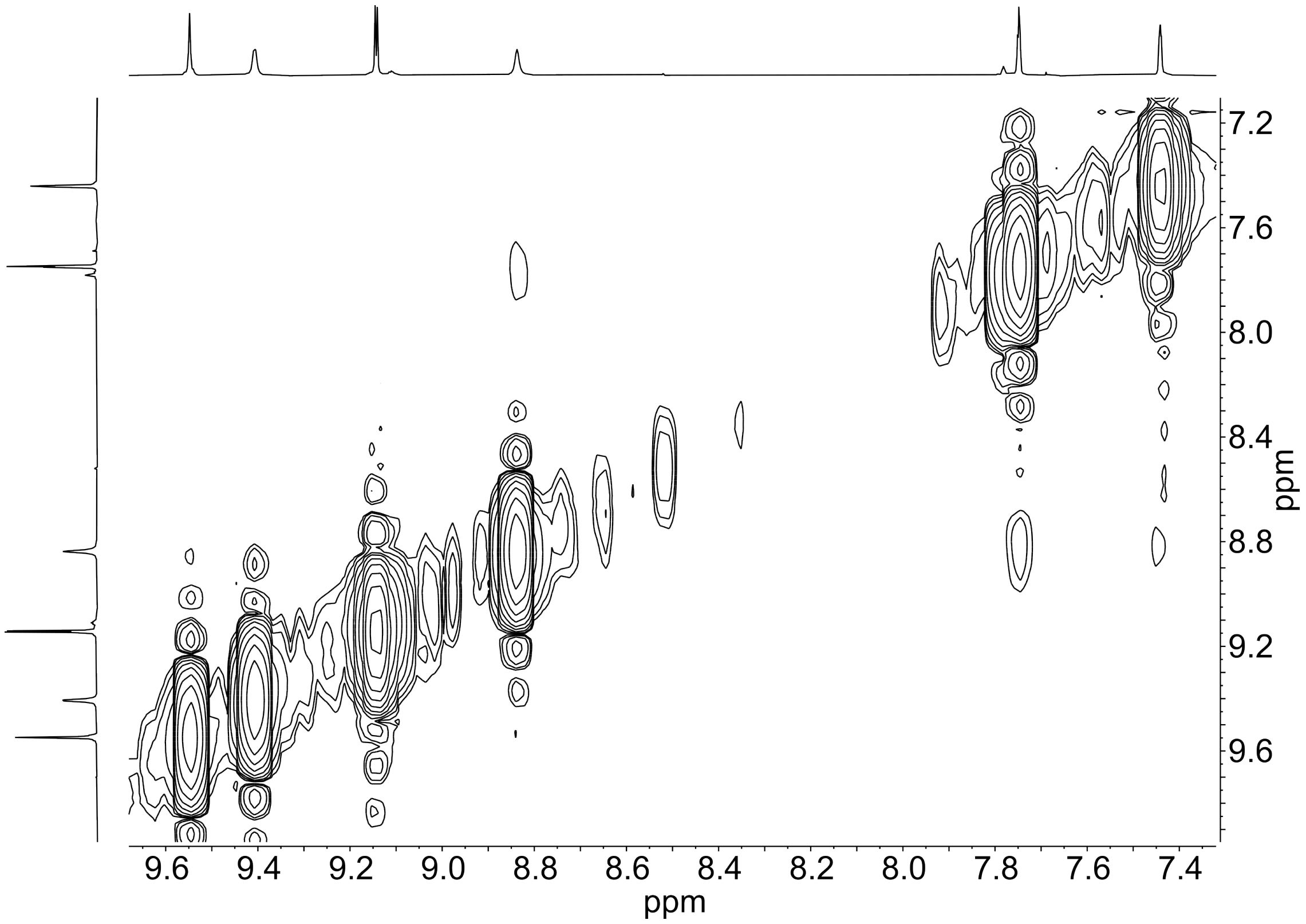

Figure S41. ^1H-^1H COSY NMR spectrum (600 MHz, CD_3NO_2) of $[Pt_2Pd_4(\mathbf{L2})_8Cl_8](BF_4)_4$.

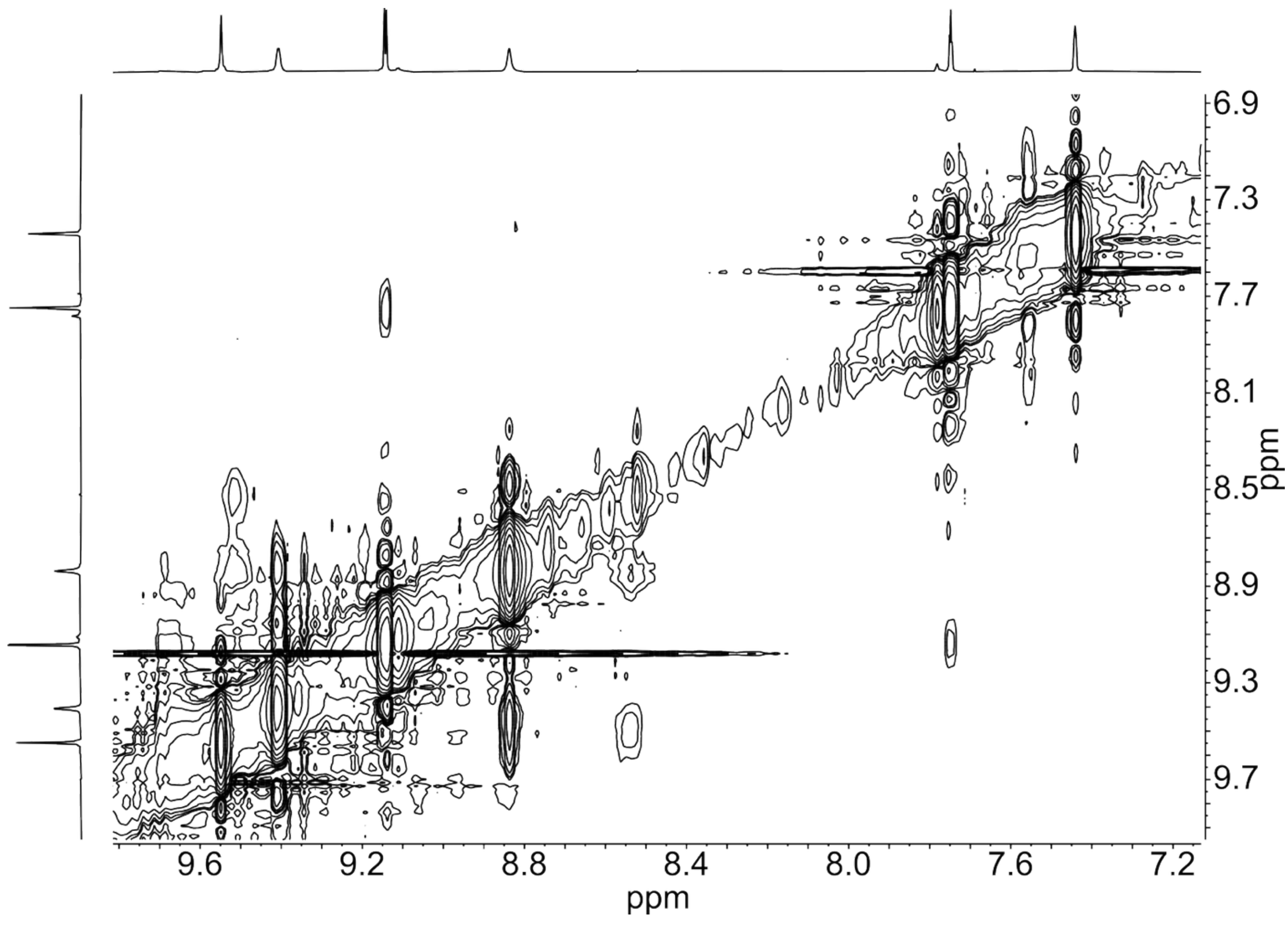

Figure S42. ^1H-^1H NOESY NMR spectrum (600 MHz, CD_3NO_2) of $[Pt_2Pd_4(\mathbf{L2})_8Cl_8](BF_4)_4$.

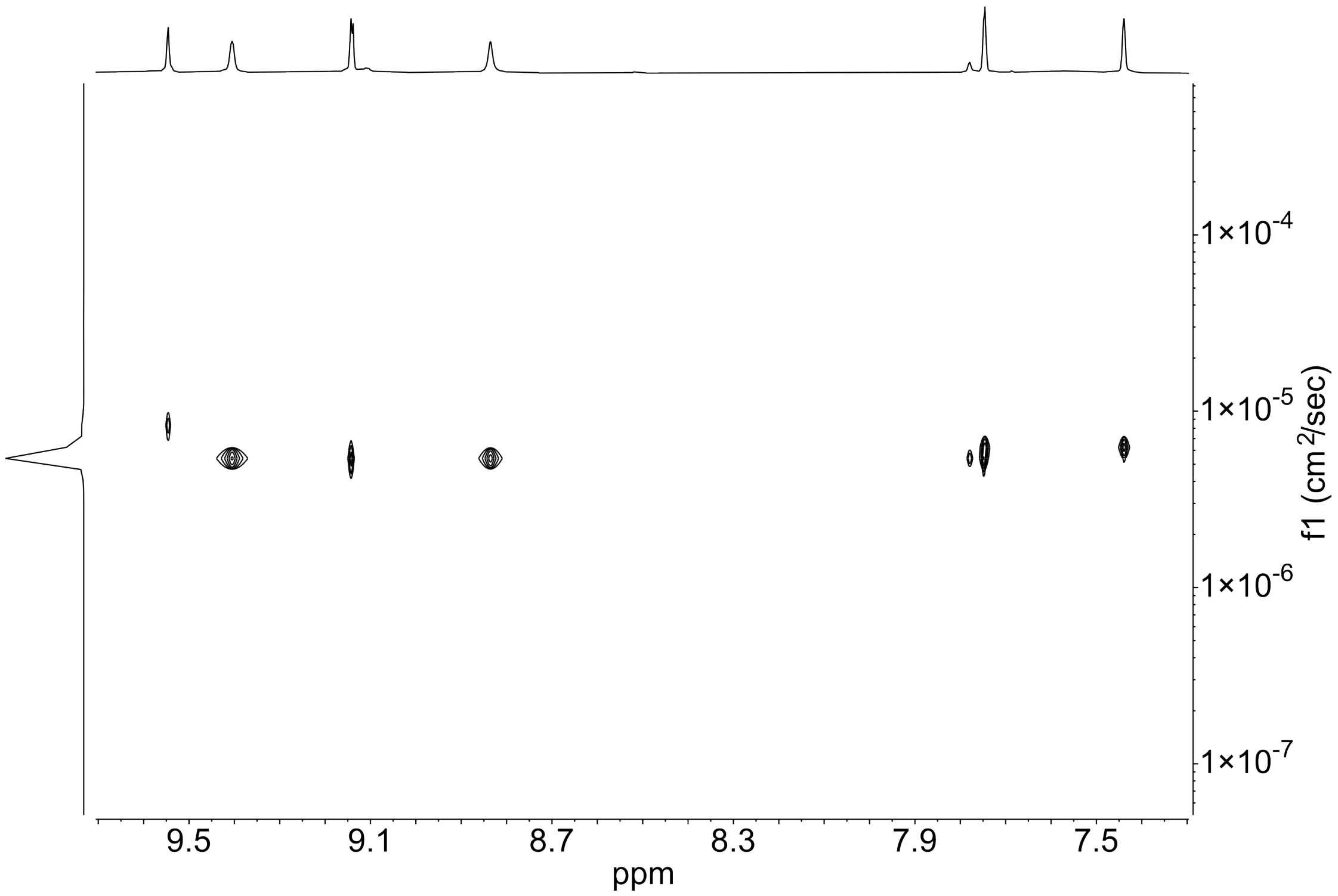

Figure S43. DOSY NMR spectrum (600 MHz, CD_3NO_2) of $[Pt_2Pd_4(\mathbf{L2})_8Cl_8](BF_4)_4$ (D = 5.30 x 10^{-6} cm^2 s^{-1}).

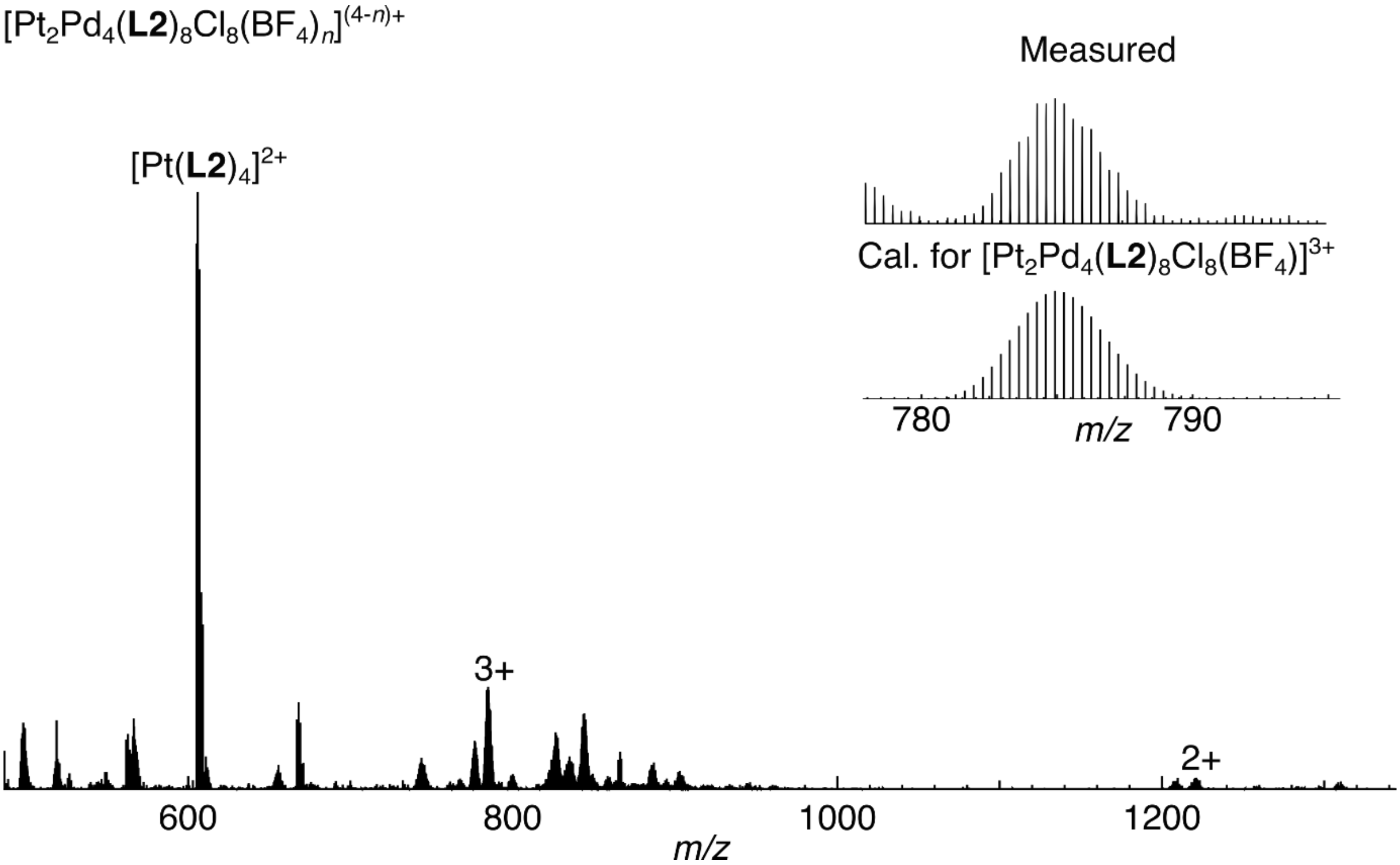

Figure S44. High-resolution ESI mass spectrum of $[Pt_2Pd_4(\mathbf{L2})_8Cl_8(BF_4)_n]^{(4-n)+}$.

3.6 Synthesis of $[Pt_2Pd_4(\mathbf{L3})_8Cl_8](BF_4)_4$

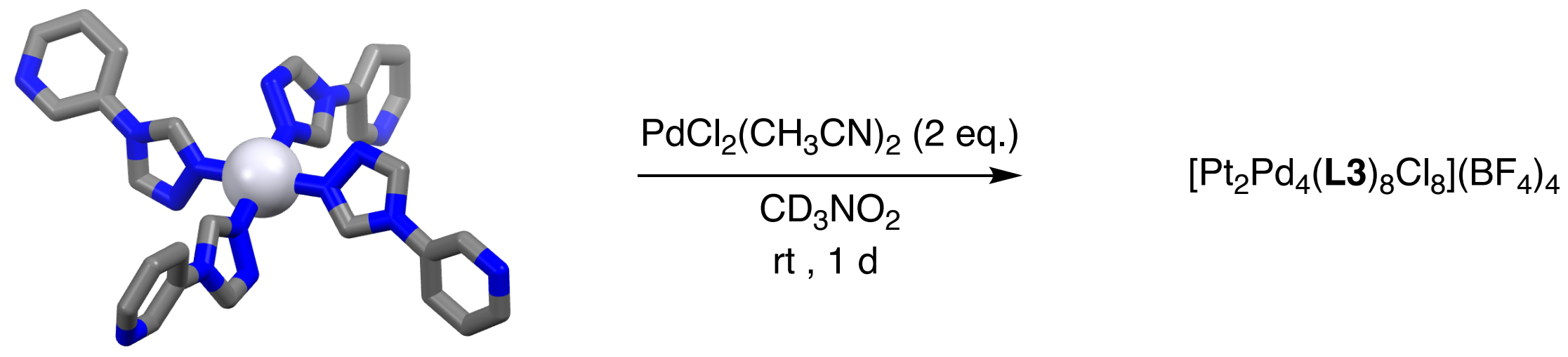

$[Pt(\mathbf{L3})_4](BF_4)_2$ (1.0 eq., 0.8 μmol, 200 μL of a 4 mM stock solution in CD_3NO_2), 320 μL CD_3NO_2, and $PdCl_2(CH_3CN)_2$ (2.0 eq., 1.6 μmol, 80 μL of a 20 mM stock solution in CD_3NO_2) were added to an NMR tube and equilibrated at RT for 1 d to yield cage $[Pt_2Pd_4(\mathbf{L3})_8Cl_8](BF_4)_4$. ^1H (400 MHz, CD_3NO_2) δ 9.54 (s, 8H), 9.17 (d, J = 2.4 Hz, 8H), 9.03 – 8.99 (m, 8H), 8.85 (s, 8H), 8.28 (d, J = 9.3 Hz, 8H), 7.81 (dd, J = 8.6, 5.7 Hz, 8H).

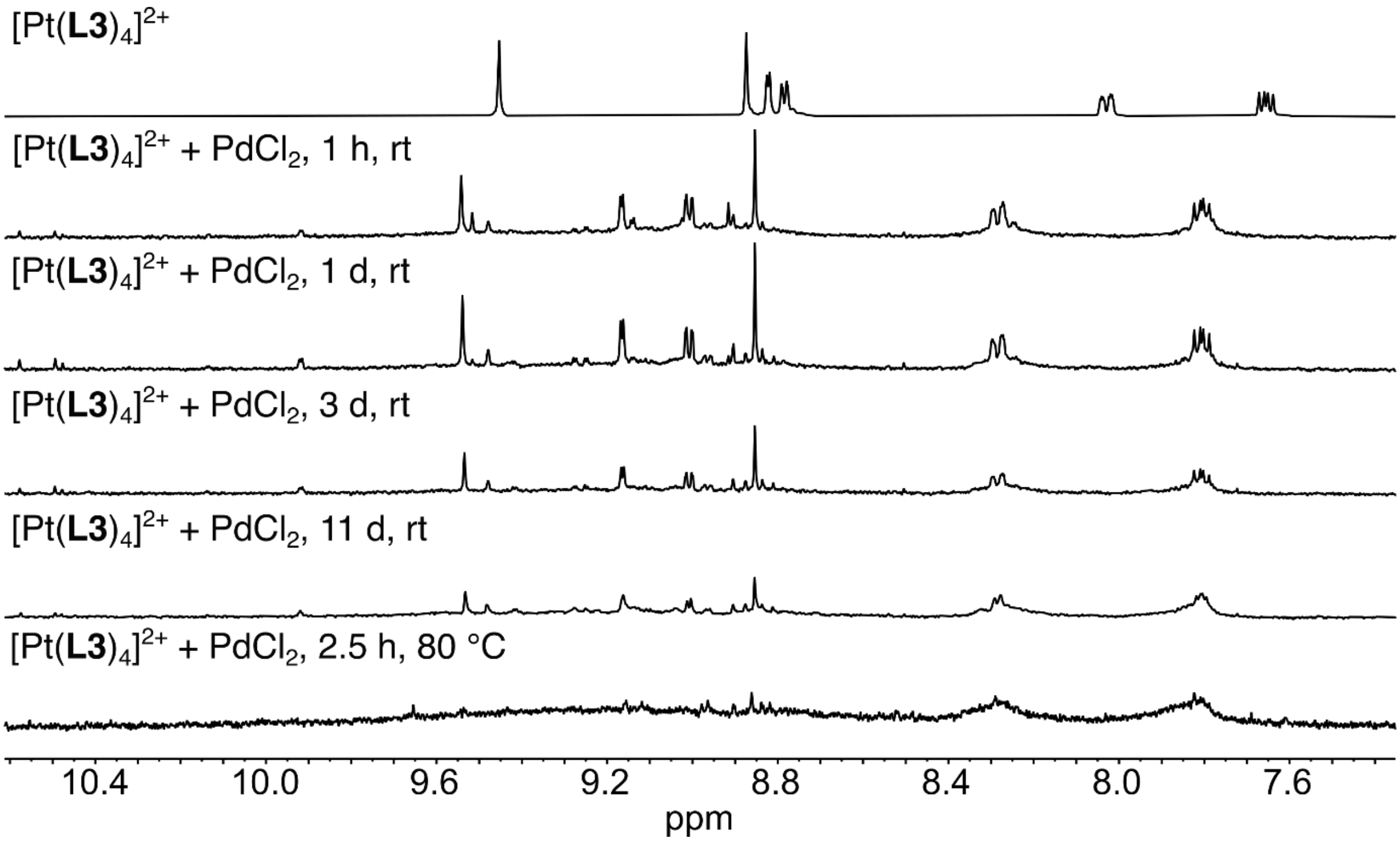

Figure S45. ^1H NMR spectrum (400 MHz (600 MHz for 11 d), CD_3NO_2) of $[Pt_2Pd_4(\mathbf{L3})_8Cl_8](BF_4)_4$.

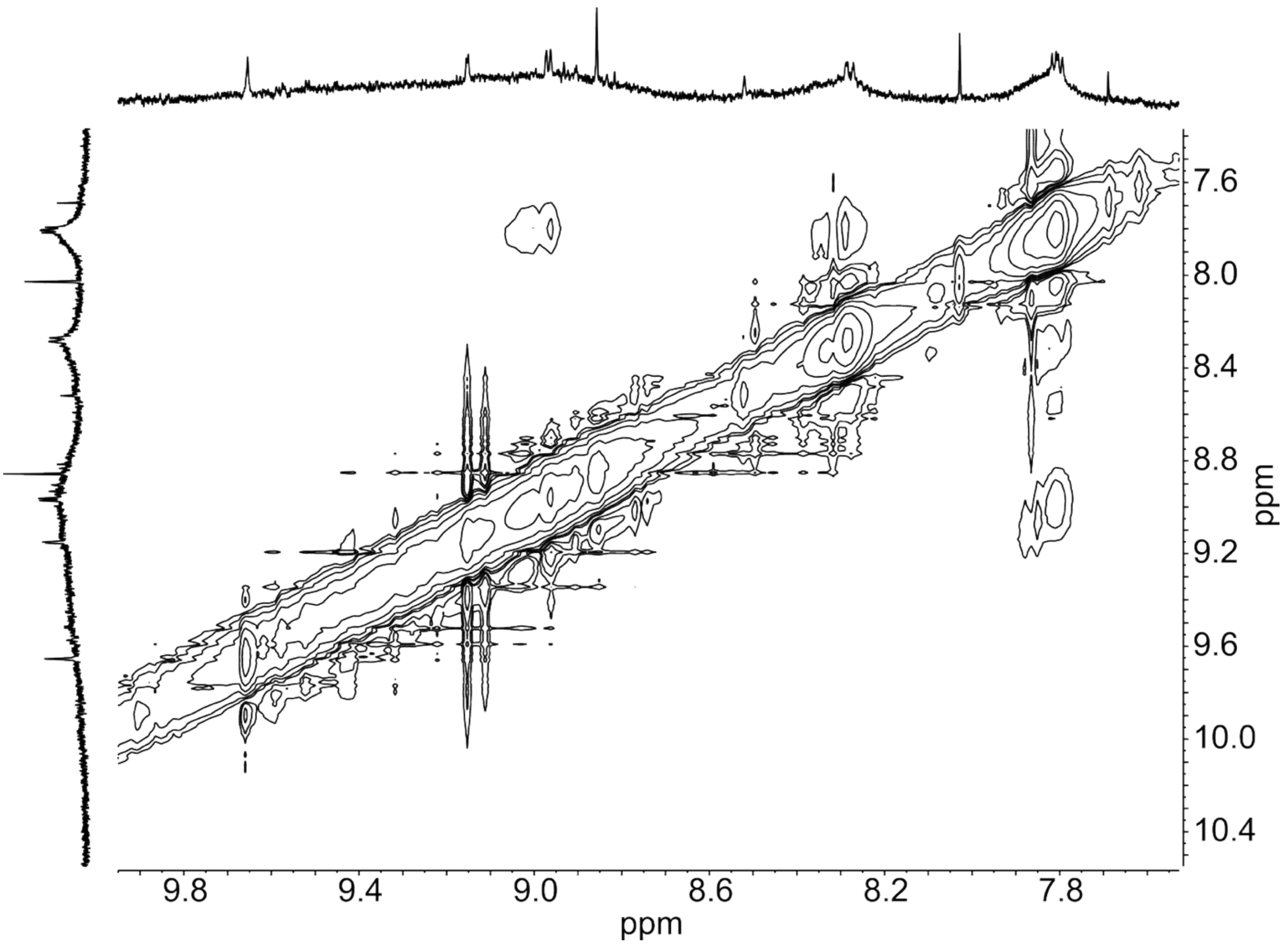

Figure S46. ^1H-^1H NOESY NMR spectrum (600 MHz, CD_3NO_2) of $[Pt_2Pd_4(\mathbf{L3})_8Cl_8](BF_4)_4$.

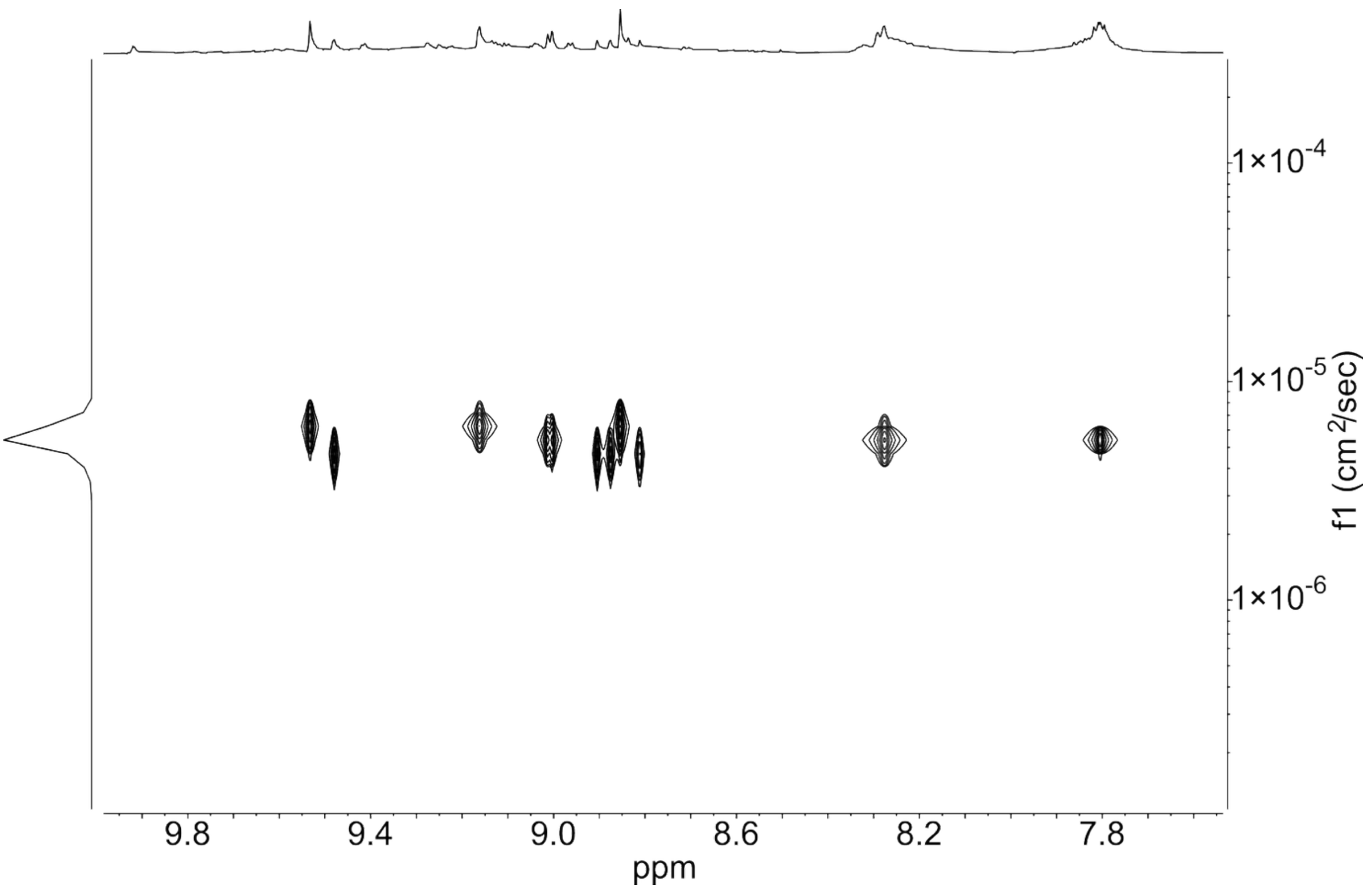

Figure S47. DOSY NMR spectrum (600 MHz, CD_3NO_2) of $[Pt_2Pd_4(\mathbf{L3})_8Cl_8](BF_4)_4$ (D = 5.40 x 10^{-6} cm^2 s^{-1}).

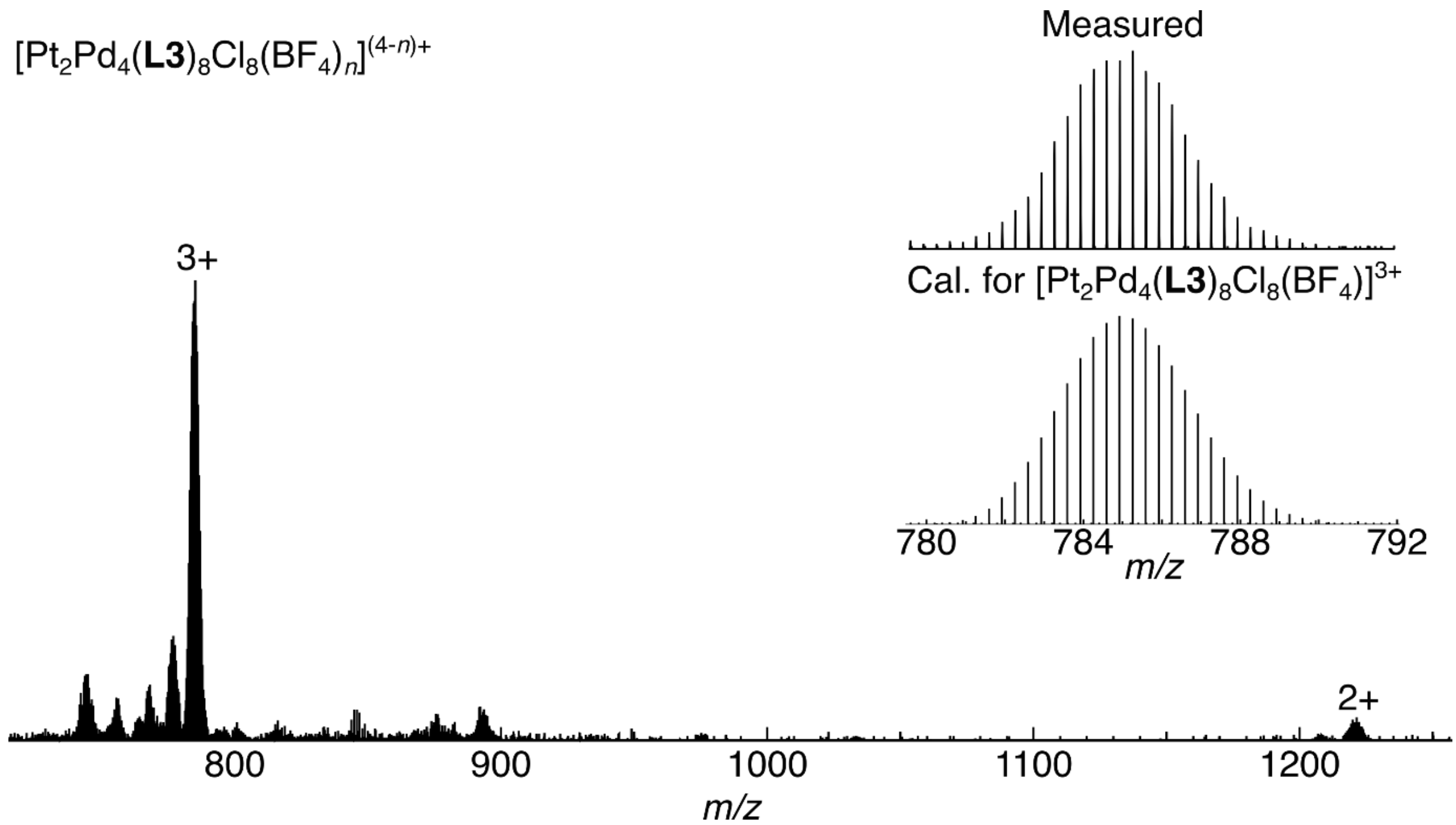

Figure S48. High-resolution ESI mass spectrum of $[Pt_2Pd_4(\mathbf{L3})_8Cl_8(BF_4)_n]^{(4-n)+}$.

4 Cyclic Ion Mobility-Mass spectrometry (cIM-MS)

Sample preparation: Samples were prepared at 3.3 µM in LC-MS grade acetonitrile (≥99.9% purity, Sigma-Aldrich, Saint-Quentin-Fallavier, France).

CIMS-MS Analysis

The cIM-MS analyses were performed on a SELECT SERIES Cyclic Ion Mobility Mass Spectrometer (Waters, Manchester, UK), controlled via MassLynx software.[10] Samples were directly infused at 5 µL/min using a syringe pump (Harvard Apparatus, MA, USA). The ESI source was operated in positive polarity mode with the following parameters: capillary potential, 1.8 kV; source offset, 10 V; nebulizer gas flow, 6 bar; cone gas flow, 0 L/h; desolvation gas flow, 800 L/h; desolvation temperature, 280°C; source temperature, 100°C. All experiments were performed in the 50-2000 *m/z* range during 1 min. The system was mass-calibrated using sodium iodide in V mode TOF resolution prior to analysis. The closed-loop traveling wave IM separator (ring path length: 98 cm) was operated orthogonally to the main ion optical axis, with a traveling wave velocity of 325 m/s and a wave height of 15 V. After screening a large set of TW settings couple, such values provided the best balance between the energetical impact on the molecular assemblies and resolution. The number of passes (n) was optimized for each ion by incrementally increasing the pass count from 1 to 4 (step 1), then by steps of 4, until optimal separation was achieved, while preserving an acceptable signal without ion overtaking. Nitrogen (≥99.99% purity) was used as the collision gas within the ring at a flow rate of 10 mL/min. The IM-MS spectra and resulting Arrival time distribution (ATD) were acquired with 3 pushes per bin and 200 bins. Nitrogen was used as the collision gas within the ring. To minimize as much as possible the internal energy of the non-covalent assemblies, while ensuring good transmission of ions, Nitrogen at a flowrate of 10 ml/min was used gas Collison 1 prior IMS cell (ion cooling), and trap and transfer collision energy set at 2 and 1 V, respectively.

Data Processing and CCS determination.

The raw data were processed using MassLynx.[S4] Extracted ATDs were converted to csv files and used as input for data analysis. The centroids of mobility features in each ATD were determined through Gaussian fitting using nonlinear least square regression and the 'nls' function in the standard R package.[S5] These centroids, representing the peak arrival time of each mobility feature at different separation times, were then used to determine the perturbed periodic drift time. Then, calibrating multipass collision cross section (CCS) measurements with cIMS was performed using the *Multipass CCS Refiner.*[S6]

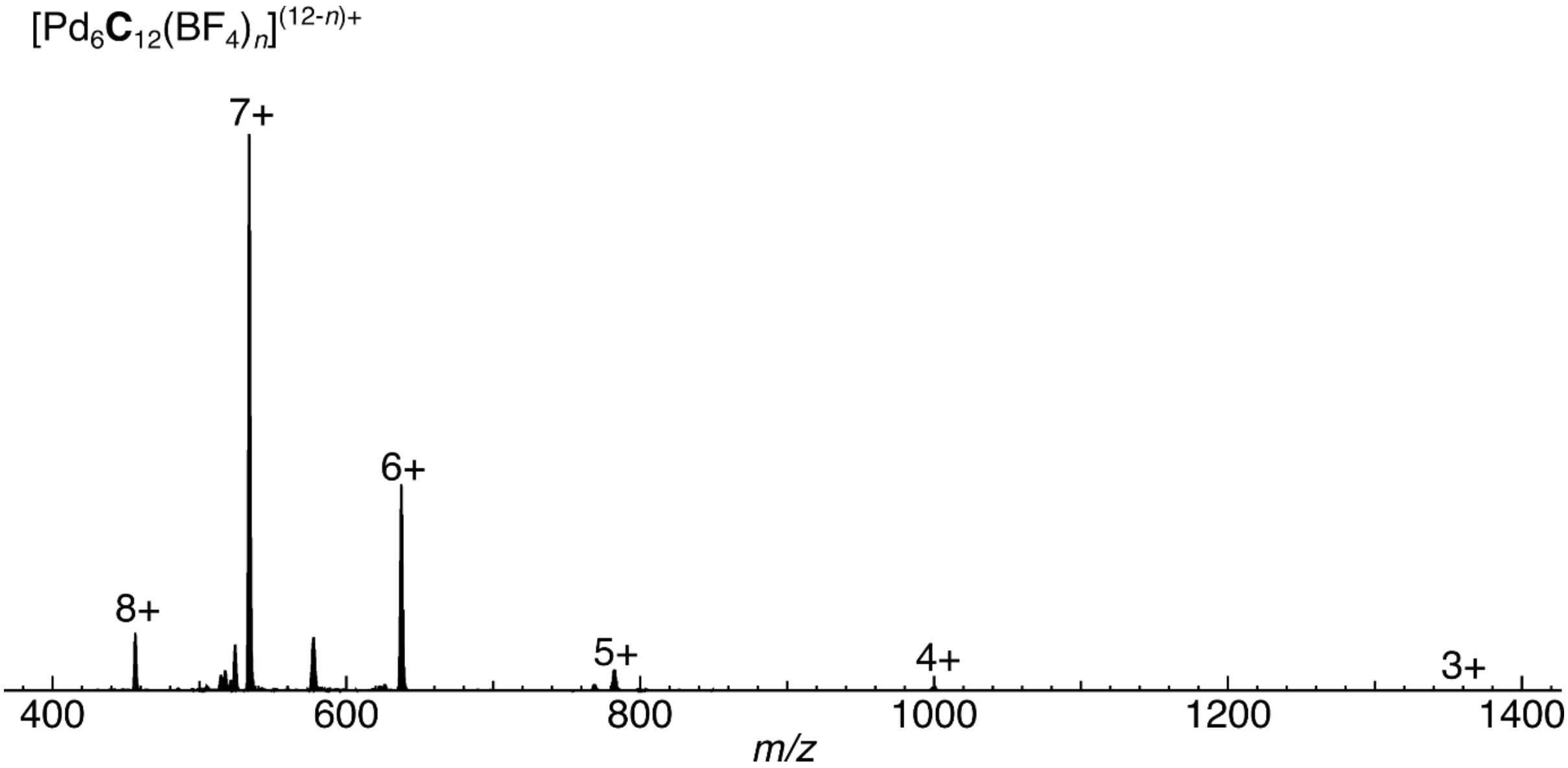

Figure S49. High-resolution ESI mass spectrum of $[Pd_6\mathbf{C}_{12}(BF_4)_n]^{(12-n)+}$.

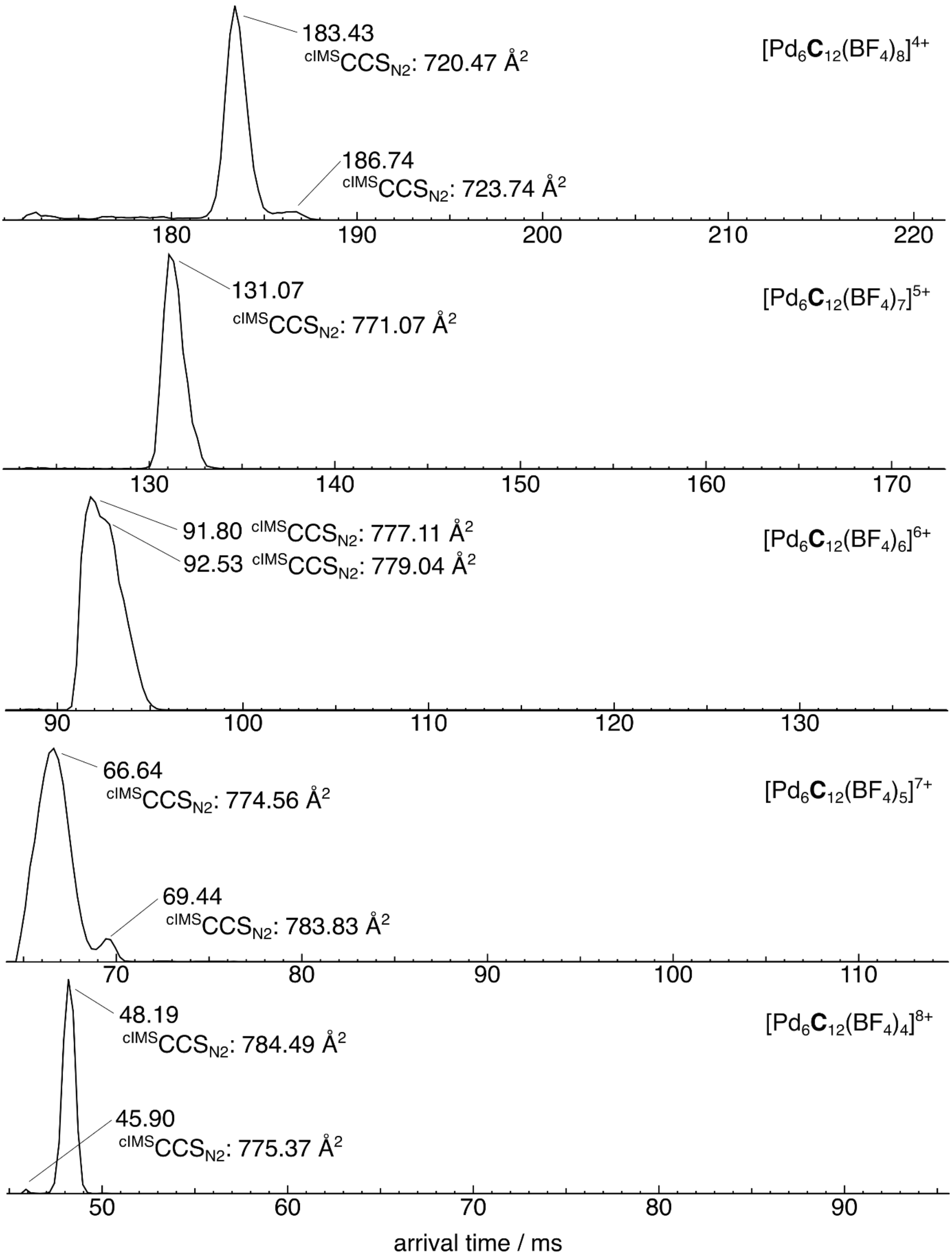

Figure S50. cIM-MS mobilograms of $[Pd_6\mathbf{C}_{12}(BF_4)_n]^{(12-n)+}$ for n = 4–8 after 12 passes.

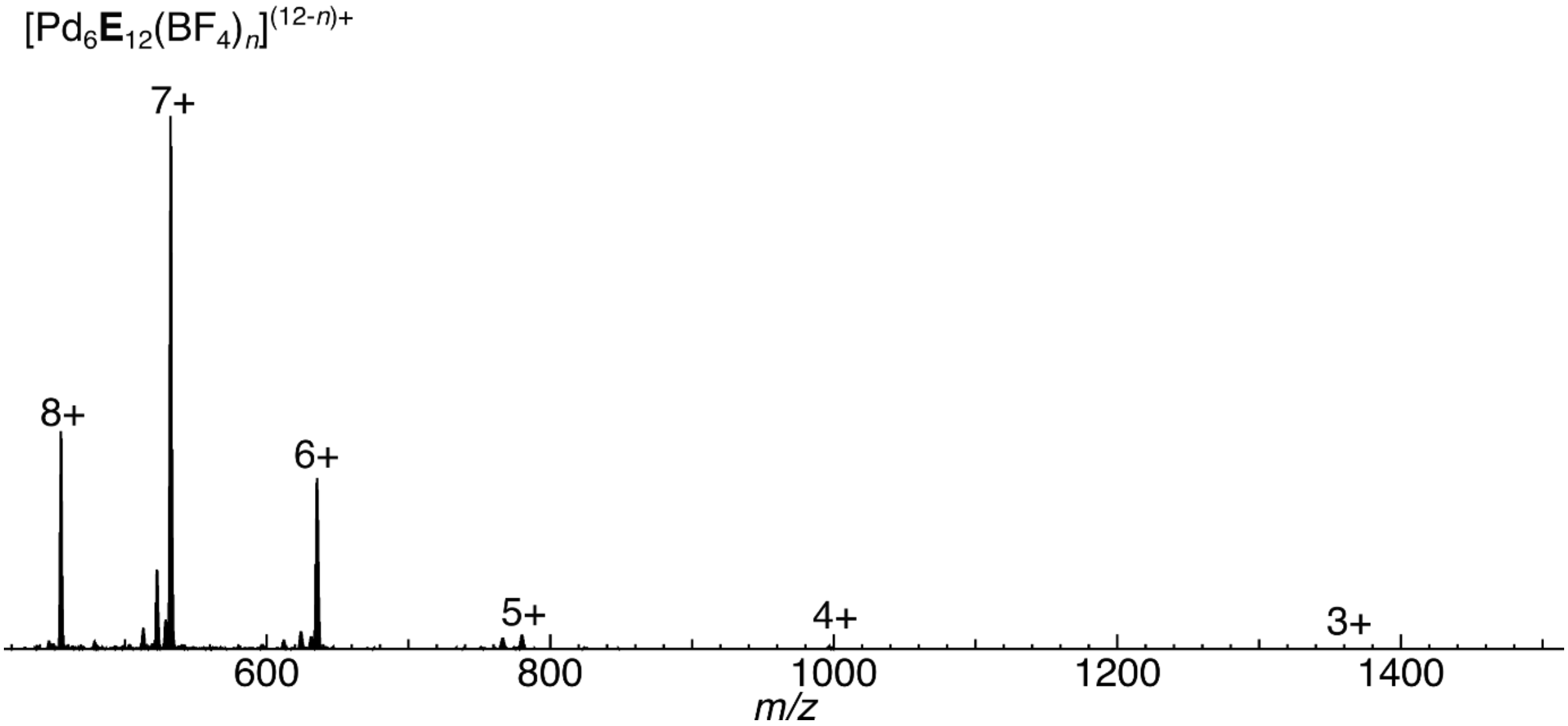

Figure S51. High-resolution ESI mass spectrum of $[Pd_6\mathbf{E}_{12}(BF_4)_n]^{(12-n)+}$.

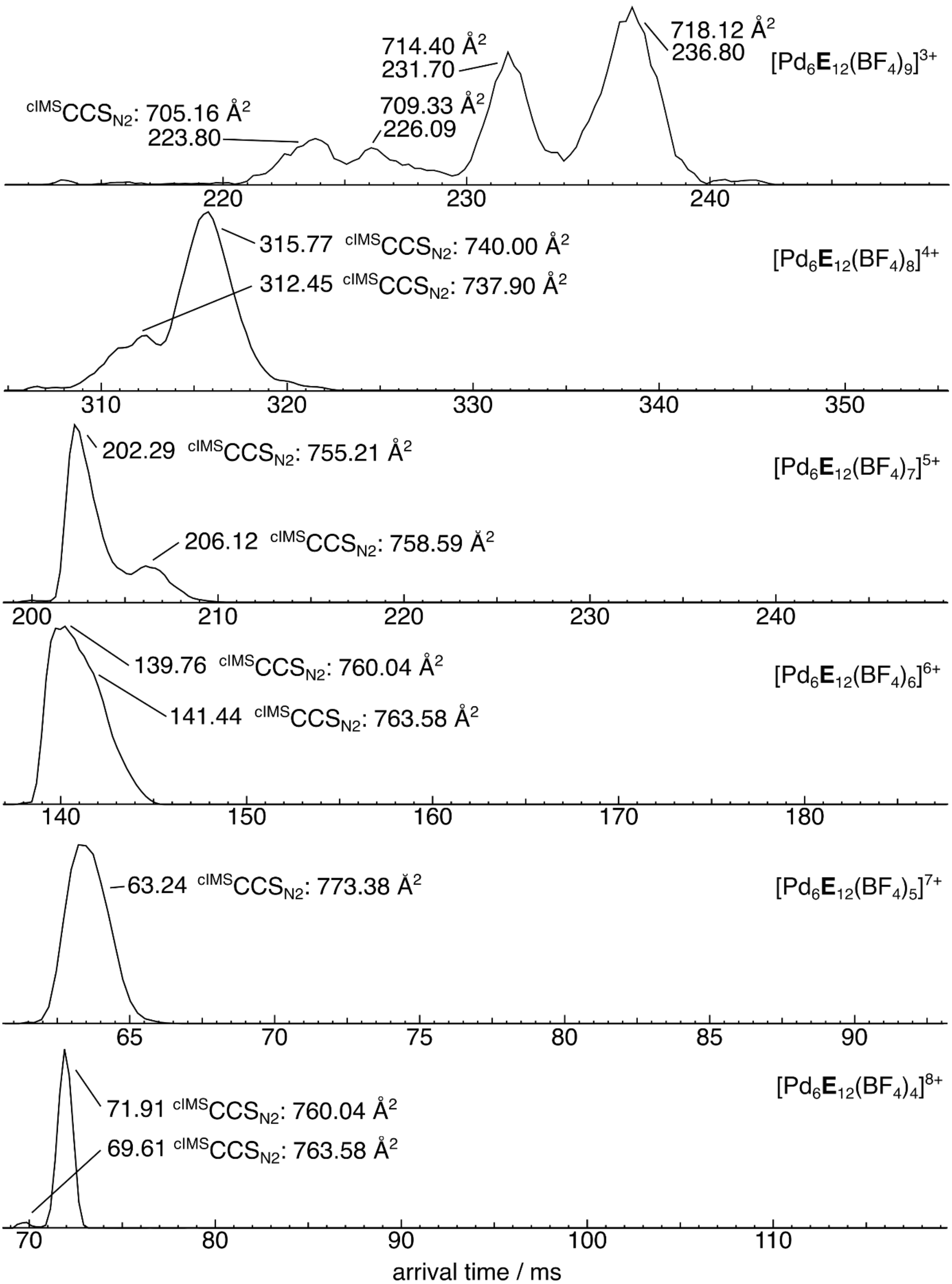

Figure S52. cIM-MS mobilograms of $[Pd_6\mathbf{E}_{12}(BF_4)_n]^{(12-n)+}$ for n = 4–9 after 20, 12, 20, 20, 20 and 8 passes, respectively.

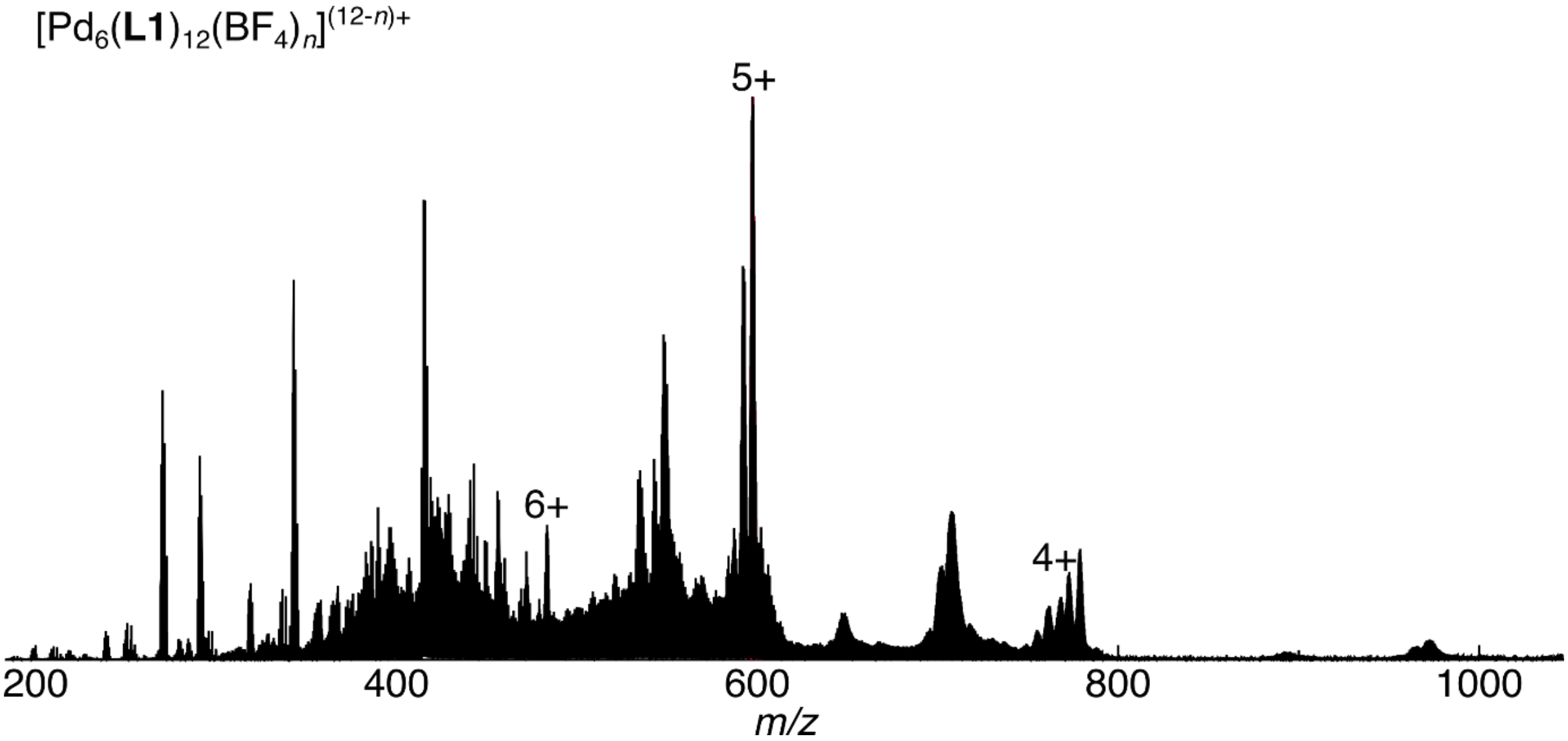

Figure S53. High-resolution ESI mass spectrum of $[Pd_6(\mathbf{L1})_{12}(BF_4)_n]^{(12-n)+}$.

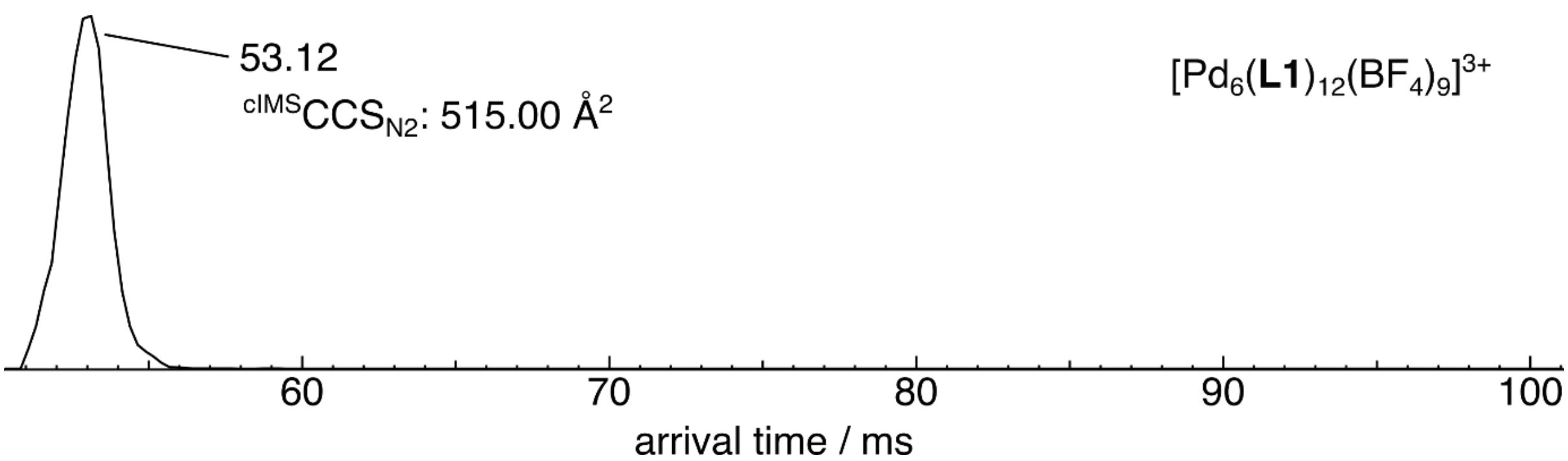

Figure S54. cIM-MS mobilograms of $[Pd_6(\mathbf{L1})_{12}(BF_4)_n]^{(12-n)+}$ for n = 9 after 12 passes.

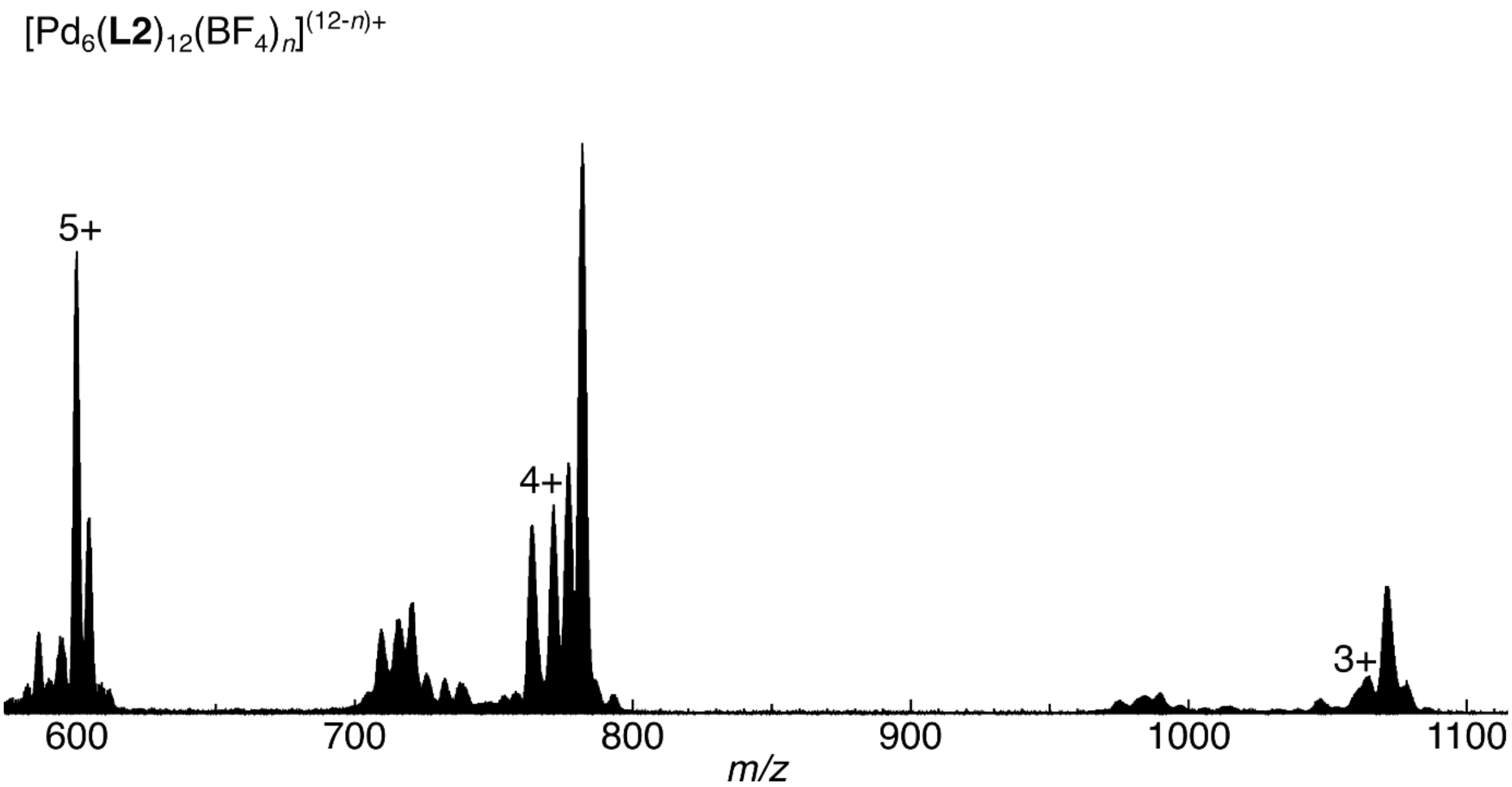

Figure S55. High-resolution ESI mass spectrum of $[Pd_6(\mathbf{L2})_{12}(BF_4)_n]^{(12-n)+}$.

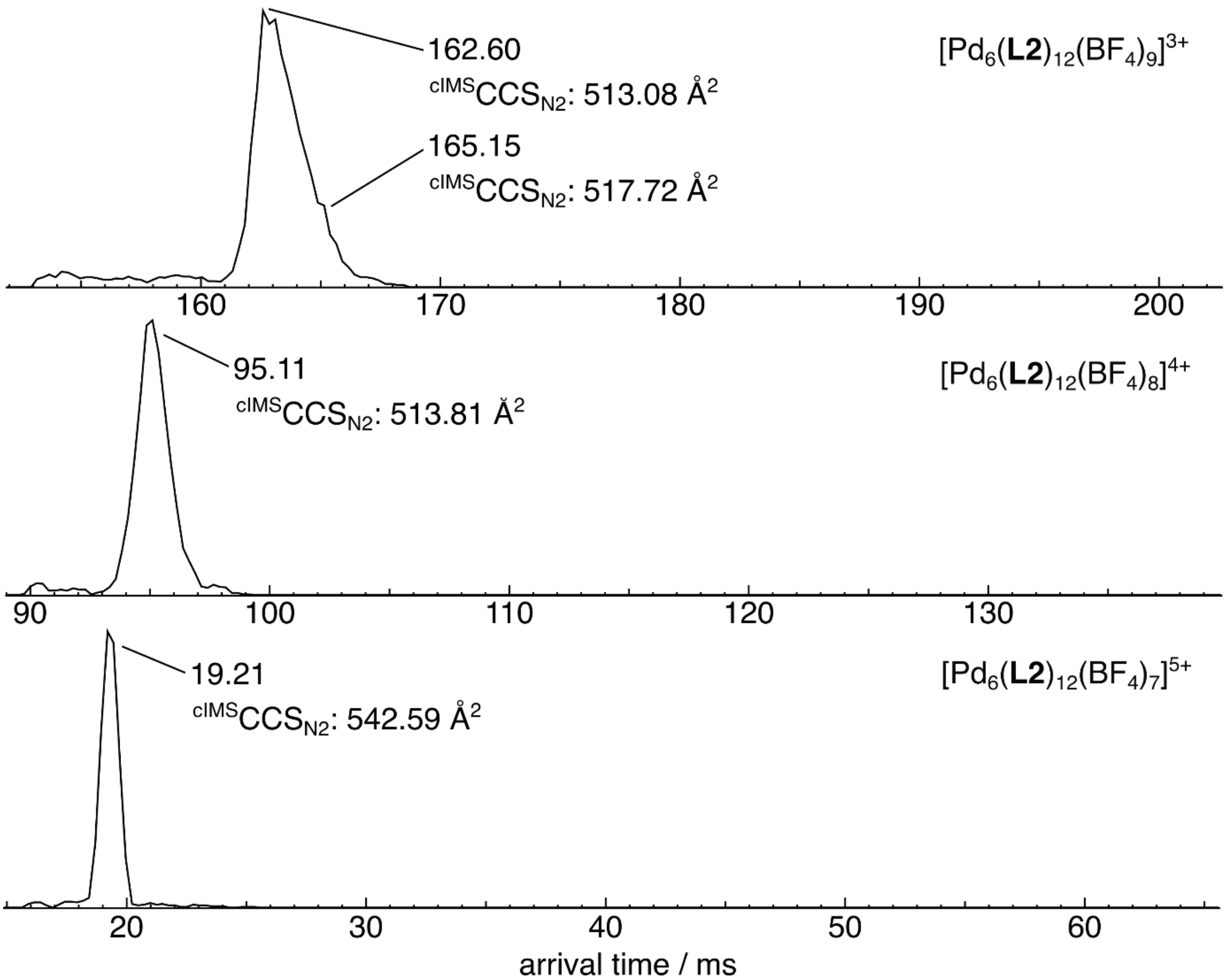

Figure S56. cIM-MS mobilograms of $[Pd_6(\mathbf{L2})_{12}(BF_4)_n]^{(12-n)+}$ for *n* = 7–9 after 2, 12 and 12 passes, respectively.

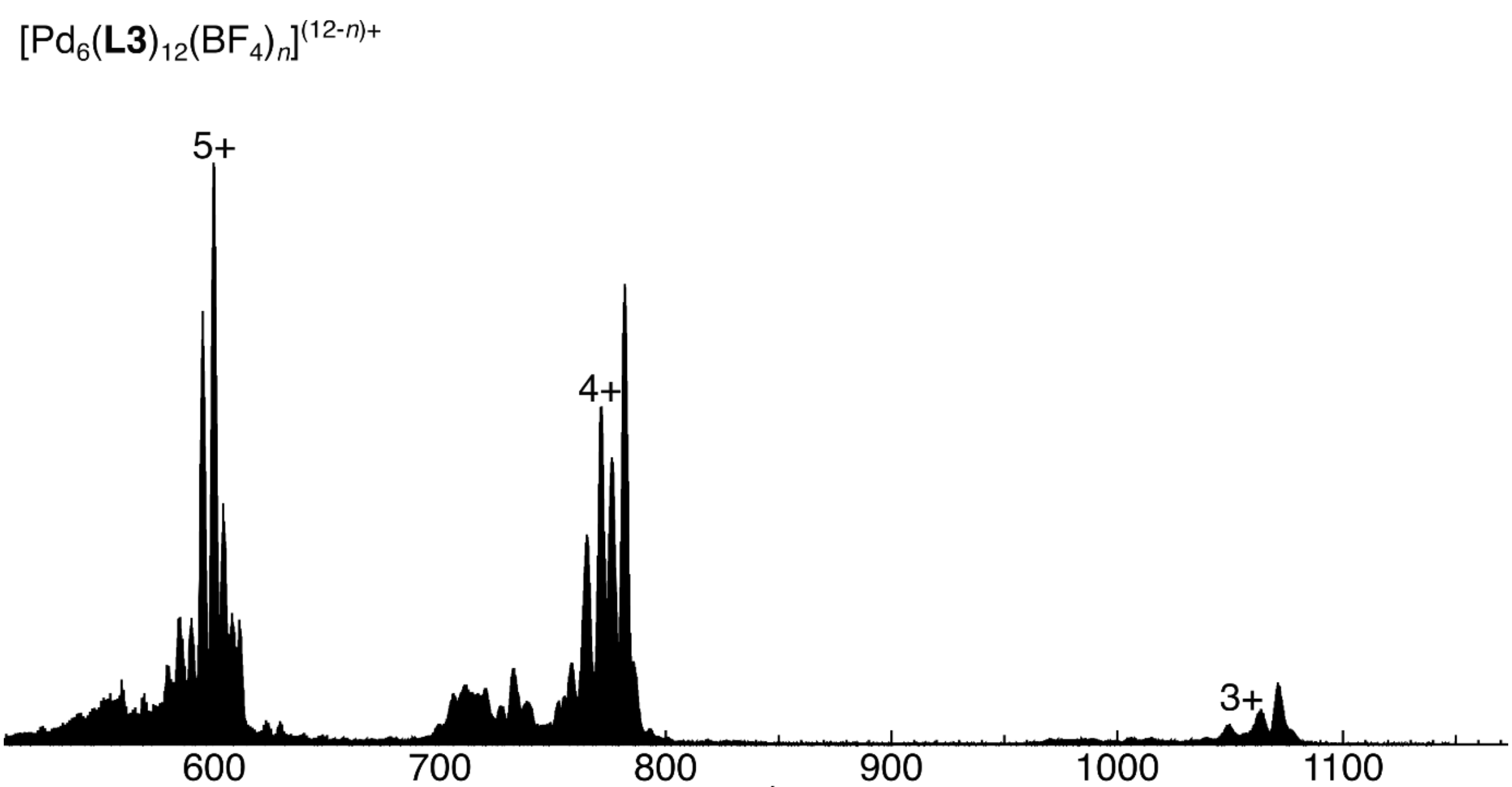

Figure S57. High-resolution ESI mass spectrum of $[Pd_6(\mathbf{L3})_{12}(BF_4)_n]^{(12-n)+}$.

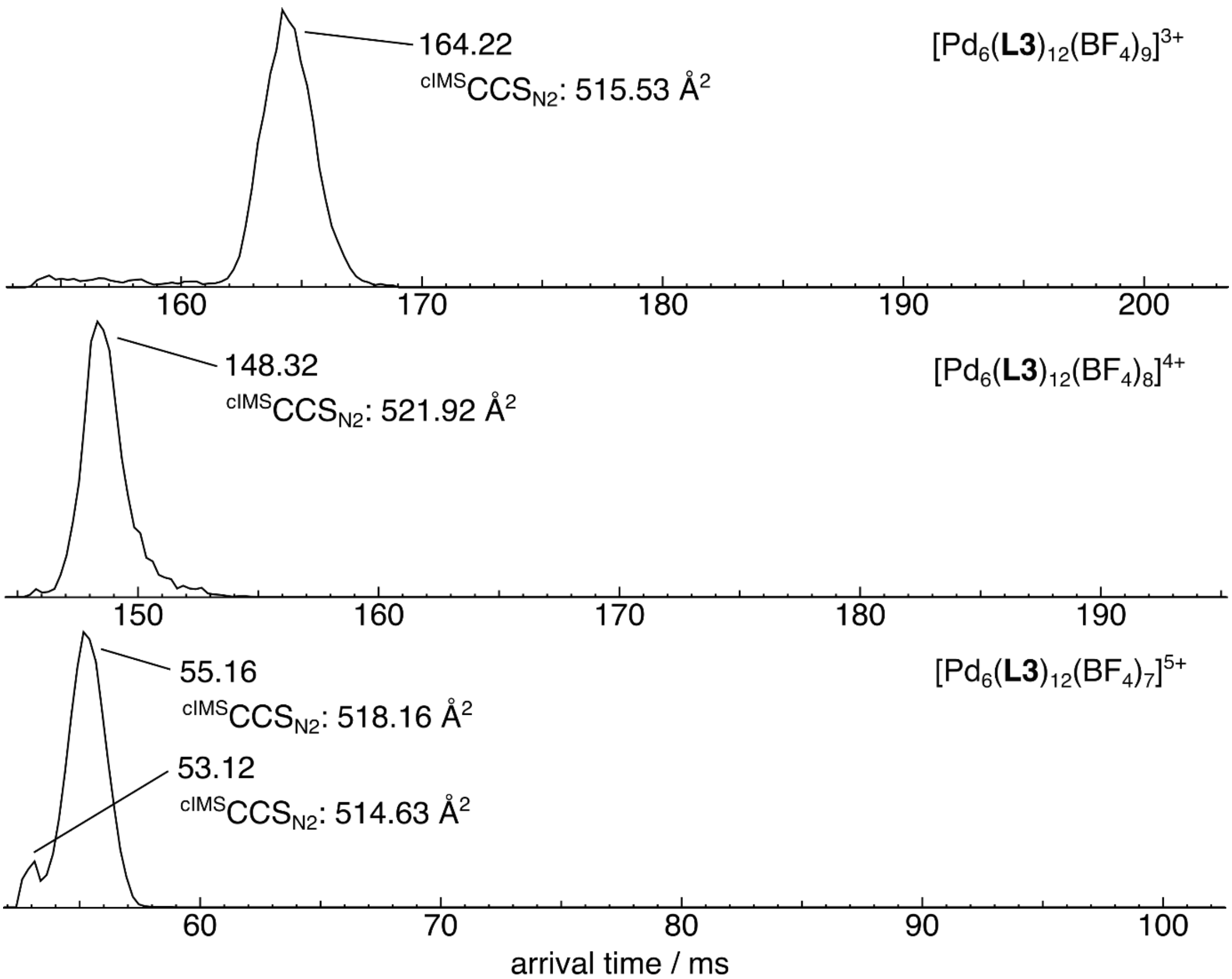

Figure S58. cIM-MS mobilograms of $[Pd_6(\mathbf{L3})_{12}(BF_4)_n]^{(12-n)+}$ for n = 7–9 after 12, 20 and 12 passes, respectively.

5 Crystallographic analyses

Single crystals of [Pt(**L1**)$_4$](BF$_4$)$_2$ (20 mM, 1:1 DMSO-d$_6$:CD$_3$CN), [Pt(**L2**)$_4$](BF$_4$)$_2$ (20 mM, 1:1 DMSO-d$_6$:CD$_3$CN), and [Pt(**L3**)$_4$](BF$_4$)$_2$ (20 mM, 1:3 DMSO-d$_6$:CD$_3$CN) were obtained by layering toluene after 3 days. Single crystals of [Pd$_6$(**L1**)$_{12}$](BF$_4$)$_{12}$ were obtained by slow (4 weeks) vapor diffusion of a Et$_2$O : THF mixture (1:1) into a 0.33 mM cage solution in CD$_3$CN. Single crystals of [Pd$_6$(**L1**)$_{12}$](OTf)$_{12}$ were obtained by slow (8 weeks) vapor diffusion of ^{i}Pr$_2$O into a 0.33 mM cage solution in CD$_3$CN. Single crystals of [Pd$_6$(**L2**)$_{12}$](BF$_4$)$_{12}$ were obtained by slow (2 weeks) vapor diffusion of ^{i}Pr$_2$O into a 0.33 mM cage solution in CD$_3$CN. Single crystals of [Pd$_6$(**L3**)$_{12}$](BF$_4$)$_{12}$ were obtained by slow (4 weeks) vapor diffusion of a Et$_2$O: THF mixture (1:1) into a 0.33 mM cage solution in CD$_3$CN. Single crystals of [Pt$_2$Pd$_4$(**L1**)$_8$Cl$_8$](BF$_4$)$_4$ were obtained by slow (2 weeks) vapor diffusion of ^{i}Pr$_2$O into a 0.66 mM cage solution in CD$_3$CN. Single crystals of [Pt$_2$Pd$_4$(**L2**)$_8$Cl$_8$](BF$_4$)$_4$ were obtained by slow (3 weeks) vapor diffusion of ^{i}Pr$_2$O into a 0.66 mM cage solution in CD$_3$NO$_2$.

Details for the analysis of [Pt(L1)$_4$](BF$_4$)$_2$:

A colorless needle-shaped crystal with dimensions 0.14 × 0.03 × 0.02 mm^3 was mounted. Data were collected using a XtaLAB Synergy R, DW system, HyPix-Arc 150 diffractometer operating at T = 140.00(10) K. Data were measured using ω scans with Cu K$_\alpha$ radiation. The diffraction pattern was indexed and the total number of runs and images was based on the strategy calculation from the program CrysAlis^{Pro}.[S7] The maximum resolution achieved was Θ = 74.127°. The unit cell was refined using CrysAlis^{Pro} on 5364 reflections, 34% of the observed reflections.

Data reduction, scaling and absorption corrections were performed using CrysAlis^{Pro}. The final completeness is 99.50 % out to 74.127° in Θ. A Gaussian absorption correction was performed using CrysAlis^{Pro}. Numerical absorption correction based on Gaussian integration over a multifaceted crystal model. Empirical absorption correction using spherical harmonics as implemented in SCALE3 ABSPACK scaling algorithm. The absorption coefficient μ of this material is 7.146 mm^{-1} at this wavelength (λ = 1.54184Å) and the minimum and maximum transmissions are 0.498 and 0.962. The structure was solved in the space group $P\bar{1}$ (# 2) by ShelXT[S8] using dual methods, Olex2[S9] as the graphical interface and was refined by full matrix least squares minimization on $|F|^2$ using of ShelXL.[S10] All non-hydrogen atoms were refined anisotropically. Hydrogen atom positions were calculated geometrically and refined using the riding model. The value of Z' is 0.5. This means that only half of the formula unit is present in the asymmetric unit, with the other half consisting of symmetry equivalent atoms. The moiety formula is C$_{32}$H$_{28}$N$_{12}$Pt, 2(BF$_4$), C$_7$H$_8$.

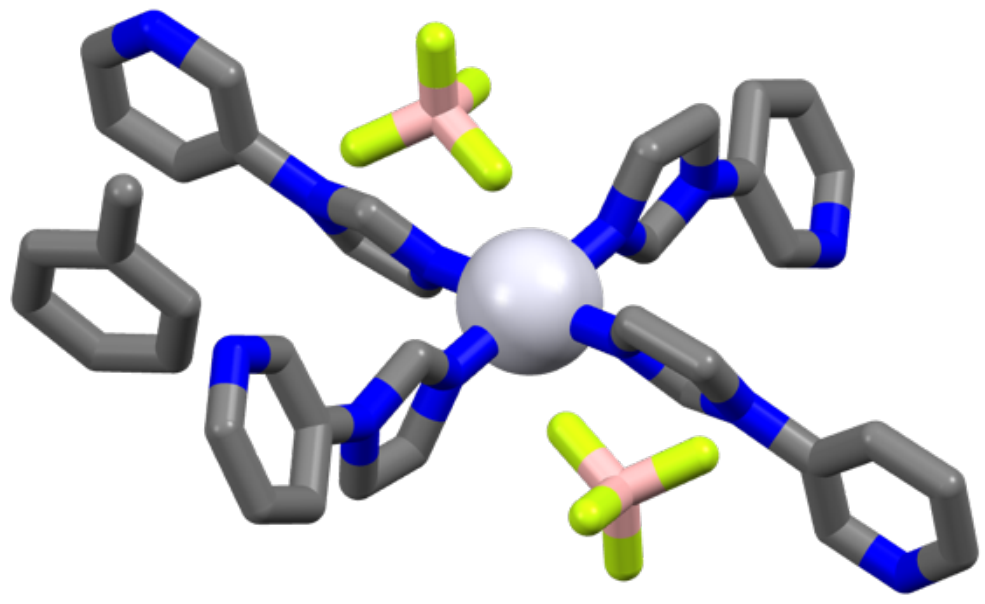

Figure S59. Structure of [Pt(**L1**)$_4$](BF$_4$)$_2$ as determined by single-crystal XRD with two BF_4^- anions and one toluene molecule. Hydrogen atoms are omitted for clarity.

Details for the analysis of [Pt(L2)$_4$](BF$_4$)$_2$:

A colorless prism-shaped crystal with dimensions 0.16 × 0.12 × 0.05 mm^3 was mounted. Data were collected using a XtaLAB Synergy R, DW system, HyPix-Arc 150 diffractometer operating at T = 140.00(10) K. Data were measured using ω scans with Mo K_α radiation. The diffraction pattern was indexed and the total number of runs and images was based on the strategy calculation from the program CrysAlis^{Pro}.[S7] The maximum resolution achieved was Θ = 30.507°. The unit cell was refined using CrysAlis^{Pro} on 19222 reflections, 45% of the observed reflections.

Data reduction, scaling and absorption corrections were performed using CrysAlis^{Pro}. The final completeness is 99.80 % out to 30.507° in Θ. A Gaussian absorption correction was performed using CrysAlis^{Pro}. Numerical absorption correction based on Gaussian integration over a multifaceted crystal model. Empirical absorption correction using spherical harmonics as implemented in SCALE3 ABSPACK scaling algorithm. The absorption coefficient μ of this material is 3.452 mm^{-1} at this wavelength (λ = 0.71073 Å) and the minimum and maximum transmissions are 0.683 and 1.000. The structure was solved in the space group $P2_1/c$ (# 14) by ShelXT[S8] using dual methods, Olex2[S9] as the graphical interface and was refined by full matrix least squares minimization on $|F|^2$ using ShelXL[S10] All non-hydrogen atoms were refined anisotropically. Hydrogen atom positions were calculated geometrically and refined using the riding model. The value of Z' is 0.5. This means that only half of the formula unit is present in the asymmetric unit, with the other half consisting of symmetry equivalent atoms. The moiety formula is $C_{28}H_{24}N_{16}Pt$, 2(BF_4), C_7H_8.

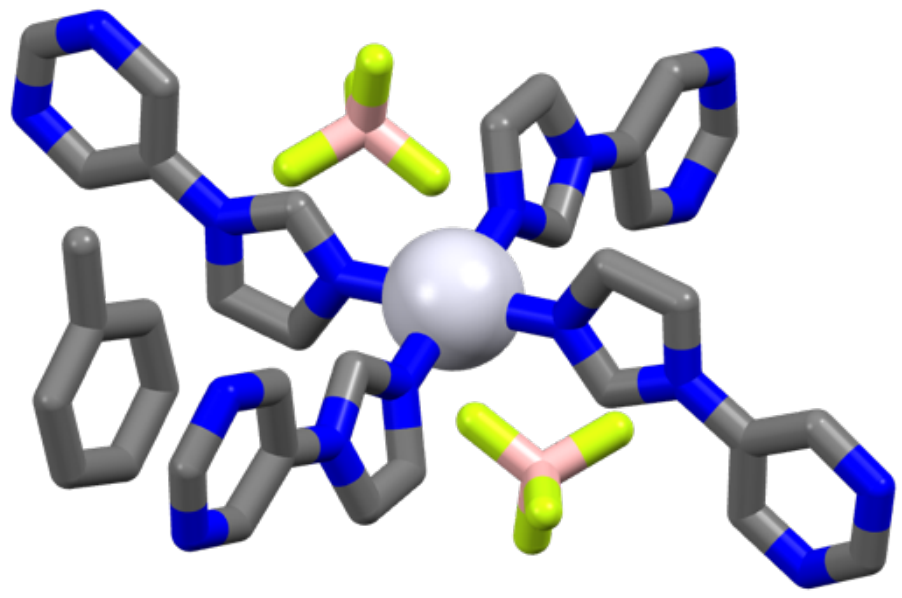

Figure S60. Structure of $[Pt(\mathbf{L2})_4](BF_4)_2$ as determined by single-crystal XRD with two BF_4^- anions and one toluene molecule. Hydrogen atoms are omitted for clarity.

Details for the analysis of $[Pt(L3)_4](BF_4)_2$:

A colorless prism-shaped crystal with dimensions 0.29 × 0.11 × 0.05 mm^3 was mounted. Data were collected using a XtaLAB Synergy R, DW system, HyPix-Arc 150 diffractometer operating at T = 140.00(10) K. Data were measured using ω scans with Mo K_α radiation. The diffraction pattern was indexed and the total number of runs and images was based on the strategy calculation from the program CrysAlis^{Pro}.[S7] The maximum resolution achieved was Θ = 36.316°. The unit cell was refined using CrysAlis^{Pro} on 89762 reflections, 60% of the observed reflections.

Data reduction, scaling and absorption corrections were performed using CrysAlis^{Pro}. The final completeness is 99.90 % out to 36.316° in Θ. A Gaussian absorption correction was performed using CrysAlis^{Pro}. Numerical absorption correction based on Gaussian integration over a multifaceted crystal model. Empirical absorption correction using spherical harmonics as implemented in SCALE3 ABSPACK scaling algorithm. The absorption coefficient μ of this material is 3.380 mm^{-1} at this wavelength (λ = 0.71073 Å) and the minimum and maximum transmissions are 0.503 and 1.000. The structure was solved in the space group $P\bar{1}$ (# 2) by ShelXT[S8] using dual methods, Olex2[S9] as the graphical interface and was refined by full matrix least squares minimization on $|F|^2$ using version 2019/3 of ShelXL.[S10] All non-hydrogen atoms were refined anisotropically. Hydrogen atom positions were calculated geometrically and refined using the riding model. There is a single formula unit in the asymmetric unit, which is represented by the reported sum formula. In other words: Z is 2 and Z' is 1. The moiety formula is $C_{28}H_{24}N_{16}Pt$, $2(BF_4)$, $1.5(C_7H_8)$.

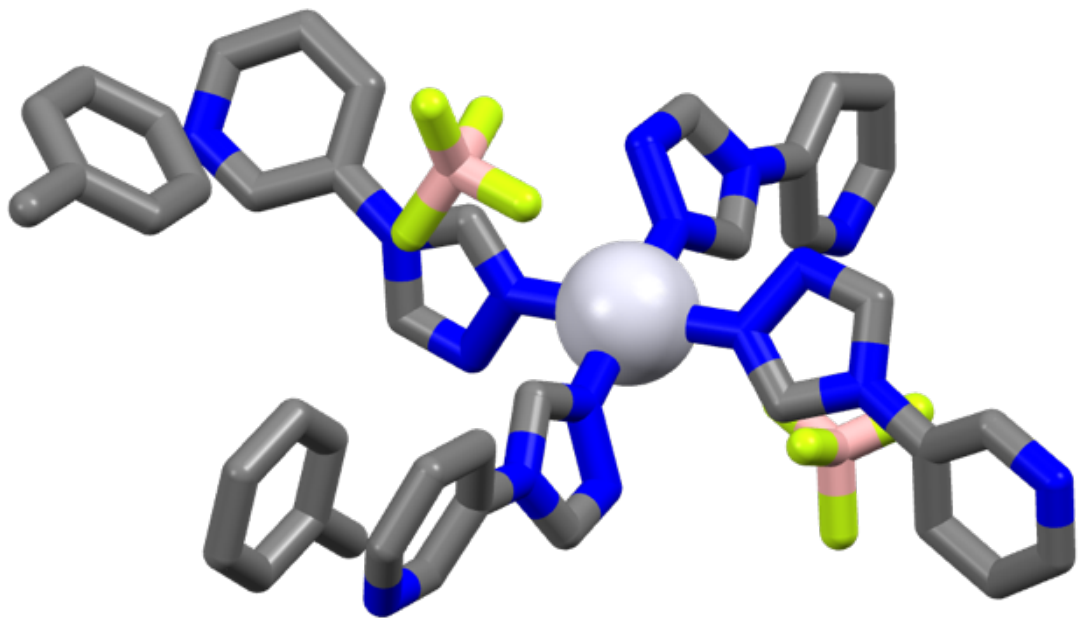

Figure S61. Structure of $[Pt(\mathbf{L3})_4](BF_4)_2$ as determined by single-crystal XRD with two BF_4^- anions and two toluene molecules. Hydrogen atoms are omitted for clarity.

Details for the analysis of $[Pd_6(L1)_{12}](BF_4)_{12}$:

A colorless prism-shaped crystal with dimensions 0.15 × 0.15 × 0.07 mm^3 was mounted. Data were collected using a XtaLAB Synergy R, DW system, HyPix-Arc 150 diffractometer operating at T = 229.99(10) K. Data were measured using ω scans with Cu K_α radiation. The diffraction pattern was indexed and the total number of runs and images was based on the strategy calculation from the program CrysAlis^{Pro}.[S7] The maximum resolution achieved was Θ = 74.890°. The unit cell was refined using CrysAlisPro on 29971 reflections, 45% of the observed reflections.

Data reduction, scaling and absorption corrections were performed using CrysAlisPro. The final completeness is 99.60 % out to 74.890° in Θ. A Gaussian absorption correction was performed using CrysAlisPro. The absorption coefficient μ of this material is 5.769 mm^{-1} at this wavelength (λ = 1.54184Å) and the minimum and maximum transmissions are 0.527 and 1.000.

The structure was solved in the space group $R\bar{3}c$ (# 167) by ShelXT[S8] using dual methods, Olex2[S9] as the graphical interface and was refined by full matrix least squares minimization on $|F|^2$ using ShelXL.[S10] All non-hydrogen atoms were refined anisotropically. Hydrogen atom positions were calculated geometrically and refined using the riding model. A solvent mask was calculated and 991 electrons were found in a volume of 9861 $Å^3$ in 2 voids per unit cell. This is consistent with the presence of 3[BF4] per Asymmetric Unit which account for 738 electrons per unit cell. The value of Z' is 0.166667. The moiety formula is $C_{96}H_{84}N_{36}Pd_6$, 9(BF_4), 3[BF_4].

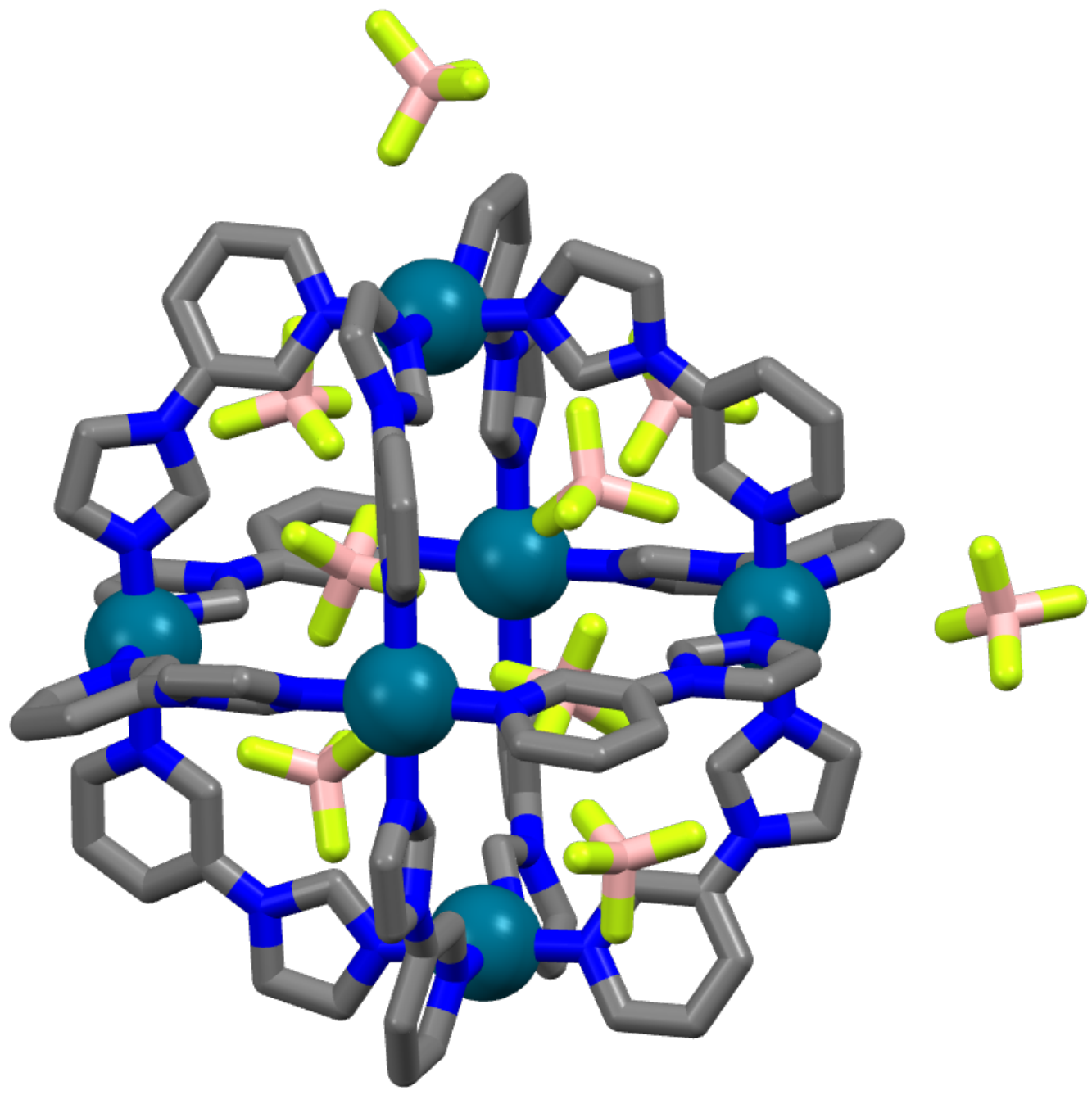

Figure S62. Structure of $[Pd_6(\mathbf{L1})_{12}]^{12+}$ with nine BF_4^- anions as determined by single-crystal XRD. The hydrogen atoms are omitted for clarity.

Details for the analysis of $[Pd_6(L1)_{12}](OTf)_{12}$:

A colorless, irregular-shaped crystal with dimensions 0.09 × 0.05 × 0.03 mm^3 was mounted. Data were collected using a XtaLAB Synergy R, DW system, HyPix-Arc 150 diffractometer operating at T = 140.00(10) K. Data were measured using ω scans with Cu K_α radiation. The diffraction pattern was indexed and the total number of runs and images was based on the strategy calculation from the program CrysAlis^{Pro}.[S7] The maximum resolution achieved was Θ = 39.970°. The unit cell was refined using CrysAlis^{Pro} on 13867 reflections, 46% of the observed reflections.

Data reduction, scaling and absorption corrections were performed using CrysAlis^{Pro}. The final completeness is 99.40 % out to 39.970° in Θ. A Gaussian absorption correction was performed using CrysAlis^{Pro}. Numerical absorption correction based on Gaussian integration over a multifaceted crystal model. Empirical absorption correction using spherical harmonics as implemented in SCALE3 ABSPACK scaling algorithm. The absorption coefficient μ of this material is 6.566 mm^{-1} at this wavelength (λ = 1.54184 Å) and the minimum and maximum transmissions are 0.593 and 0.864.

The structure was solved in the space group $C2/c$ (# 15) by ShelXT[S8] using dual methods, Olex2[S9] as the graphical interface and was refined by full matrix least squares minimization on $|F|^2$ using ShelXL.[S10] All non-hydrogen atoms were refined anisotropically. Hydrogen atom positions were calculated geometrically and refined using the riding model. The value of Z' is 0.5. This means that only half of the formula unit is present in the asymmetric unit, with the

other half consisting of symmetry equivalent atoms. The moiety formula is $C_{96}H_{80}N_{36}Pd_6$, 7(CF_3O_3S), 5[CF_3O_3S], 2[$C_6H_{14}O$].

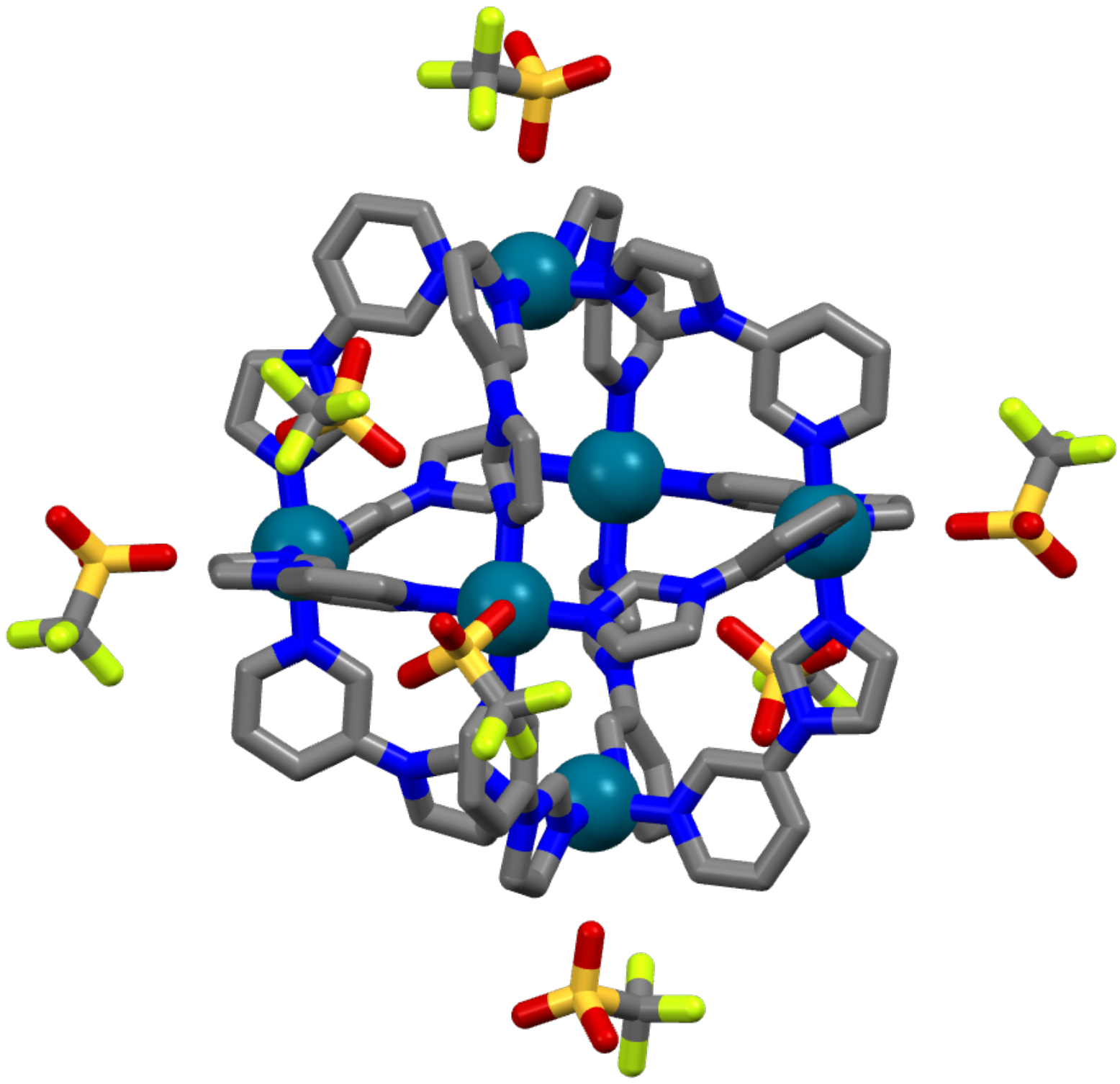

Figure S63. Structure of $[Pd_6(\mathbf{L1})_{12}]^{12+}$ with seven OTf^- anions as determined by single-crystal XRD. The hydrogen atoms are omitted for clarity.

Details for the analysis of $[Pd_6(L2)_{12}](BF_4)_{12}$:

A colorless plate-shaped crystal with dimensions 0.16 × 0.12 × 0.03 mm^3 was mounted. Data were collected using a XtaLAB Synergy R, DW system, HyPix-Arc 150 diffractometer operating at T = 140.00(10) K. Data were measured using ω scans with Cu K_α radiation. The diffraction pattern was indexed and the total number of runs and images was based on the strategy calculation from the program CrysAlis^{Pro}.[S7] The maximum resolution achieved was Θ = 74.887°. The unit cell was refined using CrysAlis^{Pro} on 12412 reflections, 34% of the observed reflections.

Data reduction, scaling and absorption corrections were performed using CrysAlis^{Pro}. The final completeness is 99.90 % out to 74.887° in Θ. A Gaussian absorption correction was performed using CrysAlis^{Pro}. Numerical absorption correction based on Gaussian integration over a multifaceted crystal model. Empirical absorption correction using spherical harmonics as implemented in SCALE3 ABSPACK scaling algorithm. The absorption coefficient μ of this material is 6.826 mm^{-1} at this wavelength (λ = 1.54184 Å) and the minimum and maximum

transmissions are 0.379 and 1.000. The structure was solved in the space group *I*2 (# 5) by ShelXT[S8] using dual methods, Olex2[S9] as the graphical interface and was refined by full matrix least squares minimization on $|F|^2$ using ShelXL.[S10] All non-hydrogen atoms were refined anisotropically. Hydrogen atom positions were calculated geometrically and refined using the riding model.

The model is refined as a 2-component inversion twin. A solvent mask was calculated and 418 electrons were found in a volume of 1658 Å^3 in 3 voids per unit cell. This is consistent with the presence of ten solvent molecules of acetonitrile per Asymmetric Unit which account for 440 electrons per unit cell. The value of Z' is 0.5. This means that only half of the formula unit is present in the asymmetric unit, with the other half consisting of symmetry equivalent atoms. The moiety formula is $C_{84}H_{72}N_{48}Pd_6$, 12(BF_4), 2(C_2H_3N), 10[C_2H_3N].

The Flack parameter was refined to 0.51(2). Determination of absolute structure using Bayesian statistics on Bijvoet differences using the Olex2 results in 0.118(2). There are no chiral atoms in this structure. Note: The Flack parameter is used to determine chirality of the crystal studied, the value should be near 0, a value of 1 means that the stereochemistry is wrong and the model should be inverted. A value of 0.5 means that the crystal consists of a racemic mixture of the two enantiomers.

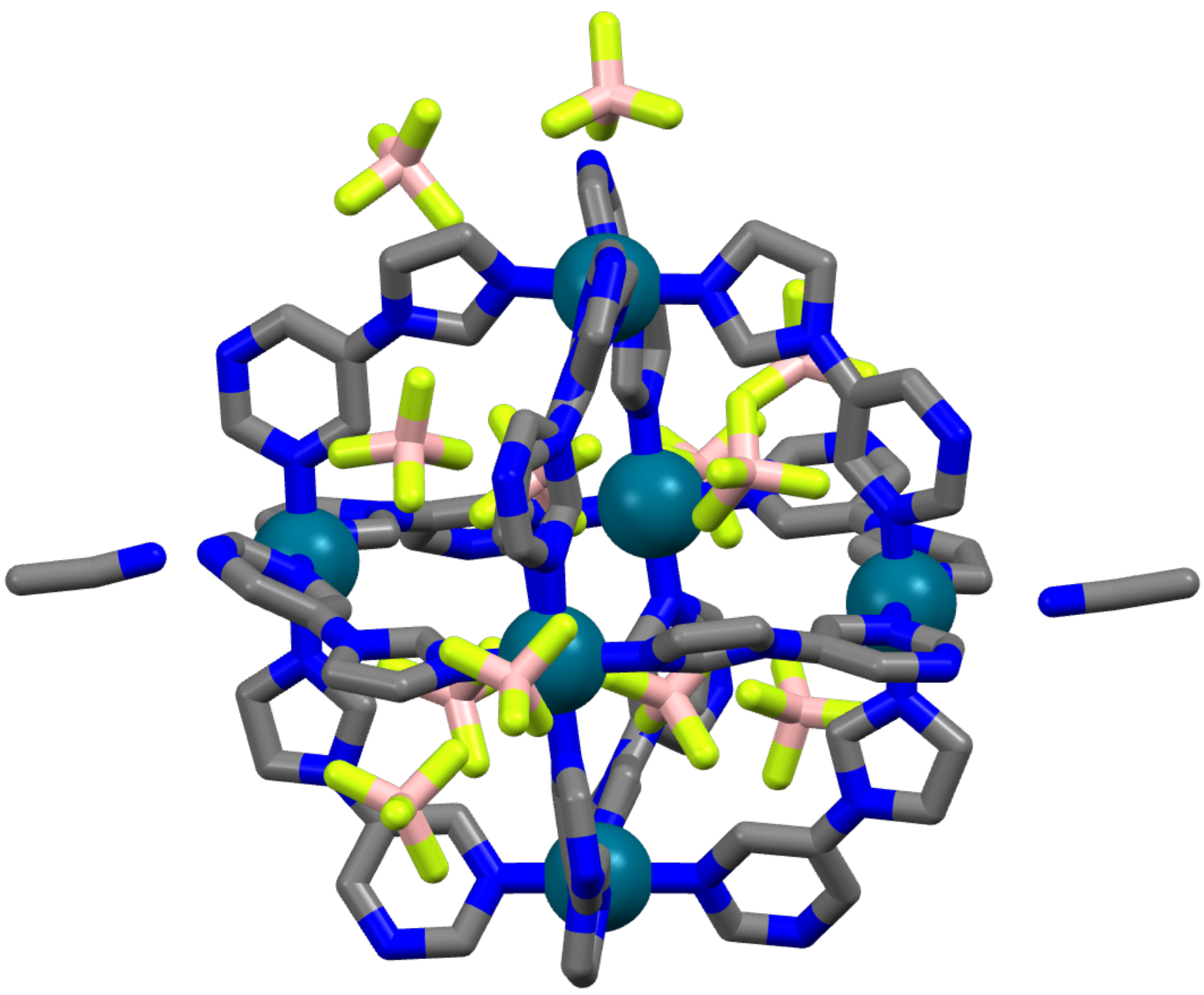

Figure S64. Structure of $[Pd_6(\mathbf{L2})_{12}]^{12+}$ with twelve BF_4^- anions and two acetonitrile solvent molecules as determined by single-crystal XRD. The hydrogen atoms are omitted for clarity.

Details for the analysis of $[Pd_6(\mathbf{L3})_{12}](BF_4)_{12}$:

A colorless, cube-shaped crystal with dimensions 0.24 × 0.21 × 0.13 mm^3 was mounted. Data were collected using a XtaLAB Synergy R, DW system, HyPix-Arc 150 diffractometer operating at T = 140.00(10) K.Data were measured using ω scans with Cu K_α radiation. The diffraction pattern was indexed and the total number of runs and images was based on the strategy calculation from the program CrysAlis^{Pro}.[S7] The maximum resolution achieved was Θ = 50.425°. The unit cell was refined using CrysAlis^{Pro} on 7133 reflections, 32% of the observed reflections.

Data reduction, scaling and absorption corrections were performed using CrysAlis^{Pro}. The final completeness is 99.50 % out to 50.425° in Θ. A Gaussian absorption correction was performed using CrysAlis^{Pro}. Numerical absorption correction based on Gaussian integration over a multifaceted crystal model. Empirical absorption correction using spherical harmonics as implemented in SCALE3 ABSPACK scaling algorithm. The absorption coefficient μ of this material is 6.167 mm^{-1} at this wavelength (λ = 1.54184Å) and the minimum and maximum transmissions are 0.309 and 0.743. The structure was solved in the space group $Fm\bar{3}c$ (# 226) by ShelXT [S8] using dual methods, Olex2[S9] as the graphical interface and was refined by full matrix least squares minimization on $|F|^2$ using ShelXL.[S10] All non-hydrogen atoms were refined anisotropically. Hydrogen atom positions were calculated geometrically and refined using the riding model.

A solvent mask was calculated, and 9328 electrons were found in a volume of 47 926 Å³ in two voids per unit cell. This is consistent with the presence of 8 $[BF_4]^-$ per asymmetric unit, which account for 7872 electrons per unit cell. The value of Z' is 0.125. The moiety formula is $C_{84}H_{72}N_{48}Pd_6$, 12$[BF_4]$.

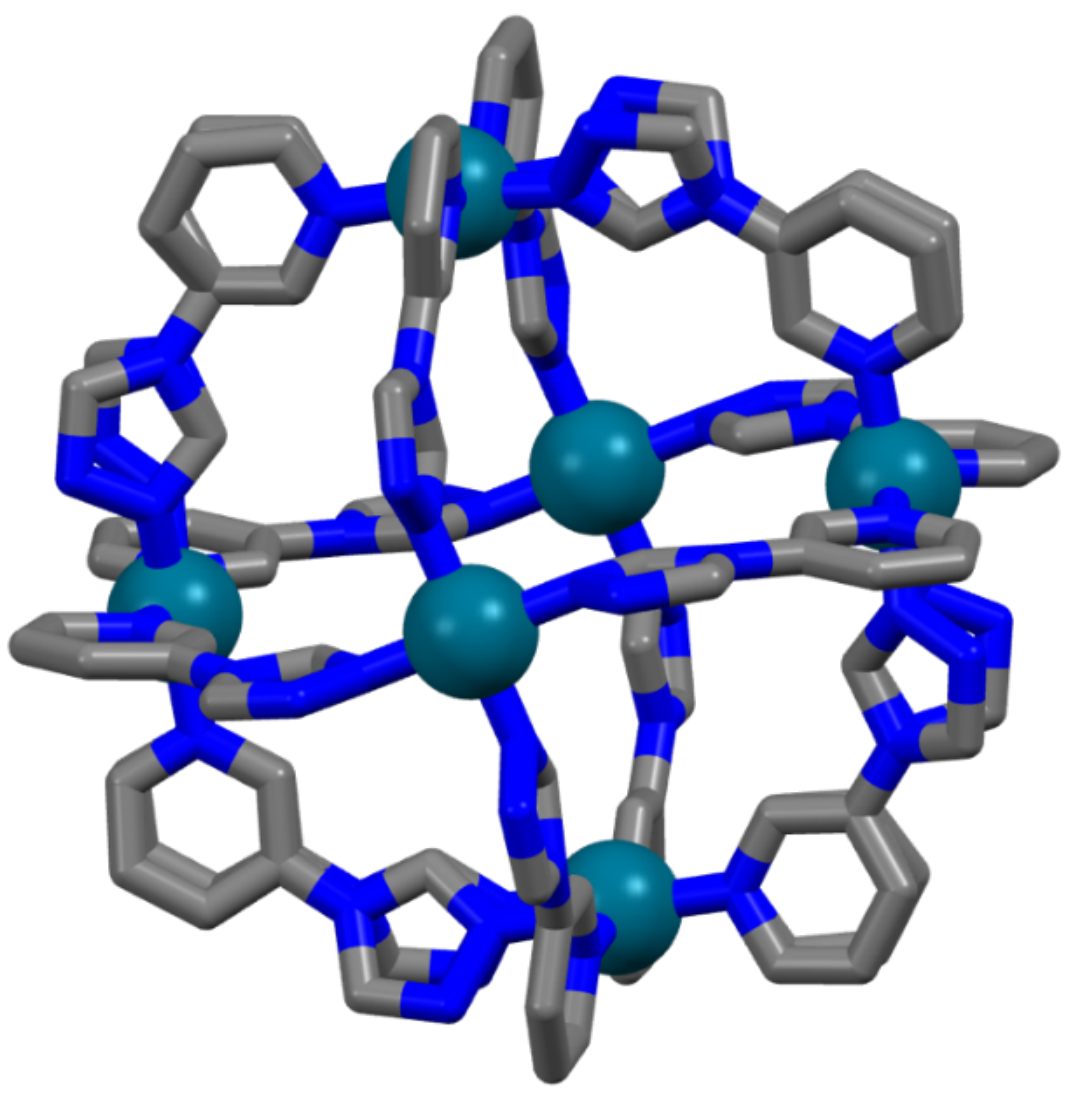

Figure S65. Structure of $[Pd_6(\mathbf{L3})_{12}]^{12+}$ as determined by single-crystal XRD. The hydrogen atoms are omitted for clarity.

Details for the analysis of $[Pt_2Pd_6(L1)_8Cl_8](BF_4)_4$:

A yellow prism-shaped crystal with dimensions 0.38 × 0.07 × 0.05 mm^3 was mounted. Data were collected using a XtaLAB Synergy R, DW system, HyPix-Arc 150 diffractometer operating at T = 100.00(10) K.Data were measured using ω scans with Mo K_α radiation. The diffraction pattern was indexed and the total number of runs and images was based on the strategy calculation from the program CrysAlis^{Pro}.[S7] The maximum resolution achieved was Θ = 30.508°. The unit cell was refined using CrysAlis^{Pro} on 23017 reflections, 27% of the observed reflections.

Data reduction, scaling and absorption corrections were performed using CrysAlis^{Pro}. The final completeness is 99.90 % out to 30.508° in Θ. A Gaussian absorption correction was performed using CrysAlis^{Pro}. Numerical absorption correction based on Gaussian integration over a multifaceted crystal model. Empirical absorption correction using spherical harmonics as implemented in SCALE3 ABSPACK scaling algorithm. The absorption coefficient μ of this material is 3.241 mm^{-1} at this wavelength (λ = 0.71073Å) and the minimum and maximum transmissions are 0.482 and 1.000. The structure was solved in the space group $P2_1/c$ (# 14) by ShelXT[S8] using dual methods, Olex2[S9] as the graphical interface and was refined by full matrix least squares minimization on $|F|^2$ using ShelXL.[S10] All non-hydrogen atoms were refined anisotropically. Hydrogen atom positions were calculated geometrically and refined using the riding model. A solvent mask was calculated and 664 electrons were found in a volume of 2205$Å^3$ in 1 void per unit cell. This is consistent with the presence of $15[C_2H_3N]$ per Asymmetric Unit which account for 660 electrons per unit cell. The value of Z' is 0.5. This means that only half of the formula unit is present in the asymmetric unit, with the other half consisting of symmetry equivalent atoms. The moiety formula is $C_{64}H_{56}Cl_8N_{24}Pd_4Pt_2$, $4(BF_4)$, $15[C_2H_3N]$.

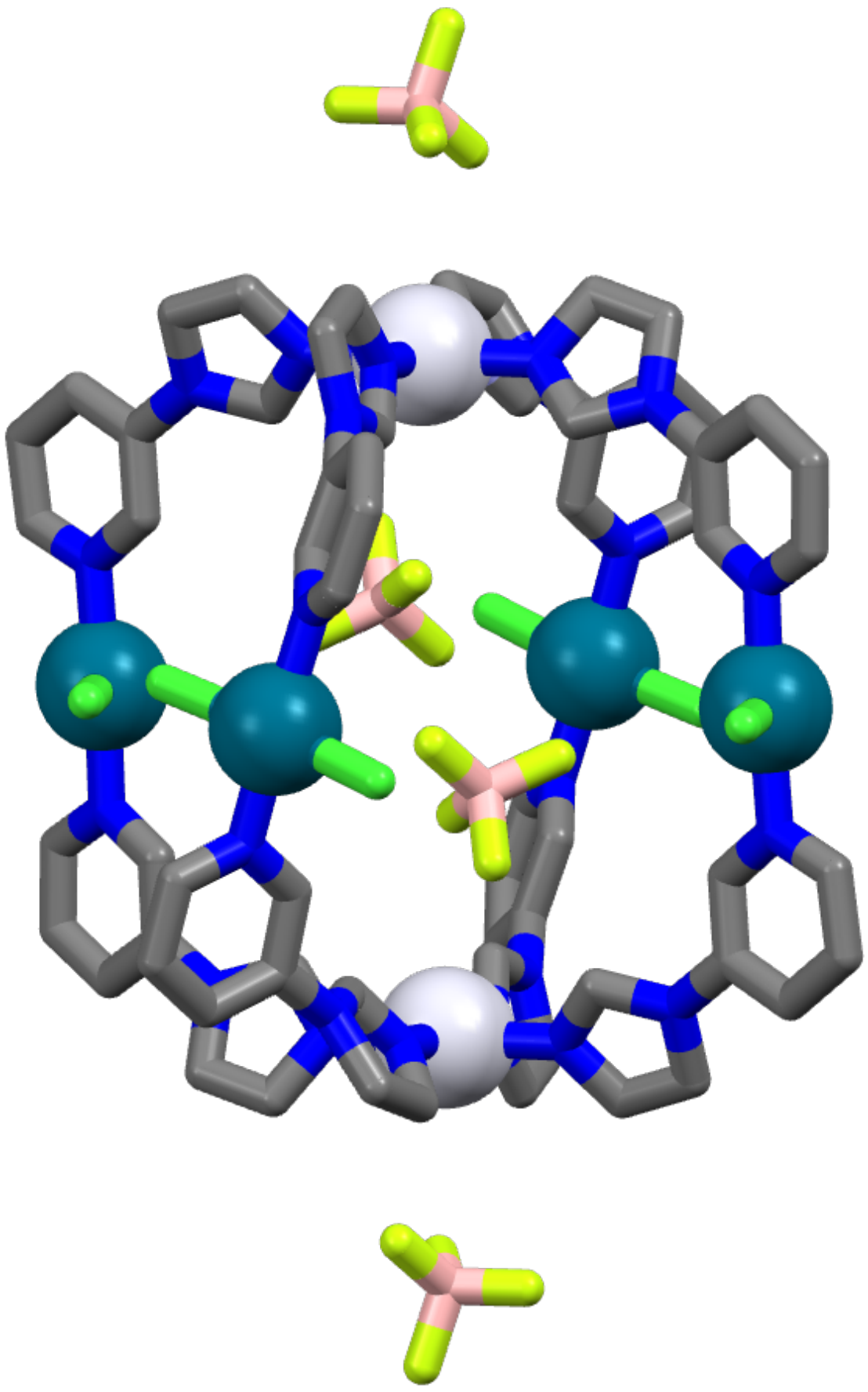

Figure S66. Structure of $[Pt_2Pd_4(\mathbf{L1})_8Cl_8]^{4+}$ with four BF_4^- anions as determined by single-crystal XRD. The hydrogen atoms are omitted for clarity.

Details for the analysis of $[Pt_2Pd_6(L2)_8Cl_8](BF_4)_4$:

A yellow block-shaped crystal with dimensions 0.09 × 0.08 × 0.06 mm^3 was mounted. Data were collected using a XtaLAB Synergy R, DW system, HyPix-Arc 150 diffractometer operating at T = 99.98(16) K.Data were measured using ω scans with Cu K_α radiation. The diffraction pattern was indexed and the total number of runs and images was based on the strategy calculation from the program CrysAlis^{Pro}.[S7] The maximum resolution achieved was Θ = 75.659°. The unit cell was refined using CrysAlis^{Pro} on 15506 reflections, 26% of the observed reflections.

Data reduction, scaling and absorption corrections were performed using CrysAlis^{Pro}. The final completeness is 99.90 % out to 75.659° in Θ. A Gaussian absorption correction was performed using CrysAlis^{Pro}. Numerical absorption correction based on Gaussian integration over a multifaceted crystal model. Empirical absorption correction using spherical harmonics as implemented in SCALE3 ABSPACK scaling algorithm. The absorption coefficient μ of this material is 9.631 mm^{-1} at this wavelength (λ = 1.54184 Å) and the minimum and maximum transmissions are 0.515 and 0.685. The structure was solved in the space group $I4/m$ (# 87)

by ShelXT [S8] using dual methods, Olex2[S9] as the graphical interface and was refined by full matrix least squares minimization on $|F|^2$ using ShelXL.[S10] All non-hydrogen atoms were refined anisotropically. Hydrogen atom positions were calculated geometrically and refined using the riding model. The value of Z' is 0.125. The moiety formula is $C_{56}H_{48}Cl_8N_{32}Pd_4Pt_2$, $3(BF_4)$, NO_3, $8(CH_3NO_2)$, [+ solvents].

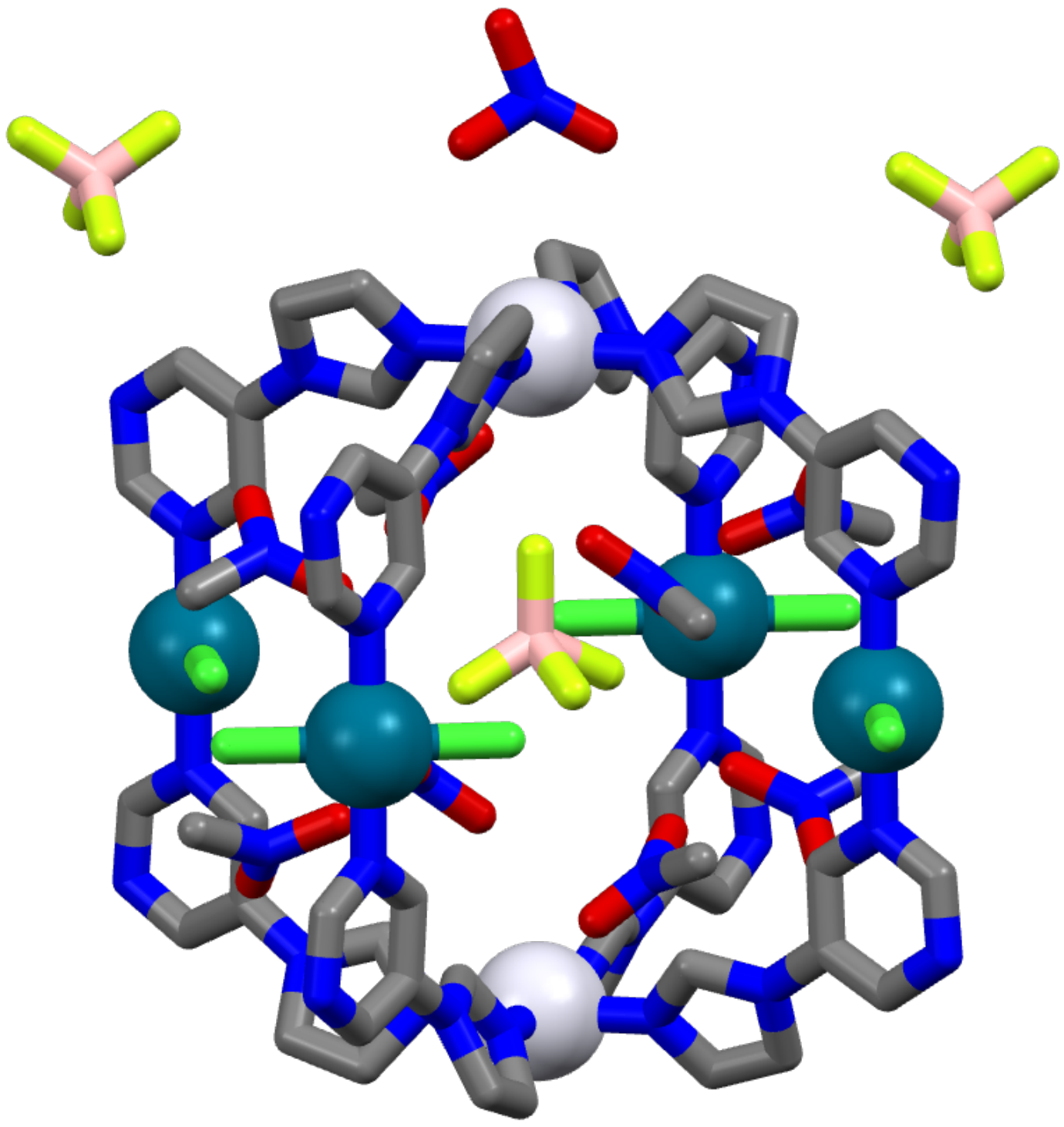

Figure S67. Structure of $[Pt_2Pd_4(\mathbf{L2})_8Cl_8]^{4+}$ with three BF_4^- anions, one NO_3^- anion and eight nitromethane solvent molecules as determined by single-crystal XRD. The hydrogen atoms are omitted for clarity.

Table S1. Crystal data and structure refinement for [Pt(**L1**)$_4$](BF$_4$)$_2$, [Pt(**L2**)$_4$](BF$_4$)$_2$ and [Pt(**L3**)$_4$](BF$_4$)$_2$.

**Compound**	Pt(**L1**)$_4$](BF$_4$)$_2$	[Pt(**L2**)$_4$](BF$_4$)$_2$	[Pt(**L3**)$_4$](BF$_4$)$_2$
Formula	$C_{39}H_{36}B_2F_8N_{12}Pt$	$C_{35}H_{32}B_2F_8N_{16}Pt$	$C_{38.5}H_{36}B_2F_8N_{16}Pt$
$D_{calc.}$/ g cm^{-3}	1.691	1.667	1.701
μ/mm^{-1}	7.146	3.452	3.380
Formula Weight	1041.51	1045.47	1091.54
Colour	colorless	colorless	colorless
Shape	needle-shaped	prism-shaped	prism-shaped
Size/mm^3	0.14×0.03×0.02	0.16×0.12×0.05	0.29×0.11×0.05
T/K	140.00(10)	140.00(10)	140.00(10)
Crystal System	triclinic	monoclinic	triclinic
Space Group	$P\bar{1}$	$P2_1/c$	$P\bar{1}$
a/Å	8.5401(2)	12.96301(18)	10.90071(10)
b/Å	9.9681(3)	8.91998(11)	14.15927(13)
c/Å	13.0801(3)	18.8610(3)	15.18768(9)
α/°	101.605(2)	90	112.5544(7)
β/°	98.795(2)	107.1977(16)	97.4732(6)
γ/°	105.978(3)	90	93.5221(7)
V/Å^3	1022.62(5)	2083.39(5)	2130.56(3)
Z	1	2	2
Z'	0.5	0.5	1
Wavelength/Å	1.54184	0.71073	0.71073
Radiation type	Cu K_α	Cu K_α	Mo K_α
Θ_{min}/°	3.537	1.644	1.674
Θ_{max}/°	74.127	30.507	36.316
Index range h	-10 ≤ h ≤ 10	-18 ≤ h ≤ 18	-18 ≤ h ≤ 17
Index range k	-12 ≤ k ≤ 12	-12 ≤ k ≤ 12	-22 ≤ k ≤ 23
Index range l	-12 ≤ l ≤ 16	-26 ≤ l ≤ 25	-25 ≤ l ≤ 25
Measured Refl's.	15806	42711	150307
Indep't Refl's	3967	6361	20648
Refl's I≥2σ(I)	3940	4897	18703
R_{int}	0.0457	0.0287	0.0241
Parameters	392	394	682
Restraints	338	420	407
Largest Peak/e Å^{-3}	1.017	1.793	1.484
Deepest Hole/e Å^{-3}	-1.860	-0.647	-1.061
GooF	1.098	1.117	1.056
wR_2	0.0354 / 0.0361	0.0298 / 0.0416	0.0231 / 0.0284
R_1	0.0772 / 0.0783	0.0788 / 0.0841	0.0538 / 0.0552
CCDC number	**2541156**	**2541157**	**2541158**

Table S2. Crystal data and structure refinement for $[Pd_6(\mathbf{L1})_{12}](BF_4)_{12}$, $[Pd_6(\mathbf{L1})_{12}](OTf)_{12}$ and $[Pd_6(\mathbf{L2})_{12}](BF_4)_{12}$.

**Compound**	$[Pd_6(\mathbf{L1})_{12}](BF_4)_{12}$	$[Pd_6(\mathbf{L1})_{12}](OTf)_{12}$	$[Pd_6(\mathbf{L2})_{12}](BF_4)_{12}$
Formula	$C_{96}H_{84}B_{12}F_{48}N_{36}Pd_6$	$C_{103}H_{80}F_{21}N_{36}O_{21}Pd_6S_7$	$C_{108}H_{108}B_{12}F_{48}N_{60}Pd_6$
$D_{calc.}$/ g cm^{-3}	1.311	1.340	1.740
μ/mm^{-1}	5.781	6.566	6.826
Formula Weight	3422.11	3419.85	3926.66
Colour	colorless	colorless	colorless
Shape	prism-shaped	irregular-shaped	plate-shaped
Size/mm^3	0.15×0.15×0.07	0.09×0.05×0.03	0.16×0.12×0.03
T/K	229.99(10)	140.00(10)	140.00(10)
Crystal System	trigonal	monoclinic	monoclinic
Flack Parameter	-	-	0.51(2)
Hooft Parameter	-	-	0.118(2)
Space Group	$R\bar{3}c$	$C2/c$	$I2$
a/Å	20.8268(3)	30.5509(6)	17.9777(2)
b/Å	20.8268(3)	22.4198(4)	21.2678(4)
c/Å	69.2165(10)	24.8408(3)	19.9935(5)
α/°	90	90	90
β/°	90	94.6944(15)	101.4064(18)
γ/°	120	90	90
V/Å^3	26000.6(8)	16957.5(5)	7493.4(3)
Z	6	4	2
Z'	0.166667	0.5	0.5
Wavelength/Å	1.54184	1.54184	1.54184
Radiation type	Cu K_α	Cu K_α	Cu K_α
Θ_{min}/°	2.763	2.447	3.023
Θ_{max}/°	74.890	39.970	74.887
Index range h	-24 ≤ h ≤ 25	-25 ≤ h ≤ 25	-16 ≤ h ≤ 22
Index range k	-25 ≤ k ≤ 19	-17 ≤ k ≤ 18	-26 ≤ k ≤ 26
Index range l	-82 ≤ l ≤ 85	-20 ≤ l ≤ 20	-24 ≤ l ≤ 23
Measured Refl's.	66169	30231	36083
Indep't Refl's	5878	5115	13045
Refl's I≥2σ(I)	5126	3979	8422
R_{int}	0.0232	0.0394	0.0191
Parameters	249	784	871
Restraints	489	1509	1789
Largest Peak/e Å^{-3}	1.956	2.399	2.055
Deepest Hole/e Å^{-3}	-1.621	-1.655	-0.881
GooF	1.693	2.203	1.136
wR_2	0.1112 / 0.1155	0.1478 / 0.1732	0.0946 / 0.1208
R_1	0.3563 / 0.3628	0.4300 / 0.4621	0.2695 / 0.3038
CCDC number	**2541152**	**2541154**	**2541155**

Table S3. Crystal data and structure refinement for $[Pd_6(\mathbf{L3})_{12}](BF_4)_{12}$, $[Pt_2Pd_4(\mathbf{L1})_8Cl_8](BF_4)_4$ and $[Pt_2Pd_4(\mathbf{L2})_8Cl_8](BF_4)_4$.

**Compound**	$[Pd_6(\mathbf{L3})_{12}](BF_4)_{12}$	$[Pt_2Pd_4(\mathbf{L1})_8Cl_8](BF_4)_4$	$[Pt_2Pd_4(\mathbf{L2})_8Cl_8](BF_4)_4$
Formula	$C_{84}H_{72}B_{12}F_{48}N_{48}Pd_6$	$C_{94}H_{101}B_4Cl_8F_{16}N_{39}Pd_4Pt_2$	$C_{64}H_{72}B_3Cl_8F_{12}N_{41}O_{19}Pd_4Pt_2$
$D_{calc.}$/ g cm^{-3}	1.398	1.822	1.439
μ/mm^{-1}	6.167	3.241	9.631
Formula Weight	3434.01	3223.75	3079.43
Colour	colorless	yellow	yellow
Shape	cube-shaped	prism-shaped	block-shaped
Size/mm^3	0.24×0.21×0.13	0.38×0.07×0.05	0.09×0.08×0.06
T/K	140.00(10)	100.00(10)	99.98(16)
Crystal System	cubic	monoclinic	tetragonal
Space Group	$Fm\bar{3}c$	$P2_1/c$	$I4/m$
a/Å	46.0840(3)	13.2498(4)	14.8530(2)
b/Å	46.0840(3)	31.4320(8)	14.8530(2)
c/Å	46.0840(3)	15.0723(4)	32.2196(6)
α/°	90	90	90
β/°	90	110.601(3)	90
γ/°	90	90	90
V/Å^3	97870.2(19)	5875.8(3)	7108.0(2)
Z	24	2	2
Z'	0.125	0.5	0.125
Wavelength/Å	1.54184	0.71073	1.54184
Radiation type	Cu K_α	Mo K_α	Cu K_α
Θ_{min}/°	4.290	1.879	2.743
Θ_{max}/°	50.425	30.508	75.659
Index range h	-21 ≤ h ≤ 34	-18 ≤ h ≤ 18	-18 ≤ h ≤ 14
Index range k	-38 ≤ k ≤ 45	-44 ≤ k ≤ 44	-18 ≤ k ≤ 18
Index range l	-19 ≤ l ≤ 45	-17 ≤ l ≤ 21	-40 ≤ l ≤ 39
Measured Refl's.	22181	83886	59461
Indep't Refl's	2252	17921	3750
Refl's I≥2σ(I)	1508	11891	3342
R_{int}	0.0193	0.0552	0.0483
Parameters	166	578	239
Restraints	344	84	90
Largest Peak/e Å^{-3}	3.967	1.725	1.903
Deepest Hole/e Å^{-3}	-0.870	-0.693	-2.545
GooF	3.063	0.991	1.076
wR_2	0.2551 / 0.2717	0.0486 / 0.0737	0.0647 / 0.0703
R_1	0.6081 / 0.6375	0.1312 / 0.1423	0.1908 / 0.1969
CCDC number	**2541153**	**2541159**	**2541160**

6 References

(S1) Li, R.-J.; Tarzia, A; Posligua, V.; Jelfs, K. E.; Sanchez, N.; Marcus, A.; Baksi, A.; Clever, G. H.; Fadaei-Tirani, F.; Severin, K. Orientational self-sorting in cuboctahedral Pd cages. *Chem. Sci*. **2022**, *13*, 11912–11917.

(S2) Wiley, R. H.; Hart, A. J. Reaction of Diformylhydrazine with Aminoheterocycles. *J. Org. Chem.* **1953,** *18, 1368*–1371.

(S3) Huang, G.; Yang, L.; Yin, Q.; Fang, Z.-B.; Hu, X.-J. ; Zhang, A.-A.; Jiang, J.; Liu, T.-F.; Cao, R. A Comparison of Two Isoreticular Metal-Organic Frameworks with Cationic and Neutral Skeletons: Stability, Mechanism, and Catalytic Activity. *Angew. Chem. Int. Ed.*, **2020**, 59, 4385.

(S4) MassLynx Mass Spectrometry Software, Waters Corporation, Wilmslow, UK. (V4.2 SCN1016 Release 10)

(S5) R Core Team (2025) R: A Language and Environment for Statistical Computing. R Foundation for Statistical Computing, Vienna, Austria. (https://www.R-project.org/)

(S6) Gier, E. C.; Leontyev, D.; Fernández, F. M.; Multipass CCS Refiner. *J. Am. Soc. Mass Spectrom.* **2025**, 36, 9, 2000. (freely available at https://ericgier.shinyapps.io/Multipass-CCS-Refiner/)

(S7) CrysAlis^{Pro} Software System Rigaku Oxford Diffraction (1.171.42.75a), **2022**.

(S8) Sheldrick, G. M. ShelXT-Integrated space-group and crystal-structure determination, *Acta Cryst.*, **2015**, *A71*, 3–8.

(S9) Dolomanov, O. V.; Bourhis, J. L.; Gildea, R. J.; Howard, J. A. K.; Puschmann, H.; Olex2: A complete structure solution, refinement and analysis program. *J. Appl. Cryst.*, **2009**, *42*, 339–341.

(S10) Sheldrick, G. M. Crystal structure refinement with ShelXL (version 2019/3), *Acta Cryst.*, **2015**, *C71*, 3–8.
